# Hospital-to-Home Neurological Transition Care: A Scoping Review Across Selected Chronic Neurological Disorders

**DOI:** 10.3390/medsci14040445

**Published:** 2026-07-28

**Authors:** Rocco Salvatore Calabrò, Andrea Calderone, Daniele Ravi, Carmelo Galipò, Maria Felicita Crupi, Angelo Quartarone

**Affiliations:** 1 IRCCS Centro Neurolesi Bonino Pulejo, 98124 Messina, Italy; roccos.calabro@irccsme.it (R.S.C.); maria.crupi@irccsme.it (M.F.C.); angelo.quartarone@irccsme.it (A.Q.); 2 Department of Mathematics, Computer Science, Physics and Earth Sciences (MIFT), University of Messina, 98166 Messina, Italy; daniele.ravi@unime.it (D.R.); info@carmelogalipo.it (C.G.)

**Keywords:** transitional care, post-discharge follow-up, continuity of care, selected chronic neurological disorders, Parkinson’s disease, dementia, multiple sclerosis, amyotrophic lateral sclerosis

## Abstract

Background: Returning home after neurological hospitalization, rehabilitation, or specialist care transfers responsibility to patients, caregivers, and community services. We mapped mechanisms and gaps across dementia/Alzheimer’s disease and related dementias (ADRD), Parkinson’s disease (PD), multiple sclerosis (MS), and amyotrophic lateral sclerosis (ALS). Methods: Following JBI guidance and PRISMA-ScR, eligibility was derived using population–concept–context. We included empirical reports involving adults with a target condition, a post-discharge, return-home, rehabilitation, telehealth, caregiver, treatment, respiratory, or palliative continuity component, and post-transition patient, caregiver, service, safety, rehabilitation, equity, or implementation outcomes. Five databases were searched through to 11 May 2026. Two reviewers independently screened records; charting and classification were verified by R.S.C., A.C., and A.Q. Results: Of 24,417 records, 69 reports were included: Dementia/ADRD, 28; PD, 10; MS, 9; and ALS, 22. Eighteen were core transition reports (26.1%), 14 return-home/community re-entry reports (20.3%), 16 adjacent continuity reports (23.2%), and 21 companion/secondary reports (30.4%). Dementia/ADRD provided discharge-anchored evidence; PD and MS mapped functional carry-over; ALS mapped adjacent respiratory, telehealth, and palliative continuity. Conclusions: The main contribution is an operational cross-disease framework separating direct discharge, return-home, adjacent-continuity, and companion evidence while linking mechanisms to disease-specific pathways. This framework maps disease-specific functions, not comparative effectiveness. The proposed frameworks are author-derived and hypothesis-generating. Future studies should use explicit anchors, standardized outcomes, longer follow-up, and equity-sensitive implementation measures addressing caregiver workload, digital access, feasibility, and sustainability. They inform testable, context-sensitive intervention designs for future neurological transition-care research and practice.

## 1. Introduction

Neurological disorders account for a major and increasing share of global health loss, and many chronic neurological conditions require care trajectories that extend well beyond acute hospital treatment or specialist rehabilitation [1]. For people living with progressive or fluctuating neurological disability, the end of an inpatient episode does not represent the end of clinical need. It often marks the point at which responsibility for medication routines, rehabilitation exercises, functional adaptation, symptom monitoring, equipment use, and service navigation shifts rapidly toward patients, families, primary care, outpatient services, and community providers.

The transition from hospital, subacute care, specialist care, or inpatient rehabilitation to home is therefore a clinically exposed interval. Information may be lost across services, medication schedules may change, rehabilitation plans may be incompletely transferred, and new functional limitations may become apparent only when the person resumes everyday activities. Transitional care has been conceptualized as a coordinated set of actions intended to preserve continuity, safety, communication, and accountability when patients move between settings [2,3]. In complex chronic illness, this process requires more than a discharge letter. It involves anticipatory planning, medication reconciliation, caregiver preparation, rehabilitation continuity, timely follow-up, and active linkage among specialist, primary-care, rehabilitation, and community services [4]. Hospital-initiated transitional-care and readmission-prevention strategies may be beneficial in selected contexts, but their effects vary according to population, intervention content, outcome selection, and implementation environment [5,6].

For this review, the post-discharge transition is understood as a trajectory rather than a single administrative event. Post-discharge follow-up refers to structured or inferable clinical, rehabilitation, nursing, telehealth, caregiver-facing, or service-navigation contact after discharge. Transitional care refers to a coordinated model designed to bridge settings and responsibilities. Return home refers both to the physical discharge destination and to the early period in which patients and caregivers re-establish daily routines, manage residual disability, and interact with services outside the institution. Community re-entry refers to the broader process of resuming participation, self-management, social roles, outpatient care, and community-based rehabilitation after discharge. These concepts overlap but are not interchangeable; separating them is necessary to understand what each study actually evaluates.

Parkinson’s disease (PD) exemplifies progressive neurodegenerative disability because motor and non-motor symptoms, cognitive and autonomic changes, dysphagia, falls, medication complexity, and fluctuating function may compromise discharge readiness and home stability [7,8]. Hospitalization adds risks of dopaminergic medication interruption or mistiming, delirium, aspiration, mobility decline, comorbidity, and polypharmacy [9]. Safe return home therefore requires accurate medication transfer, caregiver education, rehabilitation continuity, falls prevention, and timely neurological support. Integrated patient-centred networks and remote follow-up may extend specialist care, but depend on disease stage, digital access, infrastructure, and caregiver capacity [10,11,12].

Dementia/Alzheimer’s disease and related dementias (ADRD) create a different but equally complex transition-of-care challenge. Cognitive impairment, behavioural and psychological symptoms, frailty, multimorbidity, communication barriers, and impaired capacity for self-management can make discharge planning highly dependent on informal caregivers and community services [13]. Reviews of hospital discharge procedures have identified persistent gaps in continuity for people with dementia, especially when information transfer, caregiver preparation, medication management, and post-discharge support are insufficiently specified [14]. Planned care transitions for persons with dementia have been described across heterogeneous models, including discharge planning, case management, follow-up calls, caregiver-directed interventions, and multidisciplinary coordination, but study populations, settings, intervention components, and outcome measures differ substantially [15]. Medication management after discharge is particularly caregiver-mediated, and dementia-specific interventions have addressed caregiver guidance with variable depth and structure [16]. Evidence in this area therefore needs to be interpreted through patient safety, caregiver burden, preparedness, service navigation, proxy reporting, and continuity of responsibility after formal care ends.

MS is a chronic immune-mediated neuroinflammatory disease with neurodegenerative features and long-term disability accumulation [17]. Its increasing global burden and relapsing or progressive trajectories often require repeated contact with inpatient rehabilitation, outpatient neurology, community rehabilitation, home services, and self-management support [18]. Although MS rehabilitation spans physical, cognitive, multidisciplinary, and self-management domains, whether inpatient gains persist at home remains a distinct transition question [19,20]. Studies of rehabilitation carry-over, home-based management, telerehabilitation, and remote coaching highlight post-discharge reinforcement, fatigue management, functional carry-over, participation, and quality of life, while showing that intervention targets and outcomes remain diverse [21,22,23,24,25].

Amyotrophic lateral sclerosis (ALS) provides a complementary transition phenotype. Rapidly progressive motor decline, respiratory compromise, dysphagia, communication loss, and dependence on assistive technologies can alter care requirements over short intervals. Continuity therefore extends beyond a single discharge episode to repeated transitions involving home-based specialist input, non-invasive ventilation and other respiratory support, nutritional management, caregiver training, telemonitoring, palliative care, advance care planning, and end-of-life decision-making. Including ALS allows the review to examine whether principles identified in more conventionally discharge-anchored populations remain relevant when specialist, technological, and palliative support must be sustained across an evolving home-care trajectory.

PD, dementia/ADRD, MS and ALS were selected as a deliberately bounded set of clinically consequential chronic neurological conditions rather than as an exhaustive representation of neurological disease. Together, they span progressive neurodegenerative, immune-mediated, rehabilitation-intensive, respiratory and palliative trajectories; frequently require transfer from hospital, inpatient rehabilitation, skilled nursing, specialist or multidisciplinary care to home and community settings; and make caregiver work, medication or treatment continuity, rehabilitation carry-over, monitoring and escalation, and service access central to safety. Preliminary scoping also indicated sufficient transition-related literature to permit cross-disease comparison. Other conditions, including Huntington’s disease, atypical parkinsonism, epilepsy, stroke, traumatic brain injury, spinal cord injury and generic frailty, were excluded to preserve conceptual coherence and avoid adding disease pathways for which the preliminary corpus did not support stable evidence classification.

Previous syntheses have examined important parts of this landscape, including hospitalization risks and integrated care in PD [9,10], discharge procedures, planned care transitions, and caregiver medication support in dementia [14,15,16], and multidisciplinary rehabilitation or telerehabilitation in MS [19,20,23]. These reviews are informative but are generally disease-specific, intervention-specific, or effectiveness-oriented. They do not jointly map how discharge-anchored studies, return-home trajectories, rehabilitation carry-over, remote continuity, caregiver work, respiratory support, and palliative continuity are distributed across chronic neurological conditions. Nor do they provide a cross-disease framework for distinguishing direct transition evidence from adjacent continuity research and overlapping companion reports.

The literature also differs substantially in transition anchor, setting, study design, comparator, follow-up duration, intervention content, and outcome definition. Given this heterogeneity, a scoping review was considered more appropriate than a pooled effectiveness synthesis because the objective was to map concepts, mechanisms, care models, and evidence gaps rather than estimate a single comparative effect [26,27,28,29,30,31,32]. This mapping also provides the preliminary work needed before future systematic reviews can test narrower effectiveness questions with standardized outcomes, explicit transition anchors, and comparable follow-up periods. The objective of this scoping review was therefore to map published evidence on post-discharge follow-up, transitional care, return-home support, rehabilitation continuity, and community re-entry in adults with PD, dementia/ADRD, MS, or ALS, while developing an evidence-classification framework and identifying cross-cutting and disease-specific continuity mechanisms and gaps.

## 2. Materials and Methods

### 2.1. Review Design and Reporting Framework

A scoping review was selected because the objective was to characterize the breadth, concepts, care models, and evidence gaps in a heterogeneous literature rather than estimate a pooled intervention effect. The review was informed by the frameworks of Arksey and O’Malley and Levac and colleagues, Joanna Briggs Institute (JBI) guidance, the Preferred Reporting Items for Systematic Reviews and Meta-Analyses Extension for Scoping Reviews (PRISMA-ScR), PRISMA 2020, and recommendations for scoping-review extraction and presentation [26,27,28,29,30,31,32,33,34,35]. The protocol was developed a priori and registered on the Open Science Framework (OSF) on 8 May 2026, before completion of final study selection, charting, and synthesis. The OSF registration number is US27J, and the registration record is available at the following address: https://doi.org/10.17605/OSF.IO/US27J.

Meta-analysis, risk-of-bias-based exclusion, and certainty grading were not planned because the evidence varied in disease population, transition anchor, setting, intervention structure, comparator, follow-up period, outcome measurement, and implementation context. Methodological features relevant to interpretation, including design, comparator use, sample size, follow-up completeness, and reporting clarity, were charted descriptively.

### 2.2. Population–Concept–Context Framework and Review Question

Eligibility and synthesis were organized primarily using the JBI population–concept–context (PCC) framework. The population comprised adults or adult-dominant samples with PD, dementia/ADRD, MS, or ALS, including mixed samples only when target-condition data were separable or clearly predominant. The four conditions were treated as selected chronic neurological populations with sufficient transition-related literature and complementary continuity needs, not as a comprehensive list of neurological disorders.

The concept comprised post-discharge follow-up, transitional care, discharge planning, return-home support, rehabilitation continuity, home or community re-entry, case management, telehealth or telerehabilitation, caregiver support, medication or treatment continuity, integrated care, respiratory or nutritional continuity, palliative care continuity, and advance care planning.

The context was a transition from acute hospital, inpatient rehabilitation, skilled nursing or subacute care, specialist or multidisciplinary care, or another organized neurological service to home, community, outpatient, primary-care, home-health, home-palliative, or everyday-life settings. Comparator and outcome fields derived from the population–intervention–comparator–outcomes (PICO) framework were retained only as auxiliary search and charting fields [36,37]. Comparator presence was recorded but was not required, and outcomes were mapped rather than used to restrict eligible study designs. Detailed operational definitions and boundary rules are provided in Supplementary Table S1.

The review question was as follows: What evidence has been published on the models, mechanisms, outcomes, and implementation features of post-discharge follow-up, transitional care, return-home support, rehabilitation continuity, and home or community re-entry for adults with PD, dementia/ADRD, MS, or ALS?

### 2.3. Eligibility Criteria

Eligibility criteria were defined a priori to ensure that included studies contributed empirical evidence on continuity after an organized episode of neurological care, rather than on general disease management alone. The criteria were applied to preserve the broad mapping purpose of the review while maintaining a clear boundary between transition-relevant evidence and studies without a post-discharge, return-home, home-based, outpatient, community-based, or care-continuity component. During screening, records were retained when transition relevance was plausible and were excluded at full-text assessment only when the target population, transition or continuity component, or post-transition outcome could not be established with sufficient clarity. The proximity of each study to a discharge or return-home pathway was subsequently handled through evidence stratification rather than used as a narrow inclusion filter.

#### 2.3.1. Inclusion Criteria

Eligible sources were peer-reviewed, English-language, full-text original articles reporting primary empirical data. We considered quantitative, qualitative, and mixed-methods studies, including randomized and non-randomized trials, quasi-experimental studies, prospective and retrospective cohort studies, before-after studies, observational studies, implementation studies, feasibility or acceptability studies, and service evaluations with empirical outcome data. No restriction was applied by publication year beyond the final database search date.

Studies were eligible when they included adults or adult-dominant samples with Parkinson’s disease, dementia/Alzheimer’s disease and related dementias, multiple sclerosis, or amyotrophic lateral sclerosis. Mixed neurological or mixed clinical samples were considered only when data for at least one target disorder were reported separately or when the eligible disorder was clearly predominant and directly relevant to the transition-care question. Reports with within-disease overlap, such as advanced PD with probable Parkinson’s disease dementia or ALS/MND/PLS/PMA samples, were classified according to the dominant eligible disease population and main continuity mechanism; these features are described in the population columns of the evidence tables. Dementia samples had to represent Alzheimer’s disease or another related neurodegenerative dementia syndrome, such as dementia with Lewy bodies, Parkinson’s disease dementia, frontotemporal dementia, mixed neurodegenerative dementia, or another clearly specified primary neurodegenerative dementia.

Eligible studies had to describe a post-discharge, return-home, home-based, outpatient, community-based, or continuity-of-care component after hospital care, inpatient rehabilitation, skilled nursing or subacute care, specialist care, multidisciplinary clinic care, palliative care, or another organized neurological-care episode. The transition anchor was coded as explicit when the report specified hospital discharge, inpatient rehabilitation discharge, SNF/subacute discharge, specialist-care episode completion, or home-health admission after hospitalization. It was coded as inferable when timing, pathway description, intervention start, follow-up schedule, or home/community destination clearly linked the care model to an organized neurological-care episode. Home-based, remote, respiratory, nutritional, caregiver, or palliative continuity without a discrete or clearly timed discharge point was retained when empirically relevant but classified as adjacent continuity evidence rather than direct core transition evidence.

Studies also had to report at least one outcome, process measure, or empirically described experience after the transition from organized care. Eligible outcomes included patient-centred, caregiver-centred, rehabilitation, safety, service-use, feasibility, acceptability, equity, implementation, respiratory, nutritional, palliative, or end-of-life outcomes. Examples included readmission, emergency department use, adverse events, falls, medication or treatment errors, activities of daily living, mobility, walking capacity, fatigue, participation, quality of life, caregiver burden, caregiver preparedness, caregiver strain, satisfaction, adherence, service use, technology usability, implementation barriers, access barriers, non-invasive ventilation adherence, symptom burden, advance care planning, and place-of-care or place-of-death outcomes.

Companion or secondary reports from the same intervention program, registry, or cohort were retained when they provided distinct information on mechanisms, outcomes, implementation, follow-up, subgroups, or caregiver-related dimensions relevant to the evidence map.

#### 2.3.2. Exclusion Criteria

Studies were excluded when they did not include one of the target neurological disorders or when eligible disease-specific data could not be separated from broader mixed samples. Studies focused on stroke, traumatic brain injury, spinal cord injury, Huntington’s disease, epilepsy, generic frailty, non-neurological populations, or other non-target conditions were excluded unless a target-disorder subgroup was separately extractable. Dementia studies were excluded when the sample was limited to vascular dementia, post-stroke dementia, vascular cognitive impairment, traumatic, infectious, metabolic, toxic, psychiatric, or otherwise non-neurodegenerative cognitive disorders, unless a neurodegenerative dementia subgroup was clearly reported separately.

Studies were excluded when they focused only on inpatient care, inpatient rehabilitation, acute treatment, institutional care, or routine clinical monitoring without a post-discharge, return-home, outpatient, home-based, community-based, caregiver-mediated, rehabilitation-continuity, palliative-continuity, or service-coordination component. Routine outpatient follow-up was not sufficient for inclusion unless it was explicitly linked to discharge, return home, home management, community re-entry, rehabilitation carry-over, caregiver support, treatment continuity, or escalation planning.

Pharmacological, surgical, biological, respiratory-device, assistive-device, nutritional, digital-device, or technology-efficacy studies were excluded when they evaluated the efficacy of a treatment or device alone and did not include a care-continuity, service-delivery, home-management, telemonitoring, caregiver-support, rehabilitation-aftercare, or palliative care pathway. Studies limited to long-term nursing-home residence, residential care, inpatient hospice, or institution-only end-of-life care were excluded when no home, outpatient, caregiver-mediated, or community-continuity pathway was described.

Non-original publications were excluded, including reviews, systematic reviews, scoping reviews, meta-analyses, umbrella reviews, editorials, commentaries, letters, opinion papers, protocols without results, conference abstracts, conference proceedings, theses, dissertations, and book chapters. Paediatric-only studies, non-human studies, non-English articles, inaccessible full texts, and reports lacking sufficient empirical information to determine population eligibility, transition relevance, care component, or outcome relevance were also excluded. Duplicate or overlapping reports were excluded when they did not add distinct data beyond a more complete or more relevant publication from the same study program.

### 2.4. Information Sources and Search Strategy

Electronic searches were conducted in PubMed/MEDLINE, Web of Science Core Collection, Embase, Scopus, and the Cochrane Library from database inception to 11 May 2026. The search strategy was developed iteratively through preliminary scoping of terminology used in neurological transition care, rehabilitation-continuity, home care, telehealth, and palliative care literature. Initial strategies were piloted before protocol registration to verify sensitivity and to identify disease-specific terminology. After protocol registration, all database searches were updated and verified on 11 May 2026, before the definitive evidence set and final synthesis were fixed.

Three broad disease-domain search strategies were used to reflect the structure of the evidence base identified during preliminary scoping. The first strategy targeted dementia/ADRD transition evidence, including hospital-to-home transition, discharge planning, caregiver-mediated continuity, medication management, home health, readmission, and return-home support. The second strategy targeted PD and MS rehabilitation-continuity evidence, including inpatient rehabilitation, multidisciplinary rehabilitation, telerehabilitation, mobile health (mHealth), post-discharge follow-up, carry-over, and return-home support. The third strategy targeted ALS home-based, respiratory, telehealth, and palliative-continuity evidence, including telemedicine, telemonitoring, home care, non-invasive ventilation, caregiver involvement, follow-up, continuity, and advance care planning.

Searches combined controlled vocabulary, where available, and free-text terms for disease population, continuity or transition concepts, and care setting. Boolean operators, truncation, phrase searching, field tags, and proximity operators were adapted to each database interface. Comparator terms were not used as mandatory search blocks because eligible evidence could include uncontrolled, qualitative, observational, implementation, feasibility, and service-evaluation studies. Outcome terms were used selectively and only when they did not materially reduce search sensitivity. The ALS abbreviation was not used as a standalone search term to reduce irrelevant retrieval.

All search results were exported with available bibliographic metadata, including title, authors, year, journal, abstract, digital object identifier, and database source. Records were deduplicated in stages using digital object identifiers, exact and normalized titles, author combinations, publication years, and journal information [38]. Database-specific yields were retained to support auditability and PRISMA-ScR reporting. Cochrane Library/Cochrane Reviews records were exported and reported as a single Cochrane source within the five-database PRISMA structure, not combined with records from other databases. Search reporting was informed by Peer Review of Electronic Search Strategies (PRESS) and the Preferred Reporting Items for Systematic Reviews and Meta-Analyses literature-search extension (PRISMA-S) principles [34,39]. The complete Boolean strategies, including database-specific field tags, controlled vocabulary where applicable, truncation, proximity syntax, limits, and exact records retrieved from each database, are reported in Supplementary Tables S1 and S2.

### 2.5. Selection of Sources of Evidence

Before formal screening, R.S.C. and A.C. completed a calibration exercise on a random sample of records to harmonize interpretation of the eligibility criteria and reduce inconsistent decisions. Particular attention was given to records in which the transition anchor was only implicit or incompletely reported, including studies describing caregiver support, rehabilitation carry-over, home healthcare, telehealth, telemonitoring, respiratory continuity, or palliative continuity without using explicit transitional-care terminology.

Titles and abstracts were screened independently by R.S.C. and A.C. Records were retained for full-text review when either reviewer judged them eligible or potentially eligible. This conservative approach was adopted because discharge timing, post-discharge setting, caregiver involvement, and continuity-of-care components were often insufficiently described in titles and abstracts. Clearly irrelevant records were excluded at this stage when they did not meet the target population, transition relevance, care-component, outcome, or publication-type criteria.

Full texts were then assessed independently by the same two reviewers (R.S.C. and A.C.). During full-text review, eligibility decisions were based on the complete report, including methods, intervention description, follow-up procedures, outcomes, tables, and Supplementary Information when available. Disagreements were resolved through discussion; unresolved cases were adjudicated by A.Q. Each excluded full-text report was assigned one primary exclusion reason, prioritizing the main reason for non-eligibility when more than one criterion was not met. The final exclusion categories were no post-discharge or transition component, wrong population or non-extractable target subgroup, wrong care model, setting or outcome, review/protocol/conference/non-original publication, and duplicate or overlapping report without additional eligible data.

Interrater agreement was calculated before consensus resolution. Cohen’s kappa was 0.71 for title/abstract screening and 0.75 for full-text eligibility assessment, indicating substantial agreement. The study-selection process, including records identified, duplicates removed, records screened, full texts assessed, full-text exclusions, and included studies, was summarized using a PRISMA-ScR flow diagram.

### 2.6. Data Charting

Data charting was conducted using a standardized form developed a priori and pilot-tested on included studies representing different disease groups, study designs, transition settings, and care models. The pilot phase was used to refine variable definitions, align coding across disease areas, and ensure that both explicitly discharge-anchored studies and broader continuity-of-care studies could be captured consistently. These refinements clarified extraction categories but did not change the review question or eligibility criteria.

One reviewer (A.C.) charted each included report using the standardized form. R.S.C. and A.Q. independently verified the transition anchor, dominant mechanism, evidence category, and overlap status, and audited extracted fields against the full text when classification or interpretation was uncertain. Disagreements, unclear classifications, or uncertain interpretations were resolved by discussion among R.S.C., A.C., and A.Q. Charting agreement was not summarized with a separate kappa statistic because charting involved multi-field extraction and verification rather than a binary eligibility decision; screening agreement was reported separately. When information was not reported or could not be inferred reliably from the article, it was coded as not reported rather than imputed.

Charted variables included bibliographic information, country, study design, recruitment source, sample size, disease group, diagnostic subgroup when available, participant characteristics, disease stage or severity, cognitive or functional status, transition origin, post-transition destination, timing of enrolment, follow-up duration, care model or intervention components, professionals involved, caregiver role, delivery mode, remote or technology-enabled components, comparator type, outcome domains, measurement instruments, main findings, implementation features, equity or access issues, and author-reported limitations.

Each study was also coded for its dominant transition mechanism and evidence category. Companion reports, secondary analyses, and reports from overlapping programs or cohorts were flagged during charting to avoid double-counting them as independent evidence while retaining distinct information on mechanisms, outcomes, subgroups, follow-up, or implementation. After charting, extracted data were checked for internal consistency across the main manuscript tables and supplementary evidence-mapping tables. The charting form and operational definitions are provided in the Supplementary methodological framework.

### 2.7. Evidence Classification and Synthesis

To preserve inferential distinctions and control overlap, each included report was allocated to one of four mutually exclusive categories using operational boundary rules. Core transition evidence required an explicit discharge or immediate post-discharge start point and evaluated a structured transition or follow-up model. Return-home/community re-entry evidence required a post-discharge or community-return trajectory but did not necessarily test a discrete transitional-care intervention. Adjacent continuity evidence addressed home-based, remote, rehabilitation, respiratory, nutritional, caregiver, outpatient, or palliative continuity when the discharge anchor was less explicit or the model represented ongoing specialist/community management. Within this category, ALS models spanning repeated respiratory, nutritional, technological, and palliative adjustments were additionally described as a continuous, progression- or needs-triggered continuity pathway; this was a descriptive sublabel, not a fifth evidence category. Companion/secondary reports arose from overlapping programs or cohorts and contributed distinct mechanistic, outcome, implementation, or subgroup information but were not interpreted as independent replication.

Classification was applied during charting and verified during synthesis by R.S.C., A.C., and A.Q., with disagreements resolved by discussion and, when required, adjudication by A.Q. Counts and proportions were summarized overall and by disease. Direct inferences about discharge models were based primarily on core transition evidence; return-home evidence informed post-discharge trajectories; adjacent evidence informed components, feasibility, and implementation boundaries; and companion reports enriched interpretation without increasing the count of independently replicated programs. Evidence categories therefore indicate transition proximity and reporting overlap, not methodological quality, sample size adequacy, or certainty of evidence.

Synthesis used a descriptive, tabular, and framework-based narrative approach. Study distributions, care models, outcomes, and evidence categories were summarized numerically. Narrative synthesis compared charted mechanisms across diseases, grouped them by transition proximity and care function, and distinguished cross-cutting mechanisms from disease-specific continuity needs. No statistical pooling was undertaken, and causal or definitive efficacy claims were avoided when comparators were absent or weak, samples were small, follow-up was limited, or outcomes were heterogeneous.

## 3. Results

### 3.1. Study Selection and Screening Yield

The database searches identified 24,417 records from five sources: PubMed (*n* = 1023), Web of Science (*n* = 862), Cochrane Library and Cochrane Reviews (*n* = 252), Embase (*n* = 1822) and Scopus (*n* = 20,458). No register records were identified. Before screening, 2349 duplicate records were removed. No records were excluded by automation tools, and no non-English records were removed before screening. The remaining 22,068 records were screened by title and abstract.

**Figure 1:**
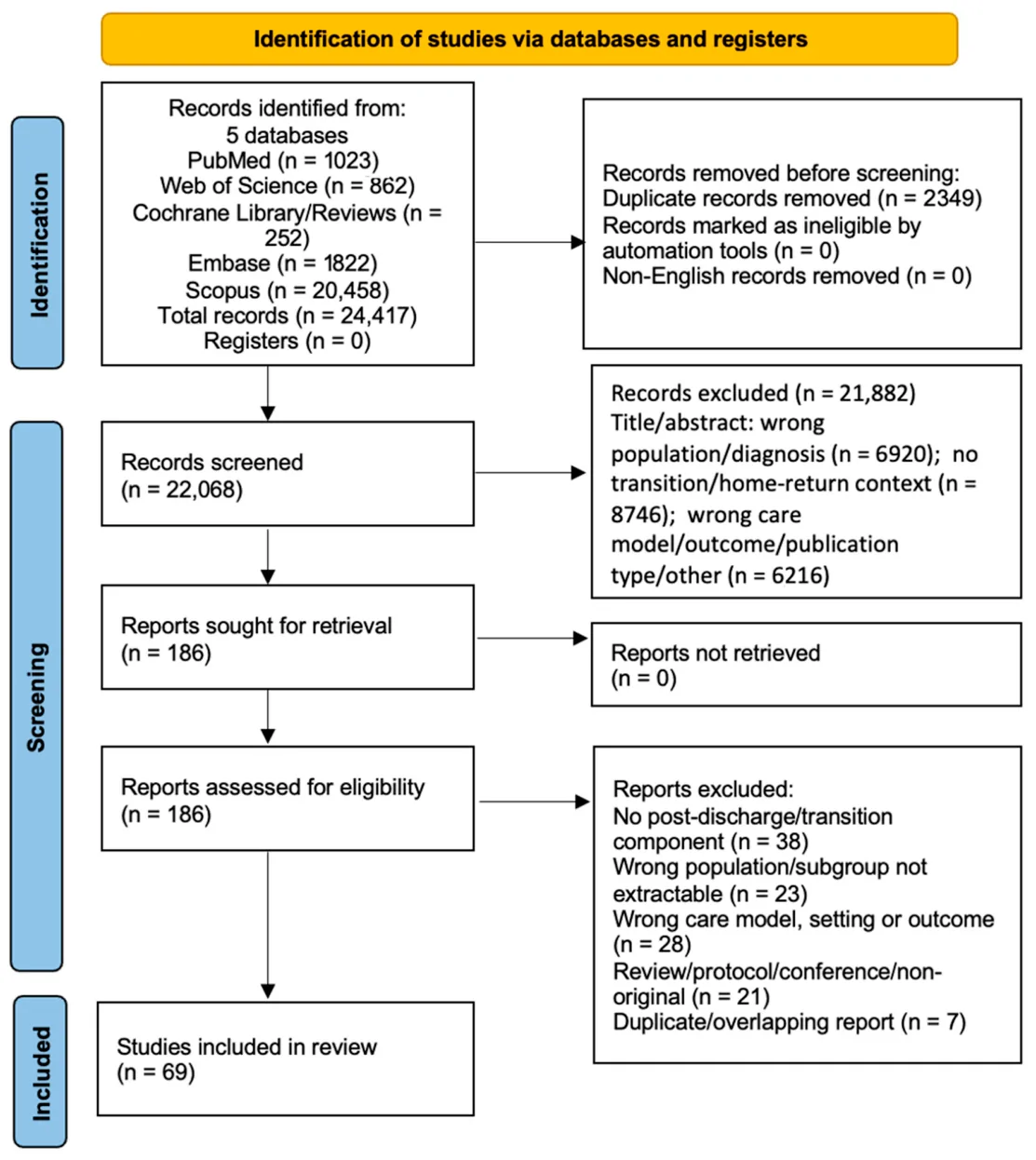
PRISMA-ScR flow diagram for study selection. The diagram reports database sources, duplicate removal, title and abstract screening, full-text assessment, reasons for exclusion, and final inclusion. Flow-diagram reporting follows PRISMA-ScR guidance [29].

At title and abstract screening, 21,882 records were excluded because they did not meet the population, transition-context, care-model, outcome or publication-type criteria. Full texts were sought for 186 reports, and all were retrieved. After full-text assessment, 117 reports were excluded. The main reasons for exclusion were absence of a post-discharge or transition component (*n* = 38), wrong population or non-extractable target subgroup (*n* = 23), wrong care model, setting or outcome (*n* = 28), review, protocol, conference or non-original publication (*n* = 21), and duplicate or overlapping report without additional eligible data (*n* = 7). The final evidence map included 69 reports. The study-selection process is shown in Figure 1.

### 3.2. Overall Evidence Landscape

The 69 included reports comprised 28 dementia/ADRD reports, 10 PD reports, 9 MS reports, and 22 ALS reports. Designs included randomized and non-randomized trials, observational cohorts, qualitative and mixed-methods studies, implementation studies, feasibility studies, and secondary analyses. Companion or secondary publications from overlapping programs were retained only when they contributed distinct mechanistic, outcome, subgroup, follow-up, or implementation information; they were not treated as independent program replication. Disease-specific characteristics are summarized in the disease-specific evidence tables. The study-level evidence classification is provided in Supplementary Table S3.

Overall, 18 reports (26.1%) were classified as core transition evidence, 14 (20.3%) as return-home/community re-entry evidence, 16 (23.2%) as adjacent continuity evidence, and 21 (30.4%) as companion/secondary reports. Dementia/ADRD contributed 14 core, 3 return-home, and 11 companion reports; PD contributed 3 core and 7 return-home reports; MS contributed 1 core, 4 return-home, and 4 companion reports; and ALS contributed 16 adjacent and 6 companion reports. No ALS report was classified as independent core transition or return-home/community re-entry evidence under the prespecified operational definitions, reflecting the predominance of ongoing home-based respiratory, specialist, telehealth, and palliative continuity rather than a discrete discharge-anchored pathway.

The classification materially guided the synthesis. Core reports supported the most direct statements about discharge-anchored models; return-home reports characterized functional, participation, equity, and service trajectories; adjacent reports informed candidate components and implementation requirements; and companion reports were used for additional outcomes or mechanisms without being treated as separate program replication.

### 3.3. Dementia/ADRD: Hospital-to-Home, Caregiver-Mediated, Medication, Home-Health and Palliative Transitions

Dementia/ADRD supplied 14 of the 18 core transition reports and was therefore the most directly discharge-anchored component of the map. The 28 reports addressed discharge-plan adequacy, special Alzheimer acute care unit follow-up, system failures in discharge planning, family-centred function-focused care, discharge documentation, medication-management support, home-health trajectories, skilled nursing facility (SNF)-to-home transitions, caregiver preparedness, palliative care transition, and equity in home-health agency quality [40,41,42,43,44,45,46,47,48,49,50,51,52,53,54,55,56,57,58,59,60,61,62,63,64,65,66,67]. Study-level details are presented in Table 1.

Earlier work showed that inadequate aftercare planning and insufficient caregiver support were associated with early readmission after neuropsychiatric hospitalization for dementia [40]. A post-discharge follow-up intervention after discharge from a special Alzheimer acute care unit reported feasibility of structured caregiver or resource-person contact, although reductions in early emergency room rehospitalization were not statistically significant [41]. Qualitative and documentation audit studies from Australia identified recurrent failures in discharge communication, medication orders, Dose administration aids, community-service linkage, general practitioner communication and carer information [42,44]. Medication-focused work further showed that caregivers often received insufficient information about medication changes, indications, cognitive adverse effects and medication supply after discharge [45,48].

Intervention evidence in dementia was largely caregiver mediated. The Family-Centred Function-focused Care (Fam-FFC) program used family engagement, function-focused goals and post-acute telephone support to target functional recovery, behavioural symptoms and caregiver preparedness [43,57,58,62]. Connect-Home ADRD adapted SNF transitional care to the needs of people with dementia and caregivers, combining pre-discharge planning, rapid home-care linkage and specialist post-discharge calls [51,52,64]. Qualitative studies clarified why these mechanisms matter: Caregivers frequently became the constant source of information, the safety monitor, the advocate, and the service navigator during the hospital-to-home or SNF-to-home transition [51,55,60,66,67].

Home-health and post-acute care evidence contributed a second major dementia pathway. Medicare Outcome and Assessment Information Set (OASIS) studies linked dementia severity, mobility, self-care dependence, cognition, medication assistance and caregiver support with potentially preventable readmissions, therapy completion, home-health utilization and successful discharge to community [46,47,49,50,61]. Claims-based studies also showed that post-acute pathways and home-health agency quality were shaped by race, ethnicity, Medicare–Medicaid dual eligibility and coronavirus disease 2019 (COVID-19)-era service shifts [56,65]. Palliative-transition studies in advanced dementia emphasized caregiver stressors, barriers to palliative-service access, goals-of-care continuity, post-discharge calls, grief support and the need for clearer future planning after hospital or emergency department contact [54,63].

### 3.4. Parkinson’s Disease and Multiple Sclerosis: Rehabilitation Continuity, Return-Home Support and Post-Discharge Functional Follow-Up

The PD and MS evidence included studies of rehabilitation continuity, return-home support, and post-discharge functional follow-up [21,22,24,25,68,69,70,71,72,73,74,75,76,77,78,79,80,81,82]. PD comprised three core and seven return-home reports, whereas MS comprised one core, four return-home, and four companion reports. Accordingly, this domain primarily informed rehabilitation continuity, functional carry-over, and return-home support rather than classic multicomponent discharge-planning models. PD studies addressed intensive inpatient rehabilitation, multimodal complex treatment, telerehabilitation, medication continuity, inpatient mobility, advanced-disease return home, and mHealth-supported self-management. MS studies addressed rehabilitation carry-over, home-based management, internet-based exercise aftercare, telecoaching, fatigue self-management, and goal-stratified multidisciplinary rehabilitation. Study-level details are presented in Table 2.

MS studies consistently framed the discharge period as a challenge of translating rehabilitation gains into daily life. Early longitudinal evidence showed that disability, handicap and emotional well-being benefits after inpatient rehabilitation could persist for months despite neurological progression [21]. Home-based multidisciplinary management improved selected health-related quality-of-life domains without increasing costs [22]. Later trials and follow-up studies from the Danish MS Hospitals program showed that inpatient multidisciplinary rehabilitation produced short- and longer-term quality-of-life benefits, but the magnitude and persistence of gains varied by patient focus area, baseline needs and rehabilitation goals [70,73,79]. Digital aftercare and telecoaching studies suggested that fatigue, physical activity, self-management and neuropsychological goal carry-over may require structured support after discharge rather than passive instruction alone [24,25,77].

PD studies mapped a related but disease-specific transition problem. Intensive inpatient rehabilitation and multimodal complex treatment were associated with short-term motor, functional or quality-of-life gains, with partial persistence after discharge in several cohorts [68,69,71,75,82]. The strongest return-home signal came from advanced PD, where a combined 6-week inpatient multidisciplinary program followed by 2 years of outpatient support stabilized activities of daily living and delayed definite nursing-home admission in many treated patients [78]. Other work identified medication continuity and early mobilization as transition-relevant safety domains: Unintended medication changes occurred after discharge in people with PD, and inpatient mobility was associated with shorter length of stay and higher probability of home-to-home discharge [74,80]. Post-rehabilitation mHealth support extended inpatient rehabilitation into daily physical-activity and nutrition self-management, with improved walking capacity, activity and quality of life at 6 months [81].

### 3.5. Amyotrophic Lateral Sclerosis: Home-Based, Telehealth, Respiratory and Palliative Care Continuity

All 22 ALS reports were classified as adjacent continuity evidence (*n* = 16; 72.7%) or companion/secondary reports (*n* = 6; 27.3%). None met the operational definition for independent core transition or return-home/community re-entry evidence. The ALS literature focused on home-based multidisciplinary continuity, respiratory support, telehealth, telemonitoring, nutritional and equipment management, caregiver support, palliative care continuity, advance care planning, and end-of-life pathways [83,84,85,86,87,88,89,90,91,92,93,94,95,96,97,98,99,100,101,102,103,104]. Because these functions are repeatedly activated by progression or changing needs rather than by one discharge event, they were described within adjacent continuity as a continuous, progression- or needs-triggered continuity pathway. This subdescriptor did not change report allocation or counts. These findings therefore inform continuity components and implementation requirements more directly than the effectiveness of a discrete discharge model. Study-level details are presented in Table 3.

**Table 3:** **Home-based, telehealth, respiratory, multidisciplinary and palliative care continuity evidence in amyotrophic lateral sclerosis.** This table summarizes studies in which home-based multidisciplinary care, telemedicine, telemonitoring, non-invasive ventilation support, respiratory or nutritional continuity, case management, caregiver support, specialist palliative care, or end-of-life care pathways represented the main continuity-of-care component.

Author, Year [Ref.]/Location/Country	Aim and Study Design	ALS Population Characteristics	Care Setting and Transition Anchor	Care Model or Continuity Component	Technology, Respiratory, Nutritional or Palliative Component	Outcome Measures	Main Findings	Key Transition Mechanism and Contribution to the Evidence Map
**Study:** Pinto et al., 2010 [83] **Location/Country:** Portugal	**Aim:** Test whether modem-based NIV telemonitoring reduces healthcare utilization **Study design:** Prospective, single-blinded controlled trial	**ALS population:** N = 40 consecutive ventilated ALS patients assigned by residence; control *n* = 20, telemonitoring *n* = 20, with 1 intervention dropout analysed as *n* = 19. Age 18–75 y; no respiratory insufficiency at diagnosis; FVC ≥ 75% at baseline.	**Care setting/transition anchor:** Home NIV follow-up after NIV adaptation; office-based monitoring versus weekly remote modem review plus 3-month office visits.	**Care model/continuity component:** Control: Office visits at NIV onset, 2–3 weeks, then every 3 months. Intervention: Modem-connected ventilator review weekly and when discomfort occurred; helpline available.	**Technology/respiratory/nutritional/palliative component:** GoodKnight 425ST bi-level NIV with internal modem; remote transmission of usage, pressures, volumes, respiratory rate, leaks, apnoeas and setting changes.	**Outcome measures:** Office visits, emergency room visits, and admissions; NIV compliance and setting changes; ALSFRS and respiratory subscores; survival with NIV.	**Main findings:** Office visits, emergency room visits and hospital admissions were significantly lower in the telemonitoring group (*p* < 0.0001). Compliance did not differ; setting changes were more frequent during initial adaptation but lower over total NIV use; survival trend favoured telemonitoring (*p* = 0.13).	Mechanism/contribution: Respiratory telemonitoring mechanism: Remote NIV data review can reduce healthcare utilization while supporting setting optimization. Evidence stratum: Adjacent continuity evidence; ALS NIV telemonitoring.
**Study:** De Almeida et al., 2010 [84] **Location/Country:** Portugal	**Aim:** Describe and test a wireless device for real-time telemedical assistance in home-ventilated ALS **Study design:** Technical feasibility/implementation study	**ALS population:** Home-ventilated ALS patients followed in a reference ALS clinic; exact patient N NR. Clinic context included about 170 ALS patients, about 100 using GoodKnight 425ST home ventilation.	**Care setting/transition anchor:** Home ventilation monitoring in a multidisciplinary ALS clinic; designed to support remote NIV follow-up in the home environment.	**Care model/continuity component:** Wireless instrument interfaced with the ventilator to record and transmit ventilatory data; physician could review data and adjust settings remotely or online.	**Technology/respiratory/nutritional/palliative component:** Bi-directional TCP/IP modem system; stored ventilator parameters outside hospital; alerts based on IPAP, EPAP, I:E ratio, backup rate, trigger sensitivity and rise time. Testing accepted errors < 5%.	**Outcome measures:** Performance, reliability, user-friendliness, transmission robustness, safety/acceptance and ventilator-parameter monitoring.	**Main findings:** The device and software were user-friendly, robust for data transmission and practical for patients and therapists. Main limitations were need for a fixed telephone line and slow data extraction; the report supported controlled efficacy testing.	Mechanism/contribution: Home ventilation monitoring mechanism, which establishes a technical platform for remote ventilator pattern recognition, parameter review and early troubleshooting. Evidence stratum: Adjacent continuity evidence; technical feasibility report.
**Study:** Vitacca et al., 2010 [85] **Location/Country:** Italy	**Aim:** Evaluate feasibility, team roles, intervention areas and satisfaction with TAIC after discharge **Study design:** Pilot telemedicine-assisted integrated-care trial	**ALS population:** N = 40 advanced ALS patients after hospital discharge; mean age 63 y; 60% men; bulbar involvement 62%; spontaneous breathing 23%, NIV 48%, invasive ventilation 30%; gastrostomy 50%; 40 caregivers.	**Care setting/transition anchor:** Home monitoring immediately after hospital discharge from a rehabilitation/ALS unit.	**Care model/continuity component:** Nurse-tutor-led TAIC with scheduled weekly contacts, unscheduled calls, multidisciplinary backup and web-based electronic records.	**Technology/respiratory/nutritional/palliative component:** Portable pulse oximeter for daily contacts; respiratory physician, neurologist, psychologist and respiratory therapist available; oximetry transmission in selected cases.	**Outcome measures:** Call volume/type, symptoms, oximetry, admissions, emergency visits, prescriptions, satisfaction and confidence in disease handling.	**Main findings:** Mean follow-up 8.6 months. There were 1907 scheduled calls and 317 unscheduled calls; 84% of unscheduled calls were managed by the nurse-tutor. TAIC recommended 4/12 emergency admissions and 77% of other hospitalizations. Satisfaction with nurse assistance was 79%; confidence improved in 71%.	Mechanism/contribution: Nurse-tutor TAIC mechanism: Scheduled and on-demand monitoring after discharge provides triage, oximetry-supported respiratory decisions and multidisciplinary escalation. Evidence stratum: Adjacent continuity evidence; nurse-centred ALS home follow-up.
**Study:** Vitacca et al., 2012 [86] **Location/Country:** Italy	**Aim:** Describe long-term TAIC activity, nurse workload, costs and timing of tele-assistance **Study design:** Prospective service-utilization study	**ALS population:** N = 73 ALS patients after hospital discharge, ALS-FRS < 40; mean age 60.8 y; 60.3% male; 60.3% bulbar; spontaneous breathing 50.6%, NIV 24.7%, invasive ventilation 24.7%; gastrostomy 34.2%; ALS-FRS 28+/−9.7.	**Care setting/transition anchor:** Post-discharge home follow-up from a specialized rehabilitation hospital over 4 years.	**Care model/continuity component:** Long-term nurse-tutor telephone TAIC using a standardized ALS-card, scheduled and symptom-triggered calls, and medical second opinions.	**Technology/respiratory/nutritional/palliative component:** Telephone triage, pulse-oximeter support, ALS-card risk scoring, nurse-tutor coordination and cost analysis.	**Outcome measures:** Number of calls/patient/month, nurse time, feasibility/usefulness of ALS-card, survival, costs and staffing capacity.	**Main findings:** TAIC generated 5073 calls; 78.6% were scheduled and 14.2% required direct doctor involvement. Steady-state activity was about 5 calls/patient/month; a nurse could follow about 25 patients/month. Mean cost was EUR105.2+/−41.3 per patient/month; TAIC was proposed at about two-thirds of disease course.	Mechanism/contribution: Scalable tele-assistance mechanism quantifies the staffing, call burden and cost profile needed to maintain ALS follow-up at home. Evidence stratum: Companion report;
**Study:** Creemers et al., 2014 [87] **Location/Country:** Netherlands	**Aim:** Test whether case management added to multidisciplinary ALS care improves patient QoL, caregiver strain and perceived care quality **Study design:** Multicentre cluster RCT	**ALS population:** N = 132 ALS patients and 126 caregivers enrolled; intervention *n* = 71 patients/66 caregivers, control *n* = 61/60. Baseline ALSFRS-R mean 32 in both groups; limb onset 73% vs. 79%; NIV/IV 17% vs. 18%.	**Care setting/transition anchor:** Community and secondary multidisciplinary ALS-team care over 12 months.	**Care model/continuity component:** Case management plus usual multidisciplinary care versus usual care alone; case managers made baseline and 3-monthly home visits with phone/email contact between visits.	**Technology/respiratory/nutritional/palliative component:** Independent case manager outside but linked to ALS team; home visits 60–180 min; symptom checklist and navigation/support functions.	**Outcome measures:** ALSAQ-40 Emotional Functioning, Caregiver Strain Index, quality-of-care ratings and ALSFRS-R over 0, 4, 8 and 12 months.	**Main findings:** No significant benefit was found for ALSAQ-40 emotional functioning, CSI or quality-of-care ratings. CSI increased over time in both groups (*p* < 0.0001), while perceived care quality remained high.	Mechanism/contribution: Case-management mechanism tests whether formal navigation support adds value when multidisciplinary ALS care is already established; neutral comparative evidence. Evidence stratum: Adjacent continuity evidence; comparative case-management RCT.
**Study:** Bakker et al., 2015 [88] **Location/Country:** Netherlands	**Aim:** Explore perceived need and added value of case management in multidisciplinary ALS care **Study design:** Qualitative study linked to the Dutch case-management program	**ALS population:** Interviews with N = 10 patients, 10 spousal caregivers and 10 professionals; focus group *n* = 20. Patients: Mean age 61 y; disease duration mean 28.5 months; slow progression *n* = 4, medium *n* = 4, rapid *n* = 2.	**Care setting/transition anchor:** Multidisciplinary ALS care and community follow-up in the context of a prior RCT.	**Care model/continuity component:** Case management as a supplementary support model; explored receptiveness and valued features rather than testing effectiveness.	**Technology/respiratory/nutritional/palliative component:** Home interviews, focus group, stakeholder perspectives on access, house calls, time, proactive support and emotional support.	**Outcome measures:** Qualitative themes: Need/receptiveness and appreciated aspects; factors included ALS-team functioning, progression rate, personal coping and social support.	**Main findings:** Participants valued house calls, ample consultation time, proactive care and emotional support. Added value was greatest when usual care was suboptimal, disease progression rapid, coping passive, or social networks limited.	Mechanism/contribution: Relational case-management mechanism identifies the specific coordination ingredients that may matter even when RCT-level effectiveness is neutral. Evidence stratum: Companion report; qualitative perspective on Dutch case-management model.
**Study:** Cordesse et al., 2015 [89] **Location/Country:** France	**Aim:** Evaluate whether a coordinated community ALS network changed hospitalization, function and survival **Study design:** Before/after cohort study	**ALS population:** N = 2452 probable or definite ALS patients followed in 2000–2012 in Ile de France; 91.5% regional coverage in 2012. Incident pre-network and network periods compared.	**Care setting/transition anchor:** Before and after creation of a community care network alongside the Salpêtrière ALS centre.	**Care model/continuity component:** Care pathway coordinators linking specialist centre, community professionals and home-based services; same medical standards before/after network creation.	**Technology/respiratory/nutritional/palliative component:** Care coordination; NIV use increased over time; respiratory/nutritional intervention details otherwise not fully separable.	**Outcome measures:** Hospitalizations, emergency admissions, ALSFRS-R functional deterioration slope, survival and Cox models.	**Main findings:** Hospitalization variables decreased after network creation. ALSFRS-R slope improved from 1.03+/−1.57 to 0.79+/−0.80 points/month (*p* = 0.002). Median survival was 38.8 versus 25.6 months; coordinated care was associated with lower mortality risk.	Mechanism/contribution: Community-network mechanism: Coordination across specialist and community services may reduce admissions and preserve continuity at home. Evidence stratum: Adjacent continuity evidence; large coordinated-care cohort.
**Study:** Hobson et al., 2019 [90] **Location/Country:** United Kingdom	**Aim:** Pilot feasibility of Telehealth in Motor Neuron Disease (TiM) plus usual care versus usual care **Study design:** 18-month mixed-methods randomized feasibility study	**ALS population:** N = 40 MND patients and 37 carers recruited from a UK specialist MND centre; telehealth *n* = 20, control *n* = 20. Age range 30–78 y; 33% had NIV or gastrostomy (King stage 4).	**Care setting/transition anchor:** Home/community specialist MND care, with usual clinic invitations every 2–6 months.	**Care model/continuity component:** TiM enabled weekly home symptom/function reporting to specialist nurses, who could contact patients or expedite care.	**Technology/respiratory/nutritional/palliative component:** TiM app, home reporting of function, symptoms, mood, carer strain, weight and balance; nurse monitoring and qualitative process evaluation.	**Outcome measures:** Recruitment/retention/data completion, QoL, ALSFRS-R, anxiety/depression, health resource use and acceptability.	**Main findings:** Recruitment target achieved; 80% of patients and 82% of carers completed 6-month outcomes. Retention was good, and participation burden was low. TiM was acceptable, but definitive RCT evaluation was judged challenging for this complex intervention.	Mechanism/contribution: Telehealth feasibility mechanism: Low-burden home reporting can include severely disabled MND patients and support specialist access. Evidence stratum: Adjacent continuity evidence; feasibility trial design.
**Study:** Pulley et al., 2019 [91] **Location/Country:** USA	**Aim:** Assess whether store-and-forward telemedicine can deliver multidisciplinary ALS care **Study design:** Feasibility and satisfaction study	**ALS population:** N = 18 ALS/PLS/PMA patients completed 27 telemedicine encounters; mean age 64 y; median ALSFRS-R 25.5; median distance from clinic 49 miles; disease duration from onset median 27 months.	**Care setting/transition anchor:** Home-based alternative to in-person multidisciplinary ALS clinic for patients previously seen in the clinic.	**Care model/continuity component:** Trained telemedicine nurse recorded home assessment; team reviewed discipline-specific videos and generated recommendations; neurologist discussed plan by video/phone.	**Technology/respiratory/nutritional/palliative component:** Two iPad recordings, secure server, HIPAA-compliant videoconference, asynchronous multidisciplinary review.	**Outcome measures:** Patient and provider satisfaction; feasibility of obtaining assessment information and developing a care plan.	**Main findings:** Patient satisfaction was excellent for all 27 visits. Providers generally agreed they could obtain information, create care plans and care for patients, although some concerns remained about time, equipment and lack of physical contact.	Mechanism/contribution: Store-and-forward multidisciplinary mechanism separates home assessment, team review and care-plan feedback to reduce travel while preserving multidisciplinary input. Evidence stratum: Adjacent continuity evidence.
**Study:** Helleman et al., 2020 [92] **Location/Country:** Netherlands	**Aim:** Evaluate ALS home-monitoring and coaching as part of specialized care **Study design:** Prospective single-centre cohort with surveys/interviews	**ALS population:** N = 50 ALS/PMA patients who used telehealth: 18 prevalent and 32 incident users; 64% male; 76% ALS; mean age at diagnosis 61.4 y; ALSFRS-R at monitoring start 38.3+/−8.1; median follow-up 10.8 months.	**Care setting/transition anchor:** Home monitoring integrated into specialist ALS clinic care.	**Care model/continuity component:** ALS app self-monitoring plus nurse-practitioner feedback and referral to the multidisciplinary team.	**Technology/respiratory/nutritional/palliative component:** ALS app for daily well-being, weekly weight, monthly ALSFRS-R; message function; alerts for weight loss, low well-being or ALSFRS-R decline.	**Outcome measures:** Adoption, dropout, adherence, messages/alerts, patient and professional user experiences.	**Main findings:** Incident-patient adoption was 80% and dropout 4%. Good adherence was 49% for well-being, 83% for body weight and 87% for functional assessment. Patients sent 2003 messages; 899 alerts were generated; user experiences were mainly positive.	Mechanism/contribution: App-based monitoring mechanism: Regular self-monitoring, alerts and nurse feedback maintain continuity between clinic visits. Evidence stratum: Adjacent continuity evidence;
**Study:** Capozzo et al., 2020 [93] **Location/Country:** Southern Italy	**Aim:** Describe feasibility and acceptability of ALS telemedicine during COVID-19 restrictions **Study design:** Observational telemedicine survey	**ALS population:** N = 32 selected ALS/PLS/ALS-FTD patients; 31 interviews completed: 8 directly with patients and 23 with caregivers. Mean age 65.65 y; ALS *n* = 25, ALS-FTD *n* = 4, PLS *n* = 3; mean ALSFRS-R 32.19.	**Care setting/transition anchor:** COVID-era substitution for multidisciplinary centre visits in southern Italy.	**Care model/continuity component:** Structured telephone/cell-phone telemedicine assessment by neurologist; video was offered but refused by all patients.	**Technology/respiratory/nutritional/palliative component:** Telemedicine questionnaire covering motor, cognitive/behavioural, sleep, respiratory, nutrition, swallowing, rehabilitation, therapy and COVID-19 symptoms.	**Outcome measures:** Clinical changes, follow-up needs, therapy renewal, urgent hospitalization, satisfaction and willingness to continue telemedicine.	**Main findings:** Follow-up visit was ordered for 17/31 patients. One patient required urgent hospitalization and tracheostomy. Physiotherapy interruption was reported by 13/31. Satisfaction survey response was 65%; 70% were very satisfied, 30% fairly satisfied, and 90% wanted future telehealth follow-up.	Mechanism/contribution: COVID-era telecare mechanism: Structured remote triage preserved ALS follow-up, therapy renewal and urgent escalation during service disruption. Evidence stratum: Adjacent continuity evidence.
**Study:** Munan et al., 2020 [94] **Location/Country:** Canada	**Aim:** Describe ALS transitions experienced by patients and caregivers **Study design:** Qualitative descriptive study	**ALS population:** N = 14 PwALS and 15 caregivers from a multidisciplinary ALS clinic; PwALS mean age 69.7+/−9.3 y; caregivers mean age 62.5+/−11.7 y; mean time since diagnosis 2.9+/−2.7 y.	**Care setting/transition anchor:** Transitions to home mechanical ventilation, PEG, assistive communication technology, dependency and future planning.	**Care model/continuity component:** No intervention; semi-structured home interviews with PwALS-caregiver dyads, analysed thematically.	**Technology/respiratory/nutritional/palliative component:** HMV/BPAP, PEG, mobility equipment, assistive communication technology, ACP and social support themes.	**Outcome measures:** Qualitative themes: Social relationships, caregiver role, medical devices, future decision-making and communication transitions.	**Main findings:** Participants described loneliness, caregiver role change and total responsibility. Early information and adoption of HMV, PEG and communication aids were linked to better QOL; participants wanted earlier ACP information.	Mechanism/contribution: Patient-caregiver transition mechanism maps the lived transitions that continuity models must anticipate, especially devices, dependency, communication loss and ACP. Evidence stratum: Adjacent continuity evidence; qualitative transition evidence.
**Study:** De Marchi et al., 2021 [95] **Location/Country:** Italy	**Aim:** Evaluate multidisciplinary ALS telehealth during COVID-19 and patient satisfaction **Study design:** Observational pilot study	**ALS population:** N = 19 ALS patients enrolled; age at onset 51.48+/−12.18 y; female 63%; spinal onset 79%; median disease duration 10 months; ALSFRS-R 32.05+/−9.22 at televisit onset; NIV 5/19; PEG 1/19.	**Care setting/transition anchor:** Telemedicine substitution for scheduled multidisciplinary ALS visits during March–May 2020.	**Care model/continuity component:** Video televisits by neurologist, dietician, psychologist and physiotherapist using the IoMT Connected Care platform.	**Technology/respiratory/nutritional/palliative component:** Telemedicine platform; neurological, nutritional, psychological and physiotherapy monitoring; NIV/pneumology referral and device prescriptions.	**Outcome measures:** Medication changes, ALSFRS-R, BMI/caloric intake, HADS, ALSAQ-40, Barthel/Borg/VAS, devices and satisfaction.	**Main findings:** Four providers completed 310 video televisits. Medication was changed in 11/19; 2/19 needed urgent pneumology and started NIV; 9/16 required device prescription. ALSFRS-R monthly decline was 0.88 before televisit and 0.49 during televisit; BMI/caloric intake remained stable.	Mechanism/contribution: Multidisciplinary televisit mechanism: Remote neurology, nutrition, psychology and physiotherapy can triage symptoms, NIV needs and devices during disrupted in-person care. Evidence stratum: Adjacent continuity evidence.
**Study:** Dontje et al., 2022 [96] **Location/Country:** Netherlands	**Aim:** Evaluate nationwide implementation of ALS home monitoring and coaching **Study design:** Implementation study using participatory action research and implementation frameworks	**ALS population:** ALS H&C implemented in 10 multidisciplinary rehabilitation settings; 86 patients started pilot, 71 completed evaluation; 76/148 healthcare providers responded. Patient median age 61 y; median ALSFRS-R 37.	**Care setting/transition anchor:** Home monitoring in multidisciplinary ALS rehabilitation teams after scale-up from the Utrecht ALS clinic.	**Care model/continuity component:** E-health monitoring of well-being, weight and functional status, with healthcare coach feedback and team integration.	**Technology/respiratory/nutritional/palliative component:** ALS H&C app/web portal; implementation action plans, healthcare protocol, app messages and monitoring frequency.	**Outcome measures:** Acceptability, appropriateness, feasibility, fidelity, sustainability, technology acceptance and usability.	**Main findings:** Patient adoption during pilot was 67.2%. Patients rated care concept 8/10 and app 7/10. Providers rated care concept 7/10 and SUS 58.8+/−11.3. Seven of nine teams completing implementation continued ALS H&C; two stopped, mainly due to platform-integration issues.	Mechanism/contribution: Implementation mechanism identifies requirements for scaling ALS home monitoring beyond an expert centre, including usability, fidelity and EHR integration. Evidence stratum: Companion report;
**Study:** Reginault et al., 2023 [97] **Location/Country:** France	**Aim:** Assess at-home NIV initiation with telemonitoring compared with in-hospital initiation **Study design:** Retrospective cohort study	**ALS population:** N = 265 ALS patients with NIV indication; at-home initiation *n* = 184, in-hospital *n* = 81. Mean age 68+/−11 y; 63% male; bulbar symptoms 53%; mean time from symptom onset to NIV initiation 29+/−25 months.	**Care setting/transition anchor:** NIV initiation at home versus hospital in a French ALS centre.	**Care model/continuity component:** 30-day at-home NIV initiation protocol with specialized physiotherapist visits, teleconsultations and telemonitoring.	**Technology/respiratory/nutritional/palliative component:** Lumis VPAP/Airview telemonitoring; home visits days 1, 3 and 30; telemonitoring/phone on days 2 and 10; nocturnal oximetry on day 30 in adherent home patients.	**Outcome measures:** NIV adherence >4 h/day at day 30; nocturnal hypoxaemia correction; prescription-to-initiation delay; leaks/AHI/respiratory rate.	**Main findings:** At 30 days, adherence > 4 h/day was 66% overall, 70% at home and 52% in hospital (*p* = 0.018). At-home initiation was faster (8.7+/−6.5 vs. 29+/−31.1 days, *p* < 0.001). In adherent home patients, nocturnal hypoxaemia was corrected in 79%.	Mechanism/contribution: Home NIV initiation mechanism: Structured physiotherapist-led home initiation plus telemonitoring can accelerate NIV access and monitor early adherence. Evidence stratum: Adjacent continuity evidence; home respiratory continuity.
**Study:** Fidelix et al., 2023 [98] **Location/Country:** Brazil	**Aim:** Evaluate feasibility and acceptability of telehealth for multidisciplinary ALS care in a Brazilian reference centre **Study design:** Retrospective cohort study	**ALS population:** N = 46 ALS patients; 32 male/14 female; mean age 55.8 y; mean distance 115 km. Spinal onset 78%; King stage 4b 59%; ALSFRS-R < 39 in 78%; NIV 54%, gastrostomy 26%, tracheostomy 11%.	**Care setting/transition anchor:** Home-based teleconsultations for patients with at least one previous in-person ALS evaluation.	**Care model/continuity component:** Multidisciplinary Teleconsulta platform with neurologist, motor/respiratory physiotherapists, speech/swallow therapist, dietician and psychologist.	**Technology/respiratory/nutritional/palliative component:** Teleconsulta or Google Meet; phone calls when internet/platform access limited; caregiver participation when needed.	**Outcome measures:** ALSFRS-R, King stage, symptoms, medication, equipment, exams, satisfaction and teleconsultation topics.	**Main findings:** Telehealth was viable and well accepted. The most frequent topics were respiratory adjustments (58%), medication adjustments (39%) and gastrostomy (28%). Satisfaction questionnaires were completed by 36 patients and 33 caregivers.	Mechanism/contribution: Telehealth access mechanism supports multidisciplinary ALS follow-up for advanced or geographically distant patients, especially respiratory and medication management. Evidence stratum: Adjacent continuity evidence.
**Study:** Bublitz et al., 2024 [99] **Location/Country:** Germany	**Aim:** Evaluate feasibility and satisfaction of a specialized homecare ALS neuropalliative model **Study design:** Longitudinal cohort study	**ALS population:** N = 94 patients in homecare project; 88 patients and 74 caregivers enrolled. Mean age 67.8+/−11.2 y; 52% male; limb onset 73.9%, bulbar 22.7%; initial ALSFRS-R 27.9+/−7.5; NIV 45.5%, gastrostomy 36.4%.	**Care setting/transition anchor:** Home-based specialized ALS care for patients unable to attend outpatient care in the Munich region.	**Care model/continuity component:** Regular multiprofessional home visits by neurologists/palliative physicians, nurse, social worker and chaplain.	**Technology/respiratory/nutritional/palliative component:** Neuropalliative care, home blood-gas checks in 39 cases, NIV coordination, 24-h nursing organization, hospice/palliative linkage and caregiver support.	**Outcome measures:** Home visits, care duration, place of death, satisfaction, caregiver burden, SAHD and end-of-life care quality.	**Main findings:** Total home visits = 1128; mean care duration 282 days overall and 221 days in deceased patients. Among 49 deceased patients, 61% died at home. Patient satisfaction was high (ICECAP-SCM 23.7/28; CollaboRATE 10.6/12); caregiver end-of-life care rating was high (CEQUEL 24.9/26).	Mechanism/contribution: Home neuropalliative mechanism: Multiprofessional home visits bridge immobility-related access barriers and allow many patients to remain at home through end of life. Evidence stratum: Adjacent continuity evidence.
**Study:** Kim et al., 2025 [100] **Location/Country:** South Korea	**Aim:** Describe video televisits in home-based ALS medical care **Study design:** Retrospective cross-sectional study	**ALS population:** N = 69 of 85 registered pALS received video televisits. Median age 66 y; 53.6% female; 71% non-ambulatory, 27.5% assisted ambulatory; median ALSFRS-R 26; 82.6% nutritional support; 78.3% home ventilator.	**Care setting/transition anchor:** Home-based medical care team follow-up at a tertiary hospital during temporary telemedicine permission.	**Care model/continuity component:** Video televisits within HBMC clinic; often conducted with care partners and combined with home visits for patients living in Seoul.	**Technology/respiratory/nutritional/palliative component:** Video telemedicine; nutritional support, gastrostomy/NG tube, NIPPV, tracheostomy invasive ventilation, equipment management and ACP.	**Outcome measures:** Televisit topics, equipment needs, acute issues, ACP discussions and frequency of televisits.	**Main findings:** Disease-related symptoms were addressed in 100% of patients; equipment management in 92.8%; acute health issues in 52.2%; ACP/goal-of-care discussions in 14.5%. During follow-up, ventilation support increased to 88.4% and tracheostomy to 44.9%.	Mechanism/contribution: Home-based televisit mechanism uses video visits to manage symptoms, equipment, acute issues and ACP for advanced, mobility-limited pALS. Evidence stratum: Adjacent continuity evidence.
**Study:** Mercadante et al., 2025 [101] **Location/Country:** Sicily, Italy	**Aim:** Describe ALS patients admitted to a specialist home palliative care program and their disease trajectory **Study design:** Retrospective cohort	**ALS population:** N = 82 ALS patients; mean age 68 y; 34 female/48 male; bulbar *n* = 33, spinal *n* = 45, mixed *n* = 4; dementia *n* = 17; Karnofsky mean 38. Deceased *n* = 31, living *n* = 51 at study end.	**Care setting/transition anchor:** Admission to specialist home palliative care in a large southern Italian home-care program.	**Care model/continuity component:** Dedicated ALS home palliative team with physicians, nurses, social workers, psychologists, dieticians and speech therapists; external neurology/pneumology consultation.	**Technology/respiratory/nutritional/palliative component:** NIV, mechanical ventilation/tracheostomy, gastrostomy, nasogastric/parenteral nutrition, shared care planning and palliative sedation.	**Outcome measures:** Vital support, ACP/shared care planning, hospital/hospice admissions, withdrawal of support, palliative sedation and home-care duration.	**Main findings:** Home-care duration averaged 287 days in deceased patients and 437 days in those living. No patient had a living will; shared care planning was documented in 21. Vital support increased from 39 at start to 52 during care; NIV use during home care was associated with shared care planning (OR 12.626; *p* = 0.001).	Mechanism/contribution: Home palliative trajectory mechanism: Specialist home palliative care provides longitudinal respiratory/nutritional support and creates opportunities for shared planning. Evidence stratum: Companion report; Mercadante ALS home palliative care program.
**Study:** Schmidt et al., 2025 [102] **Location/Country:** Germany	**Aim:** Analyse type, order and timing of palliative care use in the last year of life and its association with end-of-life quality indicators **Study design:** Retrospective German claims-based sequence-analysis study	**ALS population:** N = 1295 decedents with ALS and no concurrent cancer; 695 (53.7%) received palliative care and 600 did not. Overall median age 73 y (IQR 65–79), 56.1% female, CCI median 3 (IQR 2–5). Palliative care group: Median age 72 y, 59.9% female; no-care group: Median age 74 y, CCI median 4.	**Care setting/transition anchor:** Last 365 days before death; outpatient PPC/SPHC, inpatient palliative care and hospice claims.	**Care model/continuity component:** Temporal Needleman–Wunsch sequence alignment plus hierarchical clustering of palliative care pathways; clusters compared with no-palliative care group.	**Technology/respiratory/nutritional/palliative component:** Claims-based PPC, SPHC, inpatient palliative care (IPC) and hospice coding; sequence timing; emergency, hospital, ICU, PEG, mechanical ventilation and place-of-death indicators.	**Outcome measures:** Palliative care uptake and pathway clusters; 30-day end-of-life quality indicators; place of death.	**Main findings:** Palliative care was used by 695/1295 (53.7%). Within palliative care users: PPC 496 (71.4%), SPHC 400 (57.6%), IPC 138 (19.9%) and hospice 94 (13.5%). Nine clusters were identified. Compared with no palliative care, palliative care users had lower hospital stays (38.0% vs. 60.3%), ICU stays (5.9% vs. 19.7%), emergency service use (29.6% vs. 52.0%), mechanical ventilation (4.6% vs. 19.8%) and hospital deaths (24.3% vs. 53.5%). Late inpatient-only palliative care showed high hospital death (93.2%).	Mechanism/contribution: Palliative pathway-sequencing mechanism not only access but also the timing/combination of PPC, SPHC, IPC and hospice structures end-of-life service use. Evidence stratum: Adjacent continuity evidence; claims-based pathway mapping.
**Study:** Mercadante et al., 2026 [103] **Location/Country:** Sicily, Italy	**Aim:** Assess living will/DAT and ACP among ALS patients admitted to specialized home palliative care, and characterize how ACP emerges during home follow-up **Study design:** Prospective cohort	**ALS population:** N = 68 ALS patients; mean age 68.1+/−8.54 y; 24 female/44 male; bulbar *n* = 19, mixed *n* = 4, spinal *n* = 45; FTD *n* = 16; ALSFRS-R at admission 21.7+/−20.5; diagnosis-to-home palliative care 955+/−941 days.	**Care setting/transition anchor:** Specialized home palliative care after referral in Sicily; end-of-life discussions introduced after admission when acceptable to patient/caregiver.	**Care model/continuity component:** Home palliative team gradually introduced ACP according to sensitivity/readiness; delegates involved when FTD or emergency decisions limited direct expression.	**Technology/respiratory/nutritional/palliative component:** DAT/living will, ACP, tracheostomy/PEG choices, palliative sedation, vital support at admission and end-of-life decision categories.	**Outcome measures:** DAT and prior ACP; ACP during home care; vital support profile; end-of-life choices, deaths and concordance with ACP/delegate decisions.	**Main findings:** No patient had a DAT/living will; only 1/68 (1.5%) had ACP before home palliative care. Vital support at admission: None 40 (58.8%), tracheostomy + PEG 22 (32.3%), NIV 3 (4.4%), NIV + PEG 1 (1.4%), PEG 2 (2.9%). ACP was documented in 21/68 (30.9%) during home care; among ACP patients, tracheostomy was accepted in 15 (71.4%) and refused in 6 (28.6%), PEG was accepted/refused in 10/10, and all agreed to palliative sedation if suffering.	Mechanism/contribution: ACP facilitation mechanism: Specialist home palliative care creates repeated, relationship-based opportunities for end-of-life communication when formal DAT is absent. Evidence stratum: Companion report; ACP-focused Mercadante home palliative care analysis.
**Study:** Mercadante et al., 2026 [104] **Location/Country:** Sicily, Italy	**Aim:** Characterize 6-month clinical, symptom, vital-support and ACP trajectories after admission to specialist ALS home palliative care **Study design:** Prospective longitudinal cohort	**ALS population:** N = 34 consecutive ALS patients; mean age 69.3 y; 21 male; bulbar *n* = 13, spinal *n* = 21; mean diagnosis-to-home care 1003 days; complete/partial awareness *n* = 5/23; FTD *n* = 4; referrals: ALS centres *n* = 21, hospital *n* = 3, GP *n* = 10.	**Care setting/transition anchor:** Home palliative care after referral, with assessments at T0, T2, T4 and T6 months.	**Care model/continuity component:** Regular multiprofessional home visits coordinated by specialist nurse; individualized palliative, respiratory, nutritional, speech, psychosocial, and medical support.	**Technology/respiratory/nutritional/palliative component:** NIV initiation at home, tracheostomy/gastrostomy status, parenteral nutrition, ESAS symptom monitoring, ECAS cognition, ACP/legal administrator and planned hospital transfer for PEG.	**Outcome measures:** Karnofsky, ALS-FRS-R, ESAS symptoms, dysphagia, ECAS, vital support, ACP/living will, deaths and hospital/hospice use.	**Main findings:** Over 6 months, Karnofsky declined from 34.4 to 30.0 (*p* < 0.0005), ALS-FRS-R from 27.2 to 23.1 (*p* < 0.0005), total ESAS from 15.7 to 20.1 (*p* < 0.0005) and dysphagia from 1.1 to 1.4 (*p* < 0.0005). Vital support shifted from none *n* = 19 to *n* = 7 and NIV from *n* = 7 to *n* = 13. ACP increased from *n* = 1 to *n* = 5; no living wills. Seven of 33 evaluable patients died at home; no hospice admissions; hospital use was mainly planned gastrostomy.	Mechanism/contribution: Longitudinal home-care monitoring mechanism tracks functional decline, rising symptom burden, NIV uptake and incremental ACP within home palliative care. Evidence stratum: Companion report; longitudinal Mercadante home palliative care cohort.

Abbreviations and notes: ACP = advance care planning; ALS = amyotrophic lateral sclerosis; ALS-FRS-R = ALS Functional Rating Scale-Revised; ALSAQ-40 = ALS Assessment Questionnaire-40; BPAP = bi-level positive airway pressure; CCI = Charlson Comorbidity Index; DAT = Dichiarazione anticipata di trattamento/living will; ECAS = Edinburgh Cognitive and Behavioural ALS Screen; ER = emergency room; ESAS = Edmonton Symptom Assessment System; FTD = frontotemporal dementia; GP = general practitioner; HBMC = home-based medical care; HMV = home mechanical ventilation; ICU = intensive care unit; MV = mechanical ventilation; NG = nasogastric; NIV = non-invasive ventilation; NR = not reported; PEG = percutaneous endoscopic gastrostomy; pALS = people with ALS; PLS = primary lateral sclerosis; PMA = progressive muscular atrophy; PPC = primary palliative care; QOL = quality of life; SPHC = specialized palliative home care; TAIC = telemedicine-assisted integrated care; TiM = Telehealth in Motor Neuron Disease; IPC = inpatient palliative care; IQR = interquartile range; T0/T2/T4/T6 = baseline/2/4/6 months. ALS is treated as a progressive neurodegenerative disease. The phrase continuous, progression- or needs-triggered continuity pathway is a descriptive sublabel within adjacent continuity and does not create a fifth category or alter counts. The final column uses the standardized evidence categories; companion/secondary reports are retained for distinct information but are not interpreted as independent replication. The disease-by-category distribution is summarized in Table 4.

**Table 4:** **Distribution of included reports by disease group and evidence category.**

Disease Group	Core Transition Evidence	Return-Home/Community Re-Entry Evidence	Adjacent Continuity Evidence	Companion/Secondary Reports	Total
Dementia/ADRD	14 (50.0%)	3 (10.7%)	0 (0.0%)	11 (39.3%)	28
Parkinson’s disease	3 (30.0%)	7 (70.0%)	0 (0.0%)	0 (0.0%)	10
Multiple sclerosis	1 (11.1%)	4 (44.4%)	0 (0.0%)	4 (44.4%)	9
Amyotrophic lateral sclerosis	0 (0.0%)	0 (0.0%)	16 (72.7%)	6 (27.3%)	22
Overall	18 (26.1%)	14 (20.3%)	16 (23.2%)	21 (30.4%)	69

Percentages are calculated within disease rows; overall percentages use all 69 included reports as the denominator. Companion/secondary reports contributed distinct information from overlapping programs or cohorts and were not interpreted as independent replication.

Respiratory continuity was found to be a dominant ALS mechanism. Home non-invasive ventilation (NIV) telemonitoring was associated with fewer office and emergency visits and hospital admissions while supporting ventilator setting review [83]. Technical and clinical tele-assistance studies reported that home-ventilation data, oximetry, nurse-tutor calls and standardized telephone risk cards could support home monitoring, triage and escalation after discharge or during home follow-up [84,85,86]. At-home NIV initiation with telemonitoring was associated with faster access to ventilation than hospital initiation and provided early adherence and nocturnal oxygenation monitoring [97].

Multidisciplinary and telehealth studies addressed access to specialist ALS care in the home or community. Case management evidence was mixed: A cluster randomized trial found no clear additive benefit over established multidisciplinary care, whereas qualitative data showed that patients, caregivers and professionals valued proactive home contact, time, emotional support and navigation when usual care was less responsive or disease progression was rapid [87,88]. Store-and-forward telemedicine, app-based monitoring, COVID-era telemedicine, multidisciplinary video visits and nationwide implementation of ALS home monitoring and coaching showed that remote models can be feasible and acceptable, but they require workflow integration, usability, caregiver participation and clear escalation pathways [90,91,92,93,94,95,96,98,99,100].

Palliative and end-of-life continuity formed a further ALS pathway. Home neuropalliative and home palliative care cohorts described multiprofessional home visits, respiratory and nutritional support, shared planning, symptom monitoring, home death, hospital avoidance and gradual advance-care-planning conversations [99,101,103,104]. Claims-based sequence analysis showed that palliative care pathways differed not only by access but also by timing and service combination; pathways involving specialized palliative home care and hospice were associated with fewer emergency, hospital and intensive-care indicators than no palliative care or late inpatient-only palliative care [102]. Qualitative transition evidence added the lived experience of device adoption, caregiver role change, social isolation, communication loss and the need for earlier information about future decisions [94].

### 3.6. Cross-Cutting Care Models, Outcomes and Implementation Themes

**Figure 2:**
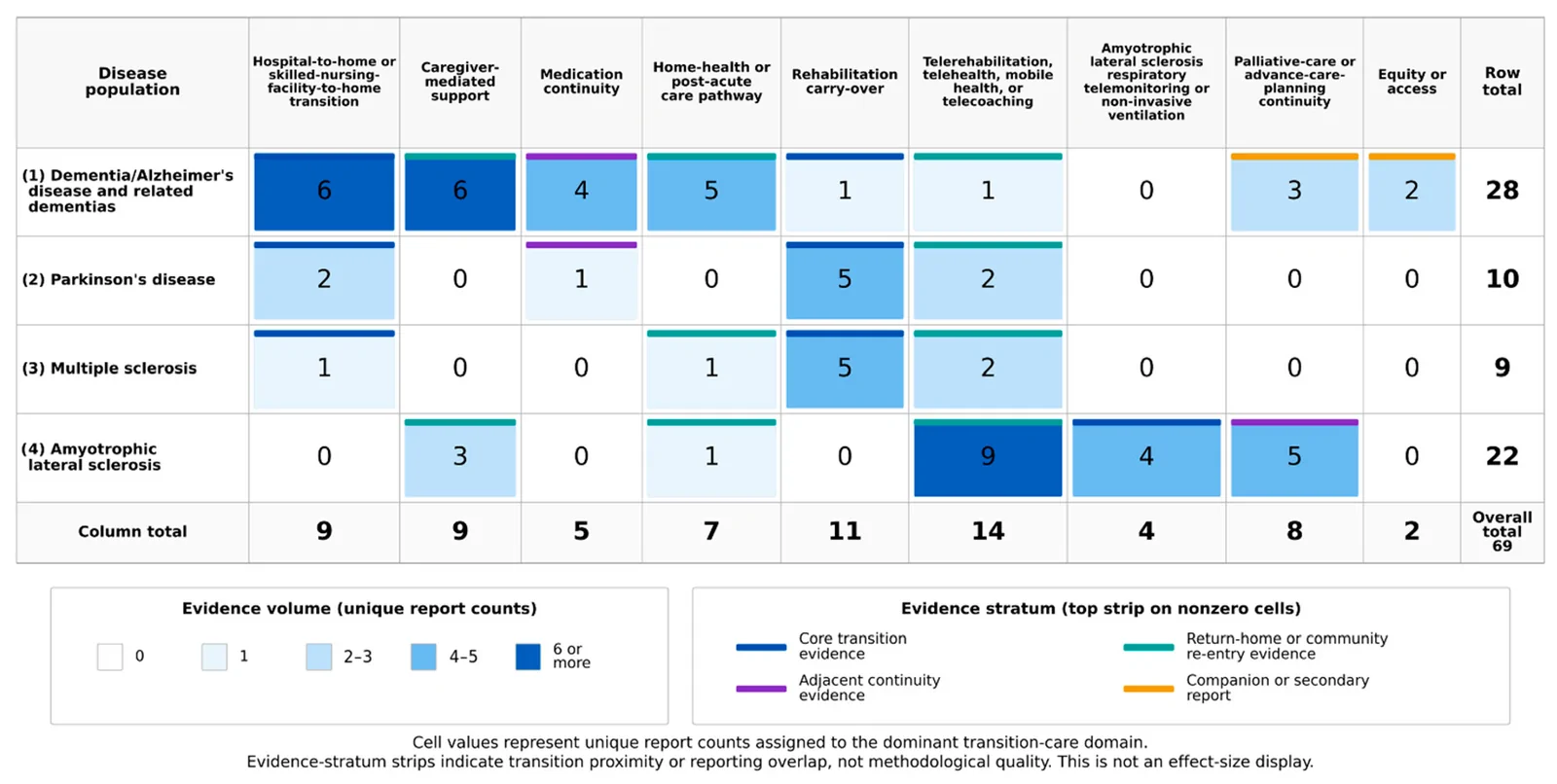
Evidence map by disease population and transition-care domain. Cell values are unique report counts assigned to the dominant transition-care domain, with each included report counted once. Background intensity indicates mapped evidence volume, and the colour strip indicates evidence stratum. The evidence stratum indicates transition proximity or reporting overlap, not methodological quality. Row totals equal the 69 included reports; this is not an effect-size display. Data are based on the included reports [21,22,24,25,40,41,42,43,44,45,46,47,48,49,50,51,52,53,54,55,56,57,58,59,60,61,62,63,64,65,66,67,68,69,70,71,72,73,74,75,76,77,78,79,80,81,82,83,84,85,86,87,88,89,90,91,92,93,94,95,96,97,98,99,100,101,102,103,104].

The cross-cutting map indicated disease-specific emphases rather than a single uniform transition pathway. Dementia/ADRD reports emphasized caregiver-mediated safety, medication management, documentation quality, home-health risk stratification, palliative planning, and equity. PD and MS reports emphasized rehabilitation carry-over, mobility, medication timing, fatigue, self-management, and functional home stabilization. ALS reports emphasized respiratory, nutritional, technological, and palliative continuity. Figure 2 organizes these domains and their evidence classifications.

Concrete category assignments further illustrate these boundaries. The study of Freeman et al. [21] was classified as return-home/community re-entry evidence because it examined the carry-over of inpatient rehabilitation after discharge; the study of Cummings et al. [40] as core transition evidence because discharge-plan adequacy and readmission were explicitly anchored to dementia hospitalization; that of Berish et al. [58] as a companion/secondary report because it provided distinct outcomes from the Fam-FFC parent cohort rather than independent replication; and that of Pinto et al. [83] as adjacent continuity evidence because home NIV telemonitoring represented ongoing specialist support without a discrete discharge-defined intervention (Table 1, Table 2 and Table 3; Supplementary Table S3).

Outcome domains were heterogeneous and were not statistically pooled. Safety and service use were commonly captured through readmission, emergency department use, hospital utilization, intensive-care use, falls, adverse events and home-health outcomes. Rehabilitation outcomes included activities of daily living, walking capacity, mobility, balance, fatigue, physical activity, goal carry-over and quality of life. Caregiver outcomes included preparedness, strain, burden, anxiety, depression, satisfaction, role ambiguity, medication-management confidence and navigation workload. Implementation outcomes included feasibility, acceptability, fidelity, adherence, technology usability, workforce burden, cost, access and sustainability. Detailed care-model, outcome-domain, technology-enabled continuity, caregiver-role, and methodological-gap mappings are provided in Supplementary Tables S4–S8.

**Figure 3:**
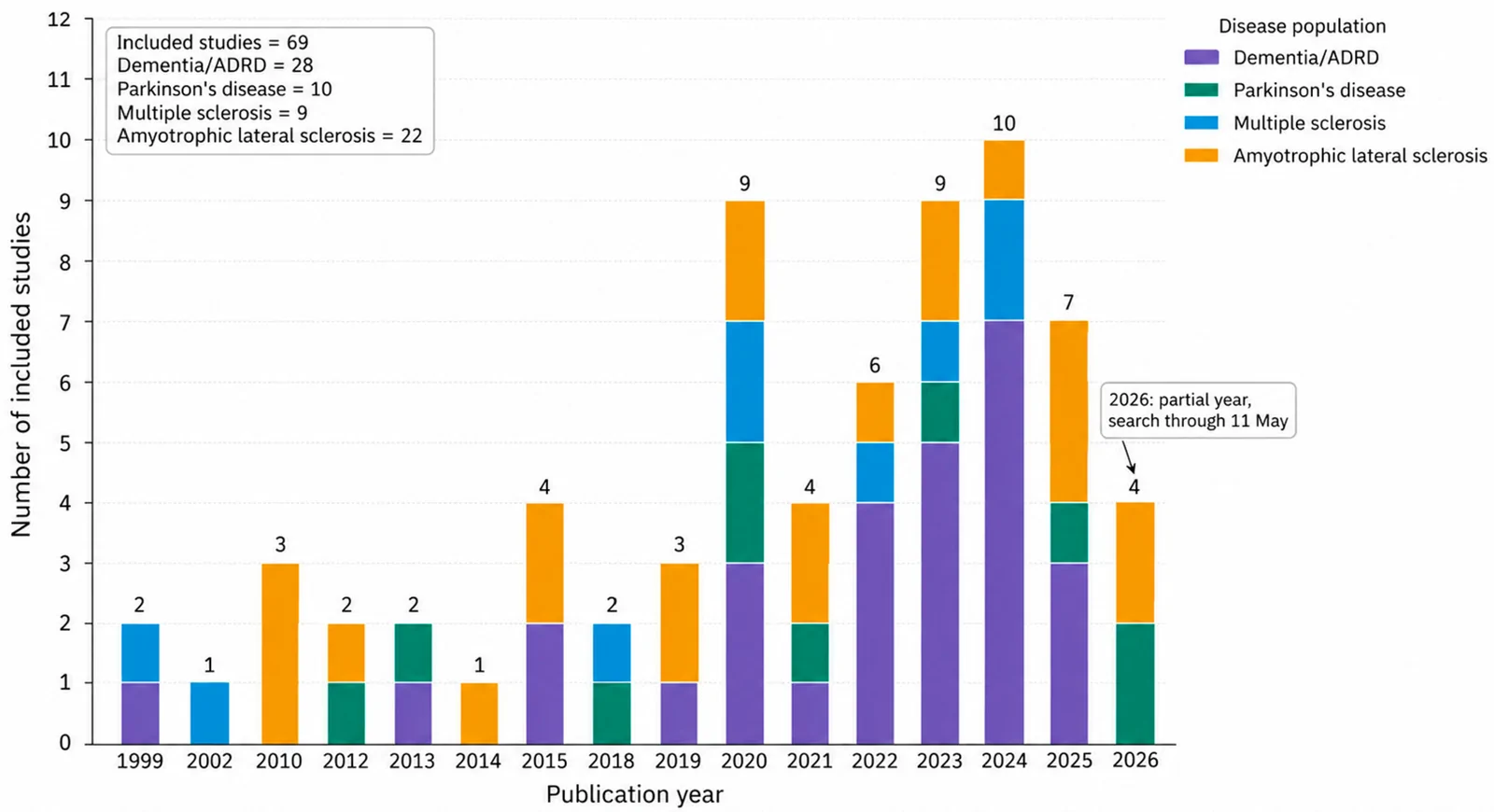
Temporal distribution of included studies by disease population. Stacked bars show included reports by publication year and disease group. Counts represent reports, not effect sizes or methodological quality. The 2026 count reflects the final search update through 11 May 2026.

Supplementary Figures S1 and S2 present the proposed, author-derived conceptual frameworks, organizing the post-discharge trajectory and the disease-specific risks, continuity mechanisms, outcomes, and gaps. The frameworks were developed through framework-based synthesis of charted mechanisms, disease-specific risks, transition stages, and recurrent implementation barriers. Neither represents an empirically validated care model, taxonomy, effect-size analysis, or clinical prediction tool. A component-to-study traceability matrix is provided in Supplementary Table S9. Most included reports were published from 2020 onward, with recent growth particularly visible in dementia/ADRD and ALS continuity research (Figure 3).

## 4. Discussion

### 4.1. Principal Findings and Disease-Specific Transition Signatures

This scoping review mapped a heterogeneous literature on post-discharge follow-up, transitional care, return-home support, rehabilitation continuity, and community re-entry across dementia/ADRD, PD, MS, and ALS. Its main contribution is not to duplicate disease-specific syntheses, but to compare selected chronic neurological conditions through a shared framework of transition proximity, continuity mechanisms, caregiver work, equity, implementation, and reporting overlap. The map suggests that neurological transition care extends beyond readmission prevention to information transfer, treatment continuity, preservation of rehabilitation gains, caregiver preparedness, monitoring and escalation, and accountable linkage with home and community services. These patterns are descriptive and hypothesis-generating rather than estimates of comparative effectiveness.

The strongest discharge-anchored conclusions arise from dementia/ADRD, which contributed most core transition evidence. These reports consistently located caregiver preparation, medication understanding, discharge documentation, home-health linkage, and palliative planning within the practical work of hospital-to-home or SNF-to-home transfer. This interpretation is consistent with broader dementia-transition literature emphasizing that transitions are experienced by patient–caregiver dyads and that caregivers frequently provide the only continuous link across fragmented services [105,106,107,108].

PD and MS findings carry a different inferential weight. Most reports addressed return-home functional continuity or rehabilitation carry-over, not comprehensive discharge pathways. In PD, the mapped vulnerabilities included medication timing, mobility readiness, falls risk, delirium risk, functional decline, and home-to-home stability; these observations align with post-discharge home-health and medication-safety literature [109,110,111,112,113,114]. In MS, the dominant question was whether rehabilitation gains, fatigue management, physical activity, self-management, and participation persisted after inpatient care, consistent with contemporary rehabilitation and remote-exercise priorities [115,116,117,118]. These findings support development of transition-specific interventions but do not establish the effectiveness of a unified PD or MS discharge model.

ALS represents the clearest example of adjacent continuity evidence. The mapped reports support the relevance of home specialist access, respiratory and nutritional monitoring, telehealth, caregiver escalation, palliative continuity, and advance care planning, in agreement with contemporary ALS guidance and reviews [119,120,121,122,123]. Their repeated activation as function or needs change is captured by the descriptive sublabel continuous, progression- or needs-triggered continuity pathway. Because this remains within adjacent continuity, these studies should not be interpreted as direct tests of discharge-model effectiveness. They instead identify components, feasibility conditions, and escalation functions that future discharge-anchored pathways could incorporate.

### 4.2. Continuity Mechanisms, Theoretical Alignment, Caregiver Work, and Equity

The mapped mechanisms correspond to established functions in transitional-care and integrated-care theory. Coleman’s continuity framework and Naylor’s care-span model emphasize accountable transfer across settings, medication continuity, patient and caregiver preparation, and timely follow-up [2,3,4]. Integrated, patient-centred network care similarly depends on coordination among specialists, rehabilitation professionals, primary care, and community services [10]. Within the Medical Research Council framework for complex interventions, the observed mechanisms can be understood as interacting components whose effects depend on context, implementation, and recipient capacity [124]. This theoretical alignment supports their use as organizing constructs for future study, but it does not validate the author-derived conceptual model.

Several mechanisms appeared genuinely cross-cutting. Information transfer, medication or treatment continuity, caregiver preparedness, rehabilitation carry-over, monitoring and escalation, home-health or community linkage, and equity/access considerations recurred across disease groups, although their content differed by trajectory. Discharge documents, medication lists, referrals, rehabilitation goals, palliative plans, and escalation instructions must be understandable and actionable for the people who use them. In PD, medication continuity included precise dopaminergic timing; in dementia/ADRD, it required caregiver-facing plans for safe administration; in ALS, it included respiratory and nutritional support that could be monitored and adjusted at home. In PD and MS, rehabilitation gains often required home exercise, coaching, outpatient support, mHealth, telecoaching, or community linkage to become sustained daily performance.

Disease-specific features indicate that a single generic continuity model is unlikely to be sufficient. Dementia/ADRD models must account for cognitive impairment, caregiver-mediated safety, documentation quality, medication support, home-health access and palliative planning. PD pathways require attention to medication timing, mobility readiness, falls and delirium risk, and home-to-home stability. MS pathways should address fatigue, rehabilitation carry-over, participation, self-management and the capacity-to-performance gap after inpatient rehabilitation. ALS pathways require respiratory/NIV monitoring, nutritional support, telemonitoring, equipment management, palliative continuity, advance care planning and caregiver escalation. These differences help explain why future systematic reviews will need more standardized transition anchors and outcome domains before meaningful effectiveness synthesis is possible.

Caregiver work was prominent in dementia/ADRD and ALS and was also relevant in advanced PD and MS. The included evidence shows how access constraints may enter the pathway: Dementia discharge-plan adequacy was compromised when resources were unavailable or unaffordable [40]; claims analyses identified differences by race/ethnicity, dual eligibility, and home-health agency quality [56,65]; and when Latino caregivers described culturally shaped roles, ambiguous responsibility, and dissatisfaction with generic resources [66]. Fam-FFC reports included racially diverse samples, but small underrepresented groups were not analysed separately, and race or education were generally covariates rather than prespecified equity outcomes [57,58]. Other reports relied on caregiver-reported outcomes without measuring financial strain or broader social determinants [59]. In ALS, discomfort with smartphone/video use [93], and in a Dutch monitoring program the high internet-use context [96], signalled digital-access and generalizability constraints rather than formal equity analyses. PD, MS, and ALS studies rarely tested differential reach, uptake, burden, or outcomes across socioeconomic, geographic, linguistic, cultural, disability, or caregiver-capacity strata.

For clinicians, discharge planners, and health systems, neurological transition planning should combine disease-specific continuity checklists with caregiver-facing information, medication or treatment reconciliation, rehabilitation carry-over plans, predefined monitoring and escalation routes, and early linkage to home-health, rehabilitation, respiratory, nutritional, or palliative services. Table 5 translates these mapped mechanisms into minimum actionable components while retaining disease-specific and equity safeguards.

### 4.3. Strengths, Limitations and Future Directions

Strengths include a prospectively registered protocol, PCC-based eligibility, PRISMA-ScR-informed reporting, broad database searching, independent screening, verified data charting, disease-specific evidence tables, and an explicit evidence-classification framework. The cross-disease approach enabled shared continuity functions to be identified while preserving distinctions among core transition, return-home, adjacent, and companion evidence.

The review also has important limitations. Transition anchors, disease stage, intervention dose, comparator design, follow-up duration, and outcome measurement were heterogeneous. Formal risk-of-bias assessment and certainty grading were not undertaken, so the map cannot establish the reliability or magnitude of intervention effects. The evidence categories indicate transition proximity and reporting overlap, not methodological quality, sample size adequacy, comparator strength, or certainty; absolute counts should therefore not be interpreted as evidence strength. Companion and secondary reports enriched mechanistic interpretation but may overrepresent particular programs. The exclusion of grey literature and non-English reports may have introduced selection bias, especially because service-delivery models are often reported in local policy documents, implementation reports, theses, or non-English health-system literature. Excluded studies and inaccessible reports may contain alternative perspectives on service organization or equity. Although the continuous, progression- or needs-triggered sublabel improves the description of ALS, it does not convert adjacent evidence into direct discharge-model evidence. Participation, caregiver workload, equity, implementation cost, and long-term community stability were often undermeasured.

Future studies should define the transition origin, destination, and intervention start point; specify active components, dose, personnel, caregiver role, and escalation rules; use shared outcome domains and longer follow-up; and report equity and implementation measures. Dementia/ADRD research should prioritize dyad-centred discharge models; PD research should test medication-safety and mobility-readiness pathways; MS research should evaluate structured rehabilitation aftercare; and ALS research should embed respiratory, nutritional, telehealth, and palliative components within explicitly discharge-anchored designs. Future systematic reviews and trials will require standardized outcomes, comparable follow-up periods, stronger comparators, and clearer reporting of intervention components.

Equity-focused research should prespecify recruitment and subgroup analyses by race/ethnicity, socioeconomic position, rurality or travel burden, language, health and digital literacy, disability and communication needs, caregiver availability, and insurance or service entitlement. Studies should report who is approached, enrolled, retained, and able to use each delivery mode; compare digital, telephone, in-person, and hybrid options; and measure affordability, equipment and broadband access, interpreter or accessibility support, caregiver time and financial strain, and differential outcomes. Community-based participatory design with patients and caregivers from underrepresented groups should inform adaptation. Implementation evaluations should use equity-sensitive reach, adoption, fidelity, burden, and sustainability indicators and test whether resource navigation, loaned technology, transport, language-concordant materials, or alternative non-digital pathways reduce observed gaps.

## 5. Conclusions

The mapped evidence suggests that return home after neurological hospitalization, rehabilitation, or specialist care is a clinically vulnerable trajectory rather than a single administrative event. Across diseases, recurrent continuity functions included caregiver preparation, medication or treatment continuity, rehabilitation carry-over, home-service linkage, monitoring and escalation, respiratory and nutritional support, palliative continuity, advance care planning, and equitable access. Their apparent recurrence does not establish comparative effectiveness because the underlying evidence was heterogeneous.

The empirical map was disease specific. Dementia/ADRD provided the most direct discharge-anchored evidence; PD and MS mainly informed functional return home and rehabilitation continuity; and ALS primarily informed adjacent home-based specialist, respiratory, telehealth, and palliative continuity. Conclusions should therefore retain the inferential distinctions created by the evidence-classification framework.

The proposed conceptual frameworks in Supplementary Figures S1 and S2 are author-derived syntheses of mapped findings, not empirically validated models, taxonomies, or clinical prediction tools. They organize hypotheses about how continuity mechanisms may interact across settings and disease trajectories; Supplementary Table S9 makes their study-level basis explicit. Prospective studies should test these mechanisms using explicit transition anchors, stronger comparators, standardized patient and caregiver outcomes, longer follow-up, and equity-sensitive implementation measures.

## Figures and Tables

**Table 1:** **Post-discharge, hospital-to-home and caregiver-mediated transition evidence in dementia/Alzheimer’s disease and related dementias.** This table summarizes studies in which discharge planning, hospital-to-home transition, skilled nursing facility-to-home transition, caregiver preparedness, medication continuity, home health care, post-acute care, palliative transition, or return-home support represented the main focus of the study.

Author, Year [Ref.]/Location/Country	Aim and Study Design	Population Characteristics	Discharge or Transition Context	Care Model or Transition Component	Outcome Measures	Main Findings	Caregiver/Implementation/Equity or Access Component	Key Transition Mechanism and Contribution to the Evidence Map
**Study:** Cummings et al., 1999 [40] **Location/Country:** USA	**Aim:** Measure discharge-plan adequacy and its relationship with rehospitalization **Study design:** Prospective observational study	**Population:** N = 131 dementia patients discharged from a geriatric neuropsychiatry unit; female 61.1%; White 84.7%; widowed 52.7%; primary diagnosis dementia with agitation 73.3%; mean LOS 15.5 days (SD 9.6; range 3–70).	**Discharge/transition context:** Hospital discharge after neuropsychiatric admission; 1-month post-discharge caregiver follow-up.	**Care model/transition component:** Social-worker-rated aftercare plan adequacy plus caregiver telephone follow-up; assessment of patient, family and resource-related discharge factors.	**Outcome measures:** Discharge-plan adequacy; 1-month readmission; patient, caregiver and resource predictors.	**Main findings:** 35.1% of plans were rated less than adequate/very inadequate; 24/131 patients (18.3%) were readmitted within 1 month. Lower adequacy ratings, male sex and insufficient caregiver support predicted readmission.	Caregiver: Adult child 52.7%; spouse 14.5%; denial, unrealistic expectations, and insufficient support compromised planning. Implementation: Plan adequacy depended on dementia-specific placement and available resources. Equity/access: Unavailable or unaffordable resources; predominantly White sample.	Mechanism/contribution: This study maps family readiness, caregiver support and resource availability as determinants of safe aftercare planning. Evidence stratum: Core transition evidence.
**Study:** Villars et al., 2013 [41] **Location/Country:** Toulouse, France	**Aim:** Assess a post-discharge follow-up intervention after discharge from a special Alzheimer acute care unit **Study design:** Quasi-experimental before-after study	**Population:** N = 390 severely demented patients in 2008–2009; mean age 81.79 years (SD 7.37); women 59.97%; AD/mixed dementia 83.38%; mean MMSE 12.34; MMSE < 10 in 37.75%; malnutrition 41.07% and malnutrition risk 50.00%.	**Discharge/transition context:** Discharge from SACU; baseline comparison year 2007 vs. intervention years 2008 and 2009.	**Care model/transition component:** Individualized care plan targeting BPSD, nutrition, function, living arrangements and treatment; calls to caregiver/resource person during week 1, before month 1, and at 3 and 6 months; optional medical/psychological/social consultation.	**Outcome measures:** ER rehospitalization within 31 days; rehospitalization to any department at 31 days and 3 months; BPSD and follow-up activity.	**Main findings:** Early ER rehospitalization decreased non-significantly from 8.39% to 8.02% and 7.47%; 3-month all-department rehospitalization decreased from 28.98% to 24.03% and 23.58%. Vocal disruptive behaviour was more frequent in rehospitalized patients (9.64% vs. 3.97%; *p* = 0.05).	Caregiver: Caregiver/resource person was the central follow-up contact. Implementation: Telephone contact was more frequent among rehospitalized patients, suggesting greater complexity rather than a clear effect. Equity/access: NR.	Mechanism/contribution: Telephone follow-up mechanism: Scheduled caregiver/resource-person contact after SACU discharge targets BPSD, nutrition, treatment changes and living-arrangement instability. Evidence stratum: Core transition evidence.
**Study:** Kable et al., 2015 [42] **Location/Country:** New South Wales, Australia	**Aim:** Explore health-professional perspectives on discharge and transitional-care system failures **Study design:** Qualitative descriptive focus-group study	**Population:** N = 33 professionals in 4 focus groups: Junior medical officers *n* = 5, acute discharge-planning nurses/allied health *n* = 16, RACF staff *n* = 4, and GPs/practice staff *n* = 8; sessions lasted 35–90 min.	**Discharge/transition context:** Hospital discharge and transfer of people with dementia to home, GP/community services or RACF care.	**Care model/transition component:** No intervention; analysis of discharge-planning barriers, communication failures, documentation gaps, service-access problems and medication-order issues.	**Outcome measures:** Qualitative barriers/facilitators; documentation and medication-order failures; post-discharge service delivery; adverse events, readmission and carer stress.	**Main findings:** Two major themes were identified: Acute-care barriers to effective discharge planning and community/RACF transitional-care process failures. Reported failure modes included early-discharge pressure, long waits for community services, incomplete discharge summaries, poor notification, missed services and medication-order problems.	Caregiver: Carers acted as de facto care managers. Implementation: Gaps included 24 h dementia support, dose administration aids, medicines review, and GP/RACF coordination. Equity/access: Limited service access was reported; no subgroup equity analysis.	Mechanism/contribution: System-failure mechanism identifies discharge pressure, documentation gaps, medication-order problems, insufficient community service capacity and fragmented communication as transition hazards. Evidence stratum: Core transition evidence.
**Study:** Boltz et al., 2015 [43] **Location/Country:** USA	**Aim:** Test feasibility and preliminary efficacy of dementia-capable Fam-FFC **Study design:** Comparative repeated-measures study	**Population:** N = 86 dyads completed baseline/discharge follow-up: Intervention *n* = 44, control *n* = 42; patients mean age 82.4 years (SD 7.6); female 59%; admitted from home 98%; Black 45% and White 45%; delirium on admission 42%.	**Discharge/transition context:** Acute medical hospitalization with outcomes through 14 and 60 days after discharge.	**Care model/transition component:** Family-centred function-focused care delivered by a family-centred resource nurse 10–15 h/week; environmental/policy assessment, staff education, individualized functional goals, family education and post-acute follow-up.	**Outcome measures:** Barthel ADL, walking performance, Tinetti gait/balance, delirium severity, 30-day readmission, return to baseline ADL, caregiver preparedness, anxiety, depression, strain and mutuality.	**Main findings:** Fam-FFC was associated with better ADL (*p* = 0.02), walking performance (*p* = 0.001), lower delirium severity (*p* = 0.03), fewer 30-day readmissions (*p* = 0.02) and higher return to baseline ADL at 2 months (*p* = 0.003); no gait/balance effect.	Caregiver: Preparedness increased and anxiety decreased; depression, strain, and mutuality did not differ significantly. Implementation: Fidelity was high: 88% staff-care-plan and 92% caregiver-log agreement. Equity/access: NR.	Mechanism/contribution: Family-function mechanism embeds family caregivers in function-focused goals and post-acute recovery planning to reduce hospital-acquired disability and support ADL/walking recovery. Evidence stratum: Companion/secondary report; early Fam-FFC report, not an independent cohort
**Study:** Kable et al., 2019 [44] **Location/Country:** New South Wales, Australia	**Aim:** Audit discharge documentation for people with dementia discharged home **Study design:** Cross-sectional pilot study with carer survey	**Population:** Of 313 dementia discharges, 73 (23%) were eligible; 72 discharged home and 1 to participating RACF; mean age 81 years; female 55%; surgical wards 23%, medical wards 48%, short-stay units 29%.	**Discharge/transition context:** Hospital discharge home/RACF with GP and patient/carer discharge documentation.	**Care model/transition component:** Audit of medical/nursing/allied-health discharge summaries against expected criteria plus post-discharge carer survey.	**Outcome measures:** Documentation compliance; readmission within 28 days and 3 months; ED presentation within 3 months; carer strain.	**Main findings:** Medical discharge summary was present in 92%, nursing summary in 70% and allied-health summary in 3%. Readmission occurred in 6% within 28 days and 19% within 3 months; ED presentation occurred in 26% within 3 months.	Caregiver: Carer information and support-group guidance were often missing from discharge documentation. Implementation: Compliance gaps affected risk assessment, medication aids, referrals, appointments, support groups, and advance planning. Equity/access: NR.	Mechanism/contribution: Documentation mechanism shows that dementia discharge documents may omit risk, medication, referral, caregiver-strain and advance-care information needed for safe transition. Evidence stratum: Core transition evidence.
**Study:** Bruce et al., 2020 [45] **Location/Country:** New South Wales, Australia	**Aim:** Evaluate carer strain and medication coping after discharge within the SMS Dementia Study **Study design:** Prospective quasi-experimental pre/post-controlled trial	**Population:** N = 88 carers completed 3-month telephone surveys; consent rate 156/523 (29.8%) among people with dementia admitted from home; carers mostly female (77%); mean carer age ranged 61–68 years across site/phase groups.	**Discharge/transition context:** Unplanned hospital admission and discharge home for people with dementia/cognitive impairment.	**Care model/transition component:** Safe Medication Strategy Dementia bundle: Medication reconciliation, carer needs assessment, DAA training/arrangement, medication list/explanations, GP contact about medication changes and HMR recommendation.	**Outcome measures:** Carer medication concerns and difficulties; DAA use; HMR receipt; knowledge of medication indications; Modified Caregiver Strain Index.	**Main findings:** At 3 months, 33% of carers were concerned about managing medications and 40% reported difficulties; 32.4% of those reporting difficulties described problems obtaining cooperation with medication-taking. DAA use was 72%; only 15% reported recent HMR; high carer strain was reported by 74%.	Caregiver: Medication safety was largely caregiver mediated. Implementation: Dose administration aid use increased from 56% to 72%, without statistical significance. Equity/access: NR.	Mechanism/contribution: Medication-safety mechanism maps caregiver vigilance, DAA use, HMR underuse and medication-management burden as central dementia discharge risks. Evidence stratum: Core transition evidence.
**Study:** Knox et al., 2020 [46] **Location/Country:** USA	**Aim:** Examine dementia severity and 30-day potentially preventable readmissions during home health **Study design:** Retrospective Medicare/OASIS cohort study	**Population:** N = 126,292 Medicare beneficiaries with dementia admitted to home health after acute/psychiatric hospitalization; 65% aged >81 years; 61% female; 80% White.	**Discharge/transition context:** Home health admission within 5 days after hospital discharge.	**Care model/transition component:** OASIS-derived dementia severity categories cross-walked to FAST stages.	**Outcome measures:** 30-day potentially preventable readmission during home health.	**Main findings:** Overall 30-day PPR rate was 7.6%. PPR rates were highest in dementia severity category 7 (12.6%) and category 6 (8.8%); adjusted ORs vs. category 1/2 were 1.37 (95% CI 1.29–1.46) for category 6 and 1.94 (95% CI 1.64–2.31) for category 7.	Caregiver: Caregiver support was captured in routine home-health assessment. Implementation: OASIS data enabled potentially preventable readmission risk stratification. Equity/access: Readmission was higher among Medicare–Medicaid dual-eligible patients.	Mechanism/contribution: Home-health risk mechanism uses dementia severity staging to identify patients who may need specialized support after hospital-to-home-health transfer. Evidence stratum: Companion/secondary report; Knox Medicare/OASIS home-health series.
**Study:** Knox et al., 2020 [47] **Location/Country:** USA	**Aim:** Identify mobility, self-care, cognition and caregiver-support predictors of readmission during home health **Study design:** Retrospective Medicare/OASIS cohort study	**Population:** N = 118,171 Medicare beneficiaries with ADRD receiving home health after hospitalization; 65% aged ≥81 years; 61% female; 80% White.	**Discharge/transition context:** Home health admission after acute/psychiatric hospitalization.	**Care model/transition component:** OASIS-derived mobility, self-care, cognition and caregiver-support domains at home-health start of care.	**Outcome measures:** 30-day potentially preventable readmission during home health; common PPR diagnoses.	**Main findings:** Overall PPR rate was 7.6%. Fully adjusted odds were higher for the most dependent mobility quartile (OR 1.59; 95% CI 1.47–1.71), most dependent self-care quartile (OR 1.73; 95% CI 1.61–1.87), unmet caregiver needs (OR 1.11; 95% CI 1.05–1.17) and cognitive impairment (OR 1.23; 95% CI 1.16–1.30).	Caregiver: NR as a distinct outcome. Implementation: Mobility and self-care dependence were the main actionable risk domains. Equity/access: NR.	Mechanism/contribution: Functional-caregiver risk mechanism links mobility, self-care, cognition and unmet caregiver support to preventable readmission risk during post-acute home health. Evidence stratum: Companion/secondary report; Knox Medicare/OASIS home-health series.
**Study:** Sawan et al., 2021 [48] **Location/Country:** Australia	**Aim:** Explore caregiver experiences of medication-management advice at discharge **Study design:** Qualitative semi-structured telephone interview study	**Population:** N = 31 caregivers across Australia; female 81%; median caregiver age 60 years; adult child 65%, spouse/partner 29%; care recipients median age 81 years; community dwelling 65%, long-term care 32%; median 4 months from admission to interview.	**Discharge/transition context:** Hospital discharge medication guidance for people living with dementia.	**Care model/transition component:** Interviews on discharge medication resources, caregiver involvement, medication administration/supply and support needs.	**Outcome measures:** Qualitative themes on medication information, caregiver engagement, supply continuity and caregiver recommendations.	**Main findings:** Three themes were identified: Inadequate information about medication management at discharge, limited caregiver engagement in medication decisions, and difficulty ensuring medication supply post-discharge.	Caregiver: Caregivers requested tailored medication lists, adverse-effect information, and earlier discharge discussion. Implementation: Needed better communication with community pharmacists and primary-care physicians. Equity/access: NR.	Mechanism/contribution: Medication-continuity mechanism identifies information deficits, limited caregiver engagement and medication-supply disruption as dementia discharge safety gaps. Evidence stratum: Core transition evidence.
**Study:** Knox et al., 2022 [49] **Location/Country:** USA	**Aim:** Assess dementia severity, caregiver support and early discharge from home-health therapy **Study design:** Retrospective Medicare/OASIS study	**Population:** N = 91,302 Medicare beneficiaries with ADRD discharged to the community after home health; 65.6% aged ≥81 years; 61.5% female; 82.6% White.	**Discharge/transition context:** Home-health episode after acute/psychiatric hospitalization; completed community discharge episode.	**Care model/transition component:** Comparison of planned vs. billed PT/OT/ST visits; OASIS dementia severity, caregiver support and oral medication management variables.	**Outcome measures:** Early discharge from therapy services, defined as missing >2 planned therapy visits.	**Main findings:** Overall early discharge rate was 10.4% (95% CI 10.2–10.6). No consistent early-discharge pattern across dementia severity levels; highest observed rate was category 4 (16.4%) and lowest category 6 (9.6%).	Caregiver: Medication assistance and unmet caregiver needs signalled greater care complexity. Implementation: Visible complexity may prolong home-health therapy episodes. Equity/access: NR.	Mechanism/contribution: Therapy-completion mechanism compares planned versus delivered therapy to map how medication assistance and caregiver need affect continuity of home-health rehabilitation. Evidence stratum: Companion/secondary report; Knox Medicare/OASIS home-health series.
**Study:** Knox et al., 2022 [50] **Location/Country:** USA	**Aim:** Identify home-health utilization factors associated with modified successful discharge to community **Study design:** Retrospective national Medicare cohort	**Population:** N = 535,691 home-health patients after hospitalization; 94,497 (18.0%) had ADRD; overall cohort 42% aged ≥81 years, 62% female, 85% White.	**Discharge/transition context:** Home health after index hospitalization; outcome assessed after discharge from home health to community.	**Care model/transition component:** Home-health utilization: PT, OT, ST, skilled nursing, home-health aide services, episode length, visit volume and early therapy discharge.	**Outcome measures:** Modified successful discharge to community: Community discharge without readmission or death within 31 days.	**Main findings:** Overall M-SDC was 92.1%; M-SDC was lower among patients with ADRD (88.5%). M-SDC likelihood increased with PT, episode length > 15 days and >10 therapy visits; it decreased with ST, skilled nursing, home-health aide services and early therapy discharge.	Caregiver: Caregiver support was included in adjusted analyses. Implementation: Longer episode duration was associated with the ADRD utilization pattern. Equity/access: NR.	Mechanism/contribution: Home-health utilization mechanism maps therapy type, visit volume, episode duration and early discharge as service-delivery factors shaping community stability after home health. Evidence stratum: Companion/secondary report; Knox Medicare/OASIS home-health series.
**Study:** Toles et al., 2022 [51] **Location/Country:** USA	**Aim:** Describe unique care needs during SNF-to-home/assisted-living transitions **Study design:** Qualitative study	**Population:** N = 39 participants: 22 SNF staff, 4 home-health nurses, 10 people with dementia and 10 family caregivers; 4 SNFs in one organization; PWD–caregiver interviews included 8 discharges home and 2 to assisted living.	**Discharge/transition context:** Skilled nursing facility discharge to home or assisted living after short-stay rehabilitation.	**Care model/transition component:** Needs assessment to adapt Connect-Home transitional care for dementia-specific transition needs.	**Outcome measures:** Qualitative themes on readiness, symptom management, community supports and caregiver support.	**Main findings:** Four care needs were identified: Limited readiness for dementia care planning in the SNF, caregiver unpreparedness for dementia symptoms at home, difficulty connecting dyads to community supports, and limited support for caregiver needs.	Caregiver: Caregivers managed medication resistance, pain, ADLs, and falls; three reported early post-return falls. Implementation: Community-support linkage was inconsistent. Equity/access: Cost and service access limited linkage.	Mechanism/contribution: SNF-to-home needs mechanism identifies readiness, symptom-management training, community linkage and caregiver support as adaptation targets for dementia transitional care. Evidence stratum: Core transition evidence; precursor/needs-assessment report for Connect-Home ADRD.
**Study:** Toles et al., 2022 [52] **Location/Country:** USA	**Aim:** Assess fidelity, acceptability and preliminary outcomes of Connect-Home ADRD **Study design:** Feasibility study	**Population:** N = 19 PWD–caregiver dyads enrolled from 34 eligible dyads (56%) in 2 SNFs; PWD female 79% and White 94.7%; caregivers female 68.4%, White 94.7%, adult child 79.0%.	**Discharge/transition context:** SNF discharge to own home (57.9%), assisted living (31.6%) or caregiver home (10.5%).	**Care model/transition component:** Two-step transitional care: SNF transition plan, care planning, medication list and provider handoff; home-care nurse within 24 h; dementia caregiving specialist calls 3 times over 4 weeks.	**Outcome measures:** Fidelity, acceptability, CTM-15, Preparedness for Caregiving Scale, 30-day hospital/ED use and mechanism of support.	**Main findings:** Fidelity was high: Complete transition plan 94.7%, care-plan meeting 100%, follow-up appointment 84.2%, records transmitted 100%, home care within 24 h 84.2%, 3 support calls 89.5%. Two readmissions and one death/hospice transition occurred within 30 days; no ED-only use.	Caregiver: Support targeted communication, symptoms, and level-of-care decisions; preparedness was measured. Implementation: Feasibility measures included CTM-15 and caregiver preparedness scores. Equity/access: NR.	Mechanism/contribution: Dyad-support mechanism combines pre-discharge planning, rapid home-care nursing and specialist caregiver calls to support SNF-to-home transition. Evidence stratum: Companion/secondary report; Connect-Home ADRD feasibility cohort.
**Study:** Kovaleva et al., 2023 [53] **Location/Country:** USA	**Aim:** Formatively evaluate Tele-Savvy after hospitalization and gather caregiver design feedback **Study design:** Qualitative study	**Population:** N = 15 caregivers completed interviews after Tele-Savvy; caregiver mean age 56.4 years (SD 11.5), female 60%, Black 33%, White 67%, employed 80%; PLWD mean age 82.3 years; median hospital LOS 6 days.	**Discharge/transition context:** Post-hospitalization caregiver support after hospitalization for falls, confusion/altered mental status, behaviour/aggression, COVID-19 or other acute illness.	**Care model/transition component:** 43-day online psychoeducational Tele-Savvy caregiver program used as a prototype for transitional-care intervention development.	**Outcome measures:** Acceptability, videoconference participation, caregiver experience and recommendations for post-discharge intervention design.	**Main findings:** Of 22 consented caregivers, 20 started and 15 completed the intervention; attrition after consent was 32%. Average videoconference attendance was 70% (range 46–96%). Four categories emerged: Improved dementia/caregiving understanding, hospitalization as a new level of normal, PLWD health concerns, and transitional-care intervention development.	Caregiver: Feedback emphasized timing, immediate recovery needs, and support for working caregivers. Implementation: Scheduling and technology accessibility required adaptation. Equity/access: Technology access and working caregiver constraints were identified.	Mechanism/contribution: Tele-education design mechanism tests caregiver acceptability of an online psychoeducational program after hospitalization and defines content/timing needs for dementia transitional care. Evidence stratum: Core transition evidence.
**Study:** Du Preez et al., 2023 [54] **Location/Country:** Australia	**Aim:** Identify carer needs after hospital/ED discharge using CANDID within CELPI **Study design:** Pre-planned qualitative analysis	**Population:** N = 30 carers of people with advanced dementia; carers: 10 spouses, 11 daughters, 6 sons, 3 other relatives and 1 close friend; age 52–88 years; care recipients had advanced dementia (MMSE < 13 or MoCA < 16; FAST 6d-7f).	**Discharge/transition context:** ED/hospital contact and discharge planning in advanced dementia with expected life expectancy < 12 months; care recipients lived at home (*n* = 8) or RACF (*n* = 22).	**Care model/transition component:** CANDID 33-item open-ended needs assessment covering symptoms, emergency contacts, ACP, future needs, carer needs and proposed care plan; referral to specialist palliative care when appropriate.	**Outcome measures:** Carer stressors, systemic barriers, future planning, palliative care needs and end-of-life planning.	**Main findings:** Three major themes were identified: Carers perceived stressors, systemic barriers to care provision, and future planning.	Caregiver: Needs included caregiver health, grief, guilt, multiple roles, family conflict, and uncertainty. Implementation: System navigation and end-of-life planning were difficult. Equity/access: Financial burden was explicitly reported.	Mechanism/contribution: Needs-assessment and palliative mechanism uses CANDID to reveal carer stressors, service barriers and future/end-of-life planning needs after ED or hospital discharge. Evidence stratum: Core transition evidence; palliative-transition emphasis.
**Study:** Saragosa et al., 2023 [55] **Location/Country:** Canada	**Aim:** Develop a grounded theory of hospital-to-home transition in dementia **Study design:** Constructivist grounded theory study	**Population:** N = 25 participants in 21 interviews: 4 people living with dementia and 21 caregivers; persons with dementia mean age 80.75 years; caregivers female 16/21, mean age 63.67 years, caregiving role mean 7.66 years.	**Discharge/transition context:** Hospital-to-home transition and subsequent caregiving trajectory.	**Care model/transition component:** No intervention; theory-building from semi-structured interviews and field notes conducted 2019–2021.	**Outcome measures:** Qualitative processes of dyad work, caregiver navigation, health/social care coordination and transition meaning.	**Main findings:** Generated the theory of “Seeing the day-to-day situation”, with six linked processes: Sealing the situation, working hard, taking charge, taking over, connecting dots and rising up.	Caregiver: Caregivers acted as observers, advocates, coordinators, and constant information holders. Implementation: Support came from public services, family, and private help. Equity/access: Reliance on different support sources may reflect unequal access; no formal subgroup analysis.	Mechanism/contribution: Caregiver-navigation mechanism theorizes caregiver work of observing, taking charge, coordinating care and connecting fragmented information across hospital-to-home transitions. Evidence stratum: Core transition evidence.
**Study:** Temkin-Greener et al., 2023 [56] **Location/Country:** USA	**Aim:** Assess post-acute care transitions and outcomes in ADRD by race/ethnicity and dual status **Study design:** Retrospective Medicare FFS cohort	**Population:** N = 619,262 community-residing Medicare FFS beneficiaries with ADRD and a 2017 hospital stay in 4044 hospitals; White 83.49%, Black 10.26%, Hispanic 6.25%; dual eligible 20.04%; female 59.75%.	**Discharge/transition context:** Hospital discharge to SNF, home health care, or home without services.	**Care model/transition component:** Claims-based comparison of PAC pathways by race/ethnicity, dual Medicare–Medicaid status and hospital/market factors.	**Outcome measures:** PAC destination; 30-day readmission; 30-day mortality.	**Main findings:** Overall discharge destinations were SNF 48.70%, HHC 19.12% and home without services 32.19%. Duals were more often discharged to SNF than non-duals (58.42% vs. 46.26%). Unadjusted 30-day readmission was 18.66% and 30-day mortality 4.77%.	Caregiver: NR as a distinct outcome. Implementation: Claims data mapped post-acute transition pathways. Equity/access: Differences were analysed by race/ethnicity and dual eligibility; dual-status differences were largest.	Mechanism/contribution: PAC-pathway mechanism maps how SNF, home-health and home-without-services pathways and dual-status disparities shape ADRD post-acute outcomes. Evidence stratum: Return-home/community re-entry evidence.
**Study:** Boltz et al., 2023 [57] **Location/Country:** USA	**Aim:** Test Fam-FFC in hospitalized persons with dementia and family care partners **Study design:** Cluster randomized controlled trial	**Population:** N = 455 dyads in final analysis from 6 medical units at 3 hospitals; patients mean age 81.5 years, female 59.1%, White 63.7%, Black 36.3%, mean LOS 6.5 days; FCPs mean age 61.9 years and female 72.3%.	**Discharge/transition context:** Hospitalization and post-acute follow-up at discharge, 2 months and 6 months.	**Care model/transition component:** Fam-FFC delivered by nurse interventionists: Family engagement in assessment, goal setting and care planning; follow-up within 48 h of discharge, weekly calls for 7 weeks, then monthly for 4 months; mean 9.2 contacts/FCP.	**Outcome measures:** Return to baseline function, physical activity, delirium, depressive symptoms, behavioural symptoms, caregiver preparedness, anxiety, strain and burden.	**Main findings:** By 6 months, Fam-FFC participants were more likely to return to baseline function (OR 2.4; 95% CI 1.2–4.7; *p* = 0.01) and had fewer behavioural symptoms of distress (b = −1.1; *p* = 0.05). No group differences were found for moderate physical activity, depressive symptoms or delirium severity.	Caregiver: Preparedness improved from 2 to 6 months; anxiety, strain, and burden did not differ. Implementation: Attrition was 26%, mostly because of death. Equity/access: Black and White participants were represented, but smaller groups were not analysed separately.	Mechanism/contribution: Family-centred function mechanism maps family partnership, bedside functional goals and extended post-acute calls as a model for functional recovery and caregiver preparedness after hospitalization. Evidence stratum: Companion/secondary report; definitive Fam-FFC trial cohort.
**Study:** Berish et al., 2024 [58] **Location/Country:** USA	**Aim:** Examine BPSD prevalence during acute hospitalization and whether BPSD severity/change predicted adverse outcomes **Study design:** Secondary analysis of Fam-FFC data	**Population:** N = 461 patient–care partner dyads from 6 acute medical/surgical units in 3 Pennsylvania hospitals; patients mean age 81.56 years, female 59%, White 63%, mean LOS 6.48 days, mean MoCA 11.63; care partners mean age 61.81 years, female 72%.	**Discharge/transition context:** Acute hospitalization with outcomes at discharge, 2 months and 6 months post-discharge.	**Care model/transition component:** NPI-Q total plus frontal, hyperactivity, mood and psychosis clusters assessed at admission and discharge; post-discharge adverse-event follow-up.	**Outcome measures:** BPSD severity/change; falls, ER visits, hospitalizations and injuries at discharge, 2 months and 6 months.	**Main findings:** BPSD prevalence was 93.9% at admission and 86.7% at discharge. Admission clusters were hyperactivity 76.7%, mood 72.3%, psychosis 71.9% and frontal 25.9%. At 2 months, ER visits occurred in 25.6%, hospitalizations in 22.3%, falls in 21.0% and injuries in 9.3%; at 6 months, ER visits were 27.4%, falls 24.5%, hospitalizations 21.2% and injuries 11.8%.	Caregiver: Companion Fam-FFC report; no separate caregiver outcome was analysed. Implementation: Behavioural-symptom predictors of emergency use and rehospitalization were modelled. Equity/access: Race and education were covariates rather than prespecified equity outcomes.	Mechanism/contribution: Behavioural-risk mechanism maps inpatient BPSD, especially hyperactivity and psychosis change, as post-discharge risk markers for ER use and rehospitalization. Evidence stratum: Companion/secondary report; Fam-FFC-related secondary analysis.
**Study:** Drazich et al., 2025 [59] **Location/Country:** USA	**Aim:** Test the association between caregiver-reported physical resilience at 1 month post-discharge and falls, hospitalizations and death within 1 year **Study design:** Cohort study using FFC-AC-EIT parent-study data	**Population:** N = 314 recently discharged older adults with dementia; mean age 82.74 years (SD 8.13); female 62.4%; White 71.3%, Black/African American 24.5%; mean SLUMS 8.29, Barthel 50.67 and Physical Resilience Scale 11.82/17.	**Discharge/transition context:** One-month post-discharge resilience assessment after acute hospitalization; adverse-event follow-up at 1, 6 and 12 months.	**Care model/transition component:** No discharge intervention tested in this analysis; parent FFC-AC-EIT focused on function-focused care during hospitalization.	**Outcome measures:** Physical Resilience Scale; fall count, hospitalization count and mortality within 1 year.	**Main findings:** Within 1 year, 141/314 (44.9%) had at least one fall, 141/314 (44.9%) were hospitalized and 118/314 (37.6%) died. Higher resilience predicted lower falls (IRR 0.939; 95% CI 0.905–0.974; *p* < 0.001), lower hospitalizations (IRR 0.959; 95% CI 0.925–0.994; *p* = 0.023) and lower mortality odds (OR 0.904; 95% CI 0.865–0.944; *p* < 0.001).	Caregiver: Physical resilience and adverse events were caregiver-reported. Implementation: Clinical and pragmatic significance requires further validation. Equity/access: Financial strain and broader social determinants were not measured.	Mechanism/contribution: Resilience-risk mechanism identifies low early post-discharge physical resilience as a potential profile for targeted fall, rehospitalization and mortality-risk monitoring. Evidence stratum: Return-home/community re-entry evidence.
**Study:** Horstman et al., 2024 [60] **Location/Country:** Houston, TX, USA	**Aim:** Identify caregiver needs during hospitalization and care transitions **Study design:** Pragmatic qualitative inquiry	**Population:** N = 12 family caregivers and 15 health professionals. Caregivers: Family member *n* = 11, friend *n* = 1; mean age 69 years; interviewed mean 18 days after discharge (range 14–34). Professionals: Hospitalists *n* = 4, nurse case managers *n* = 2, social workers *n* = 4, outpatient geriatrics *n* = 2, PCP *n* = 1 and geriatric psychiatrists *n* = 2.	**Discharge/transition context:** Hospitalization and hospital-to-home transition in a Veterans Affairs medical centre.	**Care model/transition component:** No intervention; semi-structured interviews used to inform caregiver transition-support intervention development.	**Outcome measures:** Caregiver information/resource needs; professional-perceived barriers; dementia-specific transition-support recommendations.	**Main findings:** Four recommendations emerged: Engage caregivers as care-team partners; provide dementia-specific information/training; connect caregivers to home/community-based services; provide posthospital care navigation and caregiver support.	Caregiver: Caregivers reported poor clinician communication, limited dementia-specific guidance, and uncertainty about services. Implementation: A dementia navigator or single point of contact was recommended. Equity/access: NR.	Mechanism/contribution: Caregiver-navigation mechanism identifies caregiver partnership, dementia training, home/community service linkage and a dementia navigator as core transition supports. Evidence stratum: Core transition evidence.
**Study:** Knox et al., 2024 [61] **Location/Country:** USA	**Aim:** Examine dementia severity and successful discharge to community after home health **Study design:** Retrospective Medicare/OASIS cohort	**Population:** N = 142,376 Medicare beneficiaries with ADRD receiving home health; 64.3% aged ≥81 years; 60.8% female.	**Discharge/transition context:** Home-health discharge to community with 30-day post-discharge outcome window.	**Care model/transition component:** FAST/OASIS dementia-severity categorization plus caregiver assistance and oral-medication management variables.	**Outcome measures:** Successful discharge to community: Community discharge without unplanned hospitalization or death within 30 days.	**Main findings:** Successful DTC occurred in 71.2%. Advanced dementia stages had lower likelihood of successful DTC: Category 6 RR 0.90 and category 7 RR 0.737 compared with category 2.	Caregiver: Availability, competence, and medication assistance influenced discharge-to-community probability. Implementation: Routine assessment may identify patients needing longer support. Equity/access: NR.	Mechanism/contribution: DTC risk-stratification mechanism connects dementia severity, medication management and caregiver assistance with successful discharge to community from home health. Evidence stratum: Companion/secondary report; Knox Medicare/OASIS home-health series.
**Study:** Paudel et al., 2024 [62] **Location/Country:** USA	**Aim:** Examine family care partner preparedness and associated care-partner characteristics after hospitalization **Study design:** Secondary analysis of Fam-FFC trial data	**Population:** N = 461 family care partners; mean age 61.81 years (SD 14.19); female 72.5%; White 63.9%, Black 33.9%; daughters 37.3%, sons 14.8%, spouses 28.6%; cohabiting 59.4%.	**Discharge/transition context:** Caregiver preparedness measured within 72 h of discharge, at 2 months and at 6 months post-discharge.	**Care model/transition component:** Preparedness analysis within Fam-FFC trial follow-up using care-partner self-report and psychological burden/strain measures.	**Outcome measures:** Preparedness for Caregiving Scale; burden (ZBI-12), strain (MCSI), anxiety/depression (HADS), care-partner demographics and health.	**Main findings:** Preparedness increased from discharge 23.75 (SD 6.78) to 2 months 24.55 (SD 6.49) and 6 months 26.35 (SD 6.72). Lower preparedness was associated with older age at discharge (beta = −0.071; *p* < 0.001), burden at all timepoints (*p* < 0.001, *p* < 0.001 and *p* = 0.002/0.003), depression at all timepoints (*p* = 0.002/0.041/0.003) and strain at 2 and 6 months (*p* = 0.015/0.025).	Caregiver: Caregiver psychological state and burden were transition-support targets. Implementation: Support may be particularly important after the initial discharge period. Equity/access: NR.	Mechanism/contribution: Preparedness mechanism maps care-partner age, burden, depression and strain as determinants of discharge-to-6-month caregiving readiness. Evidence stratum: Companion/secondary report; Fam-FFC secondary analysis.
**Study:** Toles et al., 2024 [63] **Location/Country:** USA	**Aim:** Describe transitional care content and quality in the ADRD-PC trial **Study design:** Mixed-methods study	**Population:** N = 40 hospitalized late-stage ADRD–caregiver dyads receiving transitional palliative care support, plus palliative care staff interviews (N = 8); parent trial includes GDS 5–7 dementia.	**Discharge/transition context:** Hospital discharge with specialty palliative care transitional support.	**Care model/transition component:** Standardized palliative care plan plus post-discharge caregiver calls at 72 h and 2 weeks; interdisciplinary palliative care consultation, caregiver education and community referral planning.	**Outcome measures:** Palliative care plan content, post-discharge call completion, symptoms, resources, caregiver needs, implementation barriers and facilitators.	**Main findings:** Palliative care plans were documented for 29/40 dyads (73%) and delivered to post-acute providers for 26/40 (65%). Calls were completed at 72 h for 39/40 (98%) and at 2 weeks for 33/40 (78%). Calls addressed continuity, resources, active listening, troubleshooting and grief/loss support.	Caregiver: Facilitators included predischarge contact, prognosis understanding, and a written palliative plan. Implementation: Barriers included short referral windows, discordant understanding, transfer ambiguity, and scarce resources. Equity/access: Scarce community resources limited implementation.	Mechanism/contribution: Palliative-transition mechanism pulls hospital palliative care plans into post-discharge calls to support continuity, troubleshooting, resources and grief support. Evidence stratum: Core transition evidence; ADRD-PC program report.
**Study:** Toles et al., 2024 [64] **Location/Country:** USA	**Aim:** Describe health trajectories after SNF discharge in ADRD **Study design:** Secondary analysis/multiple case study from Connect-Home trial data	**Population:** N = 49 SNF patients with ADRD from a 327-dyad parent study; mean age 81.2 years; female 65.3%; White 73.5%, Black 26.5%; caregivers mean age 62.2 years, female 77.6%.	**Discharge/transition context:** SNF discharge to patient home, family home or assisted living; 30-day follow-up.	**Care model/transition component:** Multiple case-study analysis using baseline data, chart data and caregiver-reported outcomes from a transitional-care trial.	**Outcome measures:** Acute care use, hospital readmission, death, discharge destination and trajectory type within 30 days.	**Main findings:** Among 41 dyads with complete outcome data, 68.3% were stable at 30 days, 24.4% had acute care use and 7.3% died. Ten patients were readmitted, including four for urinary tract infection, two for acute renal failure and two for hip fracture.	Caregiver: Caregiver sex and employment were not consistently associated with readmission. Implementation: Clinical factors supported readmission-risk stratification. Equity/access: NR.	Mechanism/contribution: Health-trajectory mechanism maps post-SNF ADRD trajectories and recurrent acute illness/injury to guide dyad-centred nursing support. Evidence stratum: Companion/secondary report; Connect-Home trial secondary analysis.
**Study:** Yang et al., 2024 [65] **Location/Country:** USA	**Aim:** Examine racial/ethnic and dual-eligibility disparities in discharge to below-average quality HHAs before and during COVID-19 **Study design:** Retrospective Medicare cohort	**Population:** N = 426,766 qualified hospitalization events among community-dwelling Medicare FFS beneficiaries with ADRD discharged to HHA; pre-COVID *n* = 205,850 and COVID-period *n* = 220,916.	**Discharge/transition context:** Hospital discharge to HHA within 3 days after non-COVID acute hospitalization, January 2019–November 2021.	**Care model/transition component:** Linkage of Medicare claims with HHA Quality of Patient Care Star Ratings, Area Deprivation Index and county COVID data; below-average HHA defined as average star rating <3.0.	**Outcome measures:** Discharge to below-average quality HHA; race/ethnicity; Medicare–Medicaid dual eligibility; pre-COVID vs. COVID period; sensitivity definitions including ≤2.5 stars and Patient Survey Star Ratings.	**Main findings:** Before COVID-19, 18.8% were discharged to below-average quality HHAs. Black and Hispanic beneficiaries had higher probabilities than White beneficiaries (3.4 and 3.9 percentage points; *p* < 0.01), and dual-eligible beneficiaries were 2.5 percentage points higher (*p* < 0.01). During COVID, racial disparities persisted; dual disparities increased by 0.8 percentage points (*p* < 0.05).	Caregiver: NR as a distinct outcome. Implementation: Home-health agency quality was the dominant implementation exposure. Equity/access: Black, Hispanic, and dual-eligible patients were overrepresented in below-average-quality agencies.	Mechanism/contribution: Equity/access mechanism maps how race/ethnicity and dual eligibility shape access to HHA quality after ADRD hospitalization. Evidence stratum: Return-home/community re-entry evidence; equity-focused transition evidence.
**Study:** Greyson et al., 2025 [66] **Location/Country:** USA	**Aim:** Explore role ambiguity and perceived care quality during hospital-to-home transitions of older Latinos with dementia **Study design:** Qualitative study guided by the Systems Ambiguity Framework	**Population:** N = 21 caregivers of 22 older Latinos with ADRD; caregivers mean age 53 years, female 71%, Hispanic/Latino 95%; child caregivers 71%, one of several family caregivers 57%, long-distance 24%; older adults mean age 75 years, female 72%, moderate/severe CDR 91%.	**Discharge/transition context:** Hospital-to-home transition within the previous 12 months; two interviews per caregiver approximately 4 weeks apart, conducted in Spanish or English.	**Care model/transition component:** No intervention; interviews on medication management, role/task ambiguity, institutional trust, cultural expectations and perceived care quality.	**Outcome measures:** Medication-management concerns; role/task ambiguity; trust; culturally specific resource needs; factors shaping task distribution.	**Main findings:** Themes included concern about over-medication, resignation about limited ADRD medication effectiveness, scarcity of culturally specific/translated resources, wariness to trust institutions and aversion to institutional care. Task allocation was shaped by geographic proximity, gender roles, relationship, English fluency and work schedule.	Caregiver: Cultural factors shaped who became caregiver; medication and navigation responsibilities remained ambiguous. Implementation: Generic resources were viewed as inadequate. Equity/access: Latino caregivers requested trusted, culturally tailored home support.	Mechanism/contribution: Cultural-tailoring mechanism links task ambiguity, medication concerns, language/cultural barriers and institutional mistrust to transition quality in Latino ADRD dyads. Evidence stratum: Core transition evidence; equity-focused qualitative evidence.
**Study:** Idso et al., 2025 [67] **Location/Country:** Minneapolis, MN, USA	**Aim:** Evaluate feasibility and acceptability of caregiver-focused nurse-led post-discharge calls for veterans with dementia **Study design:** Single-site VA quality-improvement project	**Population:** 103 charts assessed; 73 excluded after chart review; 30 caregivers recruited; 19 completed calls and 16 completed follow-up. Veterans were male, mean age 79 years and mean LOS 4 days; caregivers female 94.7%, spouse/partner 78.9% and lived with the veteran 84.2%.	**Discharge/transition context:** VA acute-care discharge to private residence or independent living; calls completed mean 17 days post-hospitalization (SD 8).	**Care model/transition component:** Registered-nurse call using caregiver-preparedness items and age-friendly 4M domains: What matters, mentation, mobility and medications; tailored resources mailed; nursing note placed in EHR.	**Outcome measures:** Feasibility of caregiver identification/contact; call duration; Preparedness for Caregiving Scale; satisfaction and qualitative acceptability.	**Main findings:** Mean call duration was 29 min. Overall, 36.8% rated themselves very well prepared to care for the veteran; 47.4% for physical needs, 31.6% for emotional needs, 26.3% for behavioural symptoms, 31.6% for setting up services, and 42.1% for emergencies/help from the health system. 21.1% were not prepared at all to find/set up services.	Caregiver: Caregiver identification was resource-intensive and required review of more than 100 charts to recruit 30 participants. Implementation: Acceptability supported tailored content and flexible timing. Equity/access: NR.	Mechanism/contribution: Pragmatic call mechanism tests caregiver-centred nurse calls and shows that EHR caregiver identification is a key implementation bottleneck. Evidence stratum: Core transition evidence.

Abbreviations: ACP = advance care planning; AD = Alzheimer’s disease; ADRD = Alzheimer’s disease and related dementias; ADL = activities of daily living; ADI = Area Deprivation Index; ALF = assisted living facility; BPSD = behavioural and psychological symptoms of dementia; CANDID = Carer Needs Directed Assessment in Dementia; CDR = Clinical Dementia Rating; CELPI = Carer End of Life Planning Intervention; CTM-15 = Care Transitions Measure-15; DAA = Dose administration aid; DTC = discharge to community; ED = emergency department; ER = emergency room; FAST = Functional Assessment Staging Tool; FCG = family caregiver; FCP = family care partner; FFS = fee-for-service; GDS = Global Deterioration Scale; GP = general practitioner; HHA = home health agency; HHC = home health care; HMR = home medicines review; LOS = length of stay; MCSI = Modified Caregiver Strain Index; MNA = Mini Nutritional Assessment; MoCA = Montreal Cognitive Assessment; M-SDC = modified successful discharge to community; NH = nursing home; NPI-Q = Neuropsychiatric Inventory Questionnaire; NR = not reported in the available PDF/extraction; OASIS = Outcome and Assessment Information Set; PAC = post-acute care; PCH = personal care home; PCP = primary care physician; PCS = Preparedness for Caregiving Scale; PPR = potentially preventable readmission; PT/OT/ST = physical/occupational/speech therapy; PWD = person with dementia; RACF = residential aged care facility; SACU = Special Alzheimer acute care unit; SLUMS = Saint Louis University Mental Status exam; SNF = skilled nursing facility. Evidence categories describe transition proximity or reporting overlap, not methodological quality.

**Table 2:** **Rehabilitation continuity, return-home support and post-discharge functional follow-up in Parkinson’s disease and multiple sclerosis.** This table summarizes studies in which inpatient rehabilitation, multidisciplinary rehabilitation, multimodal complex treatment, home-based follow-up, telecoaching, telerehabilitation, self-management, or functional carry-over after discharge represented the dominant post-discharge continuity component.

Author, Year [Ref.]/Location/Country	Aim and Study Design	Disease Group and Population Characteristics	Rehabilitation or Discharge Anchor	Post-Discharge Continuity Component	Comparator and Follow-Up Duration	Outcome Measures	Main Findings	Key Transition Mechanism and Contribution to the Evidence Map
**Study:** Freeman et al., 1999 [21] **Location/Country:** United Kingdom	**Aim:** Determine the duration and pattern of carry-over of benefits after short inpatient multidisciplinary rehabilitation **Study design:** Prospective single-group longitudinal study	**Disease group:** Multiple sclerosis. **Population:** N = 50 progressive MS; female 58%; mean age 44.8 ± 9.7 years; secondary progressive 86%, primary progressive 14%; median EDSS 6.8 (range 6.0–9.0).	**Rehabilitation/discharge anchor:** Individualized, goal-oriented inpatient MDR; mean stay 23 days (SD 11.5; range 10–62); assessments at admission, discharge and every 3 months to 12 months.	**Post-discharge continuity component:** Discharge planning and community-service linkage; 48/50 discharged home. Recommended services included wheelchair services (*n* = 27), community OT (*n* = 21), district nursing (*n* = 14), outpatient physiotherapy (*n* = 12) and house adaptations (*n* = 30).	**Comparator:** No control group **Follow-up duration:** 12-month post-discharge follow-up; 12-month data collected for 92% of patients.	**Outcome measures:** EDSS, FIM motor, London Handicap Scale, SF-36, GHQ; time to return to baseline.	**Main findings:** Neurological status worsened (median EDSS 6.8 at admission to 8.0 at 12 months), but rehabilitation gains were partly retained: Disability 186 days, handicap 185 days, emotional well-being 208 days and SF-36 physical component 293 days before return to baseline.	Mechanism/contribution: Quantifies how benefits from inpatient MS rehabilitation persist after community return despite neurological progression, and highlights the need for formal links between inpatient and community services. Evidence stratum: Return-home/community re-entry evidence.
**Study:** Pozzilli et al., 2002 [22] **Location/Country:** Rome, Italy	**Aim:** Compare effectiveness and costs of multidisciplinary home-based MS care versus hospital care over 1 year **Study design:** Randomized controlled trial with economic evaluation	**Disease group:** Multiple sclerosis. **Population:** N = 201 clinically definite MS; home-care *n* = 133 and hospital-care *n* = 68; intervention group mean age 47 ± 10.3 years, women 65%, disease duration 18.4 ± 9.5 years, EDSS 6.0 ± 2.0; RR 19.6%, PP 20.5%, SP 59.9%.	**Rehabilitation/discharge anchor:** Home-based management as an alternative to hospital-based MS care.	**Post-discharge continuity component:** Interdisciplinary home-care team with neurologists, urologist, rehabilitation physician, psychologist, physical therapist, nurse, social worker and coordinator; home visits, telephone follow-up and weekday dedicated phone line.	**Comparator:** Hospital-care comparator **Follow-up duration:** 12-month follow-up; complete/partial follow-up data in *n* = 123 home-care and *n* = 65 control patients.	**Outcome measures:** EDSS, FIM, MMSE, FSS, STAI/STAXI, depression, SF-36, resource use and direct health-care costs.	**Main findings:** No between-group differences in functional status. Home care favoured SF-36 bodily pain, general health, social functioning and role-emotional domains (*p* ≤ 0.001), as well as PCS and MCS (*p* = 0.0001). Direct health-care cost was €822/patient/year lower with home care, mainly through fewer hospital admissions.	Mechanism/contribution: Home-management mechanism maps interdisciplinary home follow-up as a community-management alternative that preserved functional status, improved selected HRQOL domains and reduced health-care costs. Evidence stratum: Return-home/community re-entry evidence.
**Study:** Frazzitta et al., 2012 [68] **Location/Country:** Italy	**Aim:** Assess whether intensive inpatient rehabilitation improves motor performance over 12 months, whether a second cycle has similar efficacy, and whether levodopa escalation is reduced **Study design:** Randomized controlled trial	**Disease group:** Parkinson’s disease. **Population:** N = 50 Hoehn–Yahr stage 3 PD; IRT *n* = 25 and control *n* = 25. Treated patients: Age 72 ± 7 years, M/F 11/14, disease duration 8 ± 3 years. Controls: Age 70 ± 7 years, M/F 13/12, duration 9 ± 3 years.	**Rehabilitation/discharge anchor:** Four-week intensive inpatient rehabilitation treatment; 3 daily 1 h sessions, 5 days/week, including stretching, treadmill plus auditory/visual cues, stabilometric platform and occupational therapy.	**Post-discharge continuity component:** Discharge instructions to continue learned exercises and walk at least 30 min/day; same inpatient cycle repeated after 12 months.	**Comparator:** Usual pharmacological care plus generic home exercise advice **Follow-up duration:** 1-year follow-up.	**Outcome measures:** UPDRS II, UPDRS III, UPDRS total; levodopa-equivalent dosage.	**Main findings:** At 1 year, treated patients had UPDRS values similar to baseline, whereas controls worsened by UPDRS II +4.9, UPDRS III +5.5 and UPDRS total +9.2 (all *p* < 0.0001). The first and second IRT cycles reduced UPDRS total by 11.6 and 9.6 points. Levodopa dosage decreased by 52 mg in treated patients and increased by 30 mg in controls.	Mechanism/contribution: Inpatient-to-home exercise carry-over mechanism links intensive cue-based rehabilitation, home exercise instructions and repeated rehabilitation cycles with 1-year motor stability and reduced medication escalation. Evidence stratum: Return-home/community re-entry evidence.
**Study:** Frazzitta et al., 2013 [69] **Location/Country:** Italy	**Aim:** Evaluate short-term and 1-year effects of 4-week MIRT on balance and gait **Study design:** Preliminary single-group longitudinal study	**Disease group:** Parkinson’s disease. **Population:** N = 20 PD inpatients; 7 males/13 females; age 71 ± 7 years; disease duration 8.3 ± 2.1 years; Hoehn–Yahr stage 3; UPDRS total 34.74, UPDRS III 18.18 and UPDRS II 14.48 at baseline.	**Rehabilitation/discharge anchor:** Four-week inpatient MIRT with 3 daily sessions, 5 days/week, including treadmill with auditory/visual cues, stabilometric platform and occupational therapy.	**Post-discharge continuity component:** Unsupervised home continuation of learned exercises and walking at least 30 min/day; pharmacological therapy unchanged.	**Comparator:** No control group **Follow-up duration:** Assessments at admission, discharge and 1-year follow-up.	**Outcome measures:** UPDRS items for falls, walking and balance; BBS, 6MWT, TUG, comfortable and fast gait speed.	**Main findings:** All outcomes improved at discharge. Examples: 6MWT 258.1 ± 86.1 to 322.7 ± 107.4 m, TUG 12.4 ± 3.0 to 9.6 ± 2.5 s, and BBS 45.1 ± 7.4 to 50.8 ± 6.8 (all *p* < 0.0001). At 1 year, UPDRS walk, comfortable gait speed and fast gait speed remained better than baseline (*p* = 0.009, *p* = 0.03 and *p* = 0.02), while balance outcomes returned toward baseline.	Mechanism/contribution: Gait/balance carry-over mechanism separates short-term gains from partial long-term maintenance, showing stronger 1-year preservation of gait speed than balance. Evidence stratum: Return-home/community re-entry evidence.
**Study:** Boesen et al., 2018 [70] **Location/Country:** Denmark	**Aim:** Evaluate 6-month effects of inpatient multidisciplinary rehabilitation on HRQOL in MS **Study design:** Two-hospital pragmatic randomized controlled trial	**Disease group:** Multiple sclerosis. **Population:** N = 427 randomized; treatment *n* = 214 and wait-list control *n* = 213; ITT *n* = 209 treatment and *n* = 204 control. Eligible patients were aged 18–65 years with MS and EDSS ≤ 7.5.	**Rehabilitation/discharge anchor:** Four-week inpatient MDR at Danish MS Hospitals; 20 scheduled rehabilitation days; median stay 19 days; mean therapy approximately 3.5 h/day.	**Post-discharge continuity component:** Post-discharge HRQOL assessment after individualized MDR; no continuation or repetition of the inpatient MDR program after discharge.	**Comparator:** Wait-list control for 6 months **Follow-up duration:** Community training/services and MS clinic care were not restricted.	**Outcome measures:** FAMS, MSIS-29, EQ-5D-5L, EQ-VAS and 15D.	**Main findings:** Trends favoured MDR across HRQOL measures. Significant 6-month effects were observed for MSIS-29 Psychological (treatment effect −2.7; 95% CI −5.6 to −0.1; *p* = 0.046) and 15D (+0.017; 95% CI 0.005 to 0.030; *p* = 0.008). FAMS was not statistically significant.	Mechanism/contribution: MDR HRQOL mechanism maps short-term community carry-over of inpatient MS rehabilitation, with stronger signals for psychological and generic HRQOL domains than for the prespecified FAMS endpoint. Evidence stratum: Companion/secondary report.
**Study:** Ferrazzoli et al., 2018 [71] **Location/Country:** Italy	**Aim:** Evaluate whether 4-week MIRT improves PD-related quality of life in the short and medium term **Study design:** Prospective parallel-group single-blind randomized controlled study	**Disease group:** Parkinson’s disease. **Population:** N = 234 analysed: MIRT *n* = 186 and control *n* = 48; idiopathic PD, Hoehn–Yahr 2–4. MIRT group: Age 66.5 ± 8.6 years, male 57.0%, disease duration 9.0 ± 5.6 years, H&Y 2.6 ± 0.5. Controls: Age 66.9 ± 10.5 years, male 62.5%.	**Rehabilitation/discharge anchor:** Four-week inpatient MIRT with multidisciplinary, aerobic, motor-cognitive and intensive sessions; four daily rehabilitative sessions for 5 days/week plus 1 h exercise on day 6.	**Post-discharge continuity component:** QoL assessed after return from hospitalization to avoid immediate hospital-context bias; intervention group also followed to 18 weeks from enrolment.	**Comparator:** No-rehabilitation/wait-list control **Follow-up duration:** Controlled T1 at 10 weeks from enrolment and T2 within-MIRT follow-up at 18 weeks.	**Outcome measures:** PDQ-39 global index and dimensions; UPDRS, PDDS, TUG, BBS and LEDD.	**Main findings:** PDQ-39 global index improved in MIRT from 43.6 ± 21.4 to 35.3 ± 22.1 (delta −8.3 ± 18.0; *p* < 0.0001), while controls were unchanged (41.6 ± 22.9 to 42.8 ± 22.9; delta +1.2 ± 9.9; *p* = 0.23); between-group delta *p* < 0.0001. At 18 weeks, MIRT retained improvement (delta −4.8 ± 17.5; *p* < 0.0001).	Mechanism/contribution: QoL carry-over mechanism shows that intensive inpatient PD rehabilitation can produce PDQ-39 gains that remain detectable after discharge, while emphasizing cautious interpretation because of single-blind design and possible contextual effects. Evidence stratum: Return-home/community re-entry evidence.
**Study:** Isernia et al., 2020 [72] **Location/Country:** Italy	**Aim:** Test the efficiency and clinical effects of the HEAD clinic-to-home telerehabilitation program on QoL, motor and non-motor abilities **Study design:** Multicentre pilot clinical trial	**Disease group:** Parkinson’s disease. **Population:** N = 31 PD outpatients; mean age 66.84 ± 9.13 years; M/F 17/14. After ClinicHEAD, HomeHEAD *n* = 11 and usual care *n* = 20; groups were similar at baseline.	**Rehabilitation/discharge anchor:** Clinic-based VR/digital rehabilitation: 12 ClinicHEAD sessions, 45 min, 3 sessions/week.	**Post-discharge continuity component:** HomeHEAD telerehabilitation: 60 sessions, 45 min, 5 sessions/week over 3 months, delivered through a digital health platform and gaming devices; remote monitoring and help-desk support.	**Comparator:** Usual care after clinic phase **Follow-up duration:** Follow-up at T2 and T3, up to 7 months from baseline.	**Outcome measures:** 2MWT, BBS, 10MWT, Box and Block Test, MoCA, RBMT-GMI, SF-12/PANAS, adherence and SUS usability.	**Main findings:** ClinicHEAD improved 2MWT from 131.23 ± 36.72 to 140.30 ± 37.54 m (*p* = 0.024), MoCA from 21.94 ± 2.82 to 22.88 ± 3.51 (*p* = 0.032), and BBT dominant hand from 41.48 ± 13.56 to 46.39 ± 13.73 (*p* < 0.001). High adherence was 83.9% in clinic and 72.7% at home; SUS median was 70 at T1 and 85 at T2. HomeHEAD further improved non-dominant BBT (*p* = 0.004; d = 0.86) and better maintained balance/function at T3 than usual care.	Mechanism/contribution: Clinic-to-home telerehabilitation mechanism bridges supervised digital rehabilitation with home VR practice, supporting adherence, usability and maintenance of functional mobility. Evidence stratum: Core transition evidence.
**Study:** Boesen et al., 2020 [73] **Location/Country:** Denmark	**Aim:** Investigate long-term HRQOL effects of inpatient multidisciplinary rehabilitation in MS at 12 months **Study design:** Randomized controlled partial crossover trial report	**Disease group:** Multiple sclerosis. **Population:** N = 413 analysed from the Danish MS Hospitals cohort; Group A *n* = 209 and Group B *n* = 204; median age 51–52 years, female 68–69%, median EDSS 4.5–5.0; 402 patients admitted.	**Rehabilitation/discharge anchor:** Four-week inpatient MDR; median length of stay 20 days (range 7–20).	**Post-discharge continuity component:** Long-term follow-up after MDR using partial crossover comparison of treatment and control periods.	**Comparator:** Partial crossover/wait-list sequence **Follow-up duration:** 6- and 12-month follow-up.	**Outcome measures:** FAMS, MSIS-29 physical/psychological, 15D, EQ index, EQ-VAS, EDSS and immunotherapy use.	**Main findings:** Statistically significant 12-month improvements were found in 3 of 6 HRQOL measures: FAMS, 15D and EQ-VAS. FAMS mean difference was +3.3 (95% CI 0.7 to 5.9; *p* = 0.018) at 6 months and +4.7 (95% CI 2.1 to 7.1; *p* < 0.001) at 12 months. Three of four suggested MCID thresholds were unmet; no overall EDSS progression occurred.	Mechanism/contribution: Long-term MDR carry-over mechanism supports lasting but clinically modest HRQOL gains after inpatient MS rehabilitation and frames long-term benefit as domain-specific rather than uniformly clinically significant. Evidence stratum: Companion/secondary report.
**Study:** Feldmann et al., 2020 [74] **Location/Country:** Germany	**Aim:** Describe PD medication changes 1 month after hospital discharge and examine associations with self-reported adherence **Study design:** Observational post-discharge study	**Disease group:** Parkinson’s disease. **Population:** N = 125 hospitalized people with PD; mean age 70.0 ± 8.0 years; male 60.8%; median H&Y 3.0; disease duration 8.1 ± 5.2 years; MDS-UPDRS III 24.3 ± 11.2; LEDD 602.2 ± 346.7 mg; MoCA 23.5 ± 2.9.	**Rehabilitation/discharge anchor:** Neurological hospitalization for PD-related worsening, fluctuations, dyskinesias, off-phases, gait/freezing or related reasons.	**Post-discharge continuity component:** Semi-structured telephone interview 4 weeks after discharge assessing PD medication changes, initiator and reasons.	**Comparator:** No comparator **Follow-up duration:** 1-month post-discharge follow-up.	**Outcome measures:** PD medication changes, SAMS adherence, MDS-UPDRS III, LEDD, NMSQ, MoCA and BDI-II.	**Main findings:** PD medication changed in 38/125 patients (30.4%), by physicians in 26 (20.8%; neurologists *n* = 17, GPs *n* = 9), and by patients in 12 (9.6%). Eleven changes (8.8%) had been recommended in discharge letters, 27 (21.6%) were unintended. Baseline SAMS predicted patient-initiated medication changes (*p* < 0.001). Common reasons were side effects (*n* = 10) and insufficient effect (*n* = 8).	Mechanism/contribution: Medication-continuity mechanism identifies early post-discharge medication modification and baseline nonadherence as safety targets after PD hospitalization. Evidence stratum: Core transition evidence.
**Study:** Flachenecker et al., 2020 [24] **Location/Country:** Germany	**Aim:** Evaluate whether internet-based PA/exercise promotion maintains fatigue, HRQOL and gait benefits after inpatient rehabilitation **Study design:** Randomized single-blind controlled study	**Disease group:** Multiple sclerosis. **Population:** N = 84 randomized, *n* = 64 analysed; intervention *n* = 34 and control *n* = 30. Intervention group: Age 47.6 ± 9.2 years, women 65%, disease duration 13.4 ± 7.9 years, EDSS median 4.3; control: Age 46.4 ± 12.2 years, women 57%, EDSS median 4.0.	**Rehabilitation/discharge anchor:** Inpatient multimodal rehabilitation for fatigued pwMS; EDSS ≤6.0 and WEIMuS ≥32.	**Post-discharge continuity component:** Three-month internet-based PA/exercise promotion after discharge with web/telephone coaching, individualized exercise prescription and browser-based training diary.	**Comparator:** Control group received no aftercare intervention **Follow-up duration:** Assessments at discharge, 3 months and 6 months.	**Outcome measures:** WEIMuS fatigue, MSIS-29 HRQOL, 2MWT, 10mWT and Tinetti balance.	**Main findings:** Inpatient rehabilitation reduced fatigue in both groups, but aftercare better preserved gains: WEIMuS improvement from baseline was 16.5 vs. 7.0 at month 3 and 22.5 vs. 5.5 at month 6 (both *p* < 0.001, IG vs. CG). MSIS-29 improved after rehabilitation in both groups but remained improved only in the intervention group through 6 months; gait gains were more pronounced in the intervention group at 3 months.	Mechanism/contribution: Digital aftercare mechanism shows that internet-based PA/exercise promotion can sustain fatigue, HRQOL and gait benefits after MS inpatient rehabilitation. Evidence stratum: Core transition evidence.
**Study:** Chen et al., 2021 [75] **Location/Country:** China	**Aim:** Assess short-term MIRT effects across PD motor subtypes and maintenance at 3 months **Study design:** Prospective pilot study	**Disease group:** Parkinson’s disease. **Population:** N = 69 idiopathic PD; PIGD *n* = 36, TD *n* = 19, indeterminate *n* = 14; 34 men/35 women; mean age 60.6 ± 7.0 years; disease duration 6.0 ± 3.2 years; mean H&Y 2.5 ± 0.5; H&Y ≤3.	**Rehabilitation/discharge anchor:** Two-week inpatient MIRT, 5 days/week, four daily sessions, including one-to-one physiotherapy, cue-based treadmill/balance training, aerobic training and speech therapy.	**Post-discharge continuity component:** Remote home rehabilitation health-education platform introduced at discharge; patients asked to sign in after daily home exercises.	**Comparator:** No external control **Follow-up duration:** Discharge assessment and 3-month patient-reported follow-up; follow-up data in *n* = 66.	**Outcome measures:** MDS-UPDRS III, M-PAS, BBS, 10MT, 6MWD, FTSTS, TUG and 3-month patient-reported duration/grade of effect.	**Main findings:** All patients improved after MIRT: MDS-UPDRS III 29.0 ± 10.7 to 24.3 ± 10.4, M-PAS 52 to 54, BBS 54 to 55, 6MWD 465 to 510 m, and TUG 9.6 to 8.5 s (all *p* < 0.001). MDS-UPDRS III change was greater in PIGD than TD after adjustment (−5.6 ± 4.9 vs. −3.5 ± 3.8; *p* = 0.021). Three-month follow-up suggested effect maintenance for 1–3 months, without significant between-subtype differences.	Mechanism/contribution: Subtype-specific carry-over mechanism links short inpatient MIRT and remote home education with short-term motor gains across PD subtypes, while identifying stronger early MDS-UPDRS III response in PIGD. Evidence stratum: Return-home/community re-entry evidence.
**Study:** Kuendig et al., 2022 [76] **Location/Country:** Switzerland	**Aim:** Evaluate whether 3-week inpatient rehabilitation improves daily PA and walking capacity in MS with moderate-to-severe walking disability **Study design:** Exploratory observational study	**Disease group:** Multiple sclerosis. **Population:** N = 24 MS patients; mean age 50.8 ± 11.1 years; female 50%; median MS duration 13.0 years [IQR 4.8–17.0]; median EDSS 6.0 [4.5–6.5]; PPMS *n* = 6, SPMS *n* = 8, RRMS *n* = 10.	**Rehabilitation/discharge anchor:** Three-week inpatient multidisciplinary rehabilitation for moderate-to-severe walking disability.	**Post-discharge continuity component:** Home measurement of physical activity before rehabilitation, 1 week after rehabilitation and 3 months after inclusion; no PA-specific behavioural intervention during rehabilitation.	**Comparator:** No comparator **Follow-up duration:** Before/after rehabilitation and 3-month follow-up.	**Outcome measures:** Accelerometer-based daily PA, 2MWT, TUG, FSMC motor/cognitive fatigue and HADS mood.	**Main findings:** Rehabilitation improved walking capacity (+17 m; +20.2%; ES = 0.74; 95% CI 0.31–1.16) and mobility (TUG −2.1 s; −14.9%; ES = 0.65; 95% CI 0.22–1.07), as well as motor fatigue (ES = 0.56) and cognitive fatigue (ES = 0.44). Daily PA did not improve; at 3 months PA was reduced by a median 30 min/day (*p* = 0.0055).	Mechanism/contribution: Capacity-to-performance gap mechanism demonstrates that improved walking capacity and fatigue after MS rehabilitation may not translate into real-world PA without behavioural aftercare. Evidence stratum: Return-home/community re-entry evidence.
**Study:** Patt et al., 2023 [77] **Location/Country:** Switzerland	**Aim:** Test whether inpatient energy management education plus HIIT is superior to PMR plus moderate continuous training for HRQOL at 6 months **Study design:** Randomized controlled superiority trial	**Disease group:** Multiple sclerosis. **Population:** N = 106 fatigued pwMS; IEME + HIIT *n* = 53 and PMR + MCT *n* = 53; mean age 49.75 ± 9.87 years; female 66%; EDSS 4.64 ± 1.32; FSMC ≥ 43 and EDSS ≤ 6.5.	**Rehabilitation/discharge anchor:** Three-week inpatient multidisciplinary rehabilitation plus twice-weekly education/relaxation and three weekly endurance-training sessions.	**Post-discharge continuity component:** Individual home exercise plan and a 6-week booster letter reminding patients of discharge goals.	**Comparator:** Active comparator: IEME + HIIT vs. PMR + MCT **Follow-up duration:** Assessments at discharge, 4 months and 6 months.	**Outcome measures:** SF-36 PCS/MCS and subscales, FSMC, HADS, SEPECSA, OSA and VO2peak.	**Main findings:** IEME + HIIT was not superior to PMR + MCT for primary SF-36 PCS/MCS at 6 months. Pairwise differences favoured IEME + HIIT for VO2peak (*p* = 0.011) and SEPECSA (*p* = 0.032) at discharge, SF-36 mental health (*p* = 0.022), HADS anxiety (*p* = 0.014) and SEPECSA (*p* = 0.040) at 4 months, and SF-36 physical functioning (*p* = 0.012) and SEPECSA (*p* = 0.003) at 6 months.	Mechanism/contribution: Fatigue self-management mechanism tests whether energy-management education plus HIIT strengthens self-efficacy and HRQOL after inpatient MS rehabilitation; secondary signals support targeted self-management. Evidence stratum: Return-home/community re-entry evidence.
**Study:** Steendam-Oldekamp et al., 2023 [78] **Location/Country:** Netherlands	**Aim:** Assess whether combined inpatient rehabilitation and 2-year outpatient support improves ADL and delays definite nursing-home admission **Study design:** Non-randomized controlled multicentre trial	**Disease group:** Advanced Parkinson’s disease. **Population:** Intervention *n* = 24 and matched control *n* = 19; advanced PD at risk of nursing-home admission. Intervention: Men 54%, median age 70 years, disease duration 8 years, H&Y IV 62.5%/V 37.5%, probable PDD 54.2%, hallucinations 41.7%.	**Rehabilitation/discharge anchor:** Six-week inpatient multidisciplinary rehabilitation and medication optimization at a Parkinson expertise centre.	**Post-discharge continuity component:** Two-year individualized outpatient multidisciplinary support via ParkinsonNet-trained professionals; focus on ADL, medication and home living.	**Comparator:** Matched usual-care/nursing-home controls **Follow-up duration:** 2-year follow-up.	**Outcome measures:** ALDS, independent home living, LEDD, SCOPA-SPES, SCOPA-COG, NPI-Q and BDI.	**Main findings:** After inpatient intervention, 20/24 patients (83.3%) returned home; 13/20 (65%) still lived at home at 2 years. ALDS remained near baseline in the intervention group (59.28 to 62.61; *p* = 0.140) but declined in controls (69.33 to 29.33; *p* = 0.017); between-group *p* = 0.002. LEDD increased by median 495 mg in the intervention group (*p* = 0.002).	Mechanism/contribution: Institutionalization-prevention mechanism combines short inpatient stabilization with 2-year outpatient support to maintain ADL and delay definite nursing-home admission in advanced PD. Evidence stratum: Return-home/community re-entry evidence.
**Study:** Nørgaard et al., 2024 [25] **Location/Country:** Denmark	**Aim:** Investigate whether monthly telephone or web-based telecoaching after inpatient MS rehabilitation enhances long-term HRQOL carry-over **Study design:** Exploratory matched-control study	**Disease group:** Multiple sclerosis. **Population:** N = 40 after inpatient MDR; telephone *n* = 20 and web-based *n* = 20. Neuropsychological goal group: Telephone *n* = 18, web *n* = 12; physical goal group: Telephone *n* = 2, web *n* = 8.	**Rehabilitation/discharge anchor:** Discharge after 4-week inpatient MDR from Danish MS Hospitals.	**Post-discharge continuity component:** Monthly telecoaching for 11 months: Telephone one-to-one coaching or text-only web-based coaching linked to personal rehabilitation goals.	**Comparator:** Matched wait-list and treatment controls from parent trial **Follow-up duration:** 6- and 12-month follow-up.	**Outcome measures:** FAMS HRQOL; coaching adherence and call duration.	**Main findings:** Adherence was 100%; each patient completed 9 sessions. Telephone group had 180 sessions, mean duration 25 min; web group required 33 unplanned calls, mean duration 13 min. At 12 months in the neuropsychological group, FAMS difference vs. wait-list control was +15.4 (95% CI 3.5–27.4; *p* = 0.011) for telephone coaching and +10.9 (−3.3 to 25.2; *p* = 0.130) for web coaching; no benefit was seen in the physical group.	Mechanism/contribution: Telecoaching mechanism: Monthly post-discharge coaching supports long-term HRQOL carry-over after MS MDR, especially when rehabilitation goals are neuropsychological and coaching is synchronous. Evidence stratum: Companion/secondary report.
**Study:** Trénel et al., 2024 [79] **Location/Country:** Denmark	**Aim:** Examine differential short- and long-term effects of inpatient MS rehabilitation by patient main focus area **Study design:** Pragmatic partial crossover randomized controlled trial analysis	**Disease group:** Multiple sclerosis. **Population:** N = 405 MS patients categorized by main focus area: Resilience *n* = 69, cognitive function *n* = 62, energy *n* = 135, physical function *n* = 142 and personal needs *n* = 12. Groups differed in age, MS type, EDSS, disease duration and baseline FAMS (all *p* ≤ 0.036).	**Rehabilitation/discharge anchor:** Four-week inpatient MDR with pre-admission patient-centred goal setting and MFA assignment.	**Post-discharge continuity component:** Post-discharge follow-up by MFA to examine differential persistence of MDR benefits.	**Comparator:** Wait-list/control sequence **Follow-up duration:** Discharge, 6-month and 12-month follow-up.	**Outcome measures:** FAMS total and subdimensions; MDR services received by MFA.	**Main findings:** All five MFA groups improved at discharge (FAMS change >10.4; *p* < 0.05). Controlled 6-month FAMS differences were resilience +9.9 (*p* = 0.001), cognitive function +5.6 (*p* = 0.196), energy +8.5 (*p* = 0.008), physical function −1.4 (*p* = 0.548) and personal needs +17.9 (*p* = 0.012), indicating variable persistence by goal domain.	Mechanism/contribution: Goal-setting mechanism shows that patient-centred rehabilitation goals and main focus areas shape both MDR content and the durability of post-discharge HRQOL gains. Evidence stratum: Companion/secondary report.
**Study:** Gunzler et al., 2025 [80] **Location/Country:** USA	**Aim:** Evaluate whether ≥3 daily mobilizations during hospitalization are associated with LOS and home-to-home discharge in PD **Study design:** Retrospective propensity score–matched study	**Disease group:** Parkinson’s disease. **Population:** From 25,555 admitted patients, 612 had PD. After matching, PD mobility group *n* = 153 and no-mobility group *n* = 153; matched controls without PD *n* = 6239 per group. PD patients were older and more often male than controls.	**Rehabilitation/discharge anchor:** Acute inpatient hospitalization; excluded obstetrics, rehabilitation admissions and ICU stay >24 h.	**Post-discharge continuity component:** Move to Heal inpatient mobility exposure: ≥3 mobilizations/day vs. <3 mobilizations/day; aligned with Parkinson’s Foundation Hospital Care Recommendations.	**Comparator:** Lower-mobility comparator **Follow-up duration:** Discharge outcomes and 30-day readmission.	**Outcome measures:** Length of stay, home-to-home discharge, 30-day readmission, insurance payout and complications.	**Main findings:** In PD, ≥3 daily mobilizations were associated with a 0.79-day shorter LOS (*p* = 0.019) and higher model-estimated probability of home-to-home discharge (65% vs. 50%; *p* = 0.004). In controls, LOS was 0.35 days shorter (*p* < 0.001). Mobility did not significantly affect 30-day readmission.	Mechanism/contribution: Mobility-readiness mechanism links inpatient mobilization frequency with shorter hospitalization and greater probability of home-to-home discharge in PD. Evidence stratum: Return-home/community re-entry evidence.
**Study:** Alnes et al., 2026 [81] **Location/Country:** Norway	**Aim:** Examine 6-month individualized mHealth self-management support after inpatient rehabilitation for physical capacity, PA, HRQOL and nutrition **Study design:** Single-blind two-arm randomized controlled trial	**Disease group:** Parkinson’s disease. **Population:** N = 100 post-rehabilitation participants randomized 1:1; 40% female; mean age 67.5 years; H&Y stage 1–3; recruited after 4–5-week inpatient interdisciplinary rehabilitation.	**Rehabilitation/discharge anchor:** Discharge from inpatient interdisciplinary Parkinson rehabilitation.	**Post-discharge continuity component:** Six-month individualized mHealth self-management support: Monthly video/phone consultations plus Garmin Vivosmart 4 activity tracker; PA, exercise and nutrition support.	**Comparator:** Usual care comparator **Follow-up duration:** 3- and 6-month follow-up.	**Outcome measures:** 6MWT primary; PG-SGA SF, PDQ-39, physical function, self-reported PA, daily steps and weekly intensity minutes.	**Main findings:** At 6 months, mHealth improved 6MWT by 33.1 m vs. control (95% CI 14.8–51.3; *p* < 0.001; ES = 0.75) and PDQ-39 SI by −6.1 (95% CI −9.5 to −2.8; *p* < 0.001; ES = 0.93). PA frequency favoured mHealth (*p* = 0.02; ES = 0.51); daily steps (*p* = 0.006) and weekly intensity minutes (*p* = 0.042) increased. No significant differences were found for nutritional status or physical function. In the mHealth group, 88% completed all six support sessions and 72% used the tracker for a median 23 weeks.	Mechanism/contribution: MHealth self-management mechanism extends inpatient PD rehabilitation into daily life through remote coaching, self-monitoring and nutrition/PA support, improving physical capacity, HRQOL and activity. Evidence stratum: Core transition evidence.
**Study:** Friedrich et al., 2026 [82] **Location/Country:** Germany	**Aim:** Characterize domain-specific effects, response predictors and 3-month persistence after inpatient PD-MCT **Study design:** Two-centre prospective open-label cohort trial	**Disease group:** Parkinson’s disease. **Population:** N = 109 screened; *n* = 67 baseline; *n* = 53 discharge complete-case; *n* = 44 at 3-month follow-up. Advanced idiopathic PD; mean age 70.7 ± 7.5 years; female 45.3%; median disease duration 11 years; median H&Y 2.5; MDS-UPDRS III 35.6 ± 13.1; PDQ-39 53.2 ± 18.9.	**Rehabilitation/discharge anchor:** Inpatient PD multimodal complex treatment for 14–21 days at two German centres; OPS-standardized multiprofessional care.	**Post-discharge continuity component:** Three-month structured telephone follow-up on symptom burden, physical activity and continuation of supportive therapies.	**Comparator:** No comparator **Follow-up duration:** Discharge and 3-month follow-up.	**Outcome measures:** MDS-UPDRS II/III/IV, PDQ-39, NMSQ, BDI, MoCA/PANDA, TUG, Tinetti, FRT, 6MWT and NHPT.	**Main findings:** PD-MCT improved motor symptom severity (MDS-UPDRS III −5.2 ± 10.6), overall motor function (MDS-UPDRS II + III + IV −10.6 ± 10.7) and QoL (PDQ-39 −9.3 ± 16.2). Symptom burden decreased from 6.1 ± 1.8 to 4.1 ± 2.0 (*p* < 0.001). Clinically meaningful MDS-UPDRS III response occurred in 32/53 (60.4%); 10/53 were stable and 11/53 worsened. Higher baseline motor burden and poorer QoL predicted larger short-term gains.	Mechanism/contribution: Multimodal complex-treatment carry-over mechanism maps short-term PD-MCT effects across motor, non-motor, cognitive and QoL domains and identifies high baseline burden as a response signal Evidence stratum: Return-home/community re-entry evidence.

Abbreviations: 2MWT = Two-Minute Walk Test; 6MWT = Six-Minute Walk Test; 10MT/10mWT = 10-Meter Walk Test; ADL = activities of daily living; ALDS = Amsterdam Linear Disability Scale; BBS = Berg Balance Scale; BDI = Beck Depression Inventory; EDSS = Expanded Disability Status Scale; FAMS = Functional Assessment of Multiple Sclerosis; FGS = fast gait speed; FIM = Functional Independence Measure; FRT = Functional Reach Test; FSMC = Fatigue Scale for Motor and Cognitive Functions; GHQ = General Health Questionnaire; HADS = Hospital Anxiety and Depression Scale; HIIT = high-intensity interval training; HRQOL = health-related quality of life; IEME = inpatient energy management education; LEDD = levodopa-equivalent daily dose; MDR = multidisciplinary rehabilitation; MFA = main focus area; MIRT = multidisciplinary intensive rehabilitation treatment; MoCA = Montreal Cognitive Assessment; MS = multiple sclerosis; MSIS-29 = Multiple Sclerosis Impact Scale-29; mHealth = mobile health; MDS-UPDRS = Movement Disorder Society-Unified Parkinson’s Disease Rating Scale; NHPT = Nine-Hole Peg Test; NMSQ = Non-Motor Symptom Questionnaire; NR = not reported in the available PDF/extraction; PA = physical activity; PD = Parkinson’s disease; PD-MCT = Parkinson’s disease multimodal complex treatment; PDQ-39 = Parkinson’s Disease Questionnaire-39; PG-SGA SF = Patient-Generated Subjective Global Assessment Short Form; PMR = progressive muscle relaxation; PP = primary progressive; RR = relapsing-remitting; SAMS = Stendal Adherence to Medication Score; SCOPA = Scales for Outcomes in Parkinson’s Disease; SEPECSA = Self-Efficacy for Performing Energy Conservation Strategies Assessment; SF-36 = 36-item Short Form Health Survey; SP = secondary progressive; SUS = System Usability Scale; TD = tremor dominant; TUG = Timed Up and Go. Evidence categories are standardized as core transition, return-home/community re-entry, adjacent continuity, or companion/secondary report.

**Table 5:** Actionable recommendations for neurological transition care by stakeholder.

Stakeholder	Minimum Actionable Components	Disease-Specific Examples	Implementation and Equity Safeguards	Supporting Included Evidence
**Clinicians**	Reconcile treatment; use teach-back; document risks and escalation thresholds; arrange early follow-up.	Dementia/ADRD: Medication and behavioural symptom plan. PD: Exact dopaminergic timing, adherence, mobility and falls. MS: Fatigue plan and home exercise. ALS: NIV, nutrition, equipment, symptom escalation and advance care planning.	Adapt for cognition, language, health literacy, communication disability and device access; offer written, telephone and in-person alternatives.	[24,25,45,48,58,74,77,80,81,83,95,99]
**Discharge planners**	Name the accountable patient/caregiver and contact; record origin, destination, treatment changes, follow-up, referrals, preparedness and required resources.	Dementia/ADRD: Caregiver plan and home–health linkage. PD: Medication timetable and mobility supports. MS: Portable rehabilitation goals. ALS: Respiratory, equipment, nutrition and palliative referrals.	Assess caregiver capacity, affordability, local service availability, transport, cultural and linguistic needs, and likely gaps before discharge.	[40,42,44,51,55,60,63,74]
**Health systems**	Assign a coordinator/champion; enable interoperable records and cross-setting referral; monitor reach, outcomes, burden and sustainability; maintain non-digital routes.	Integrate dementia navigation/home health, PD medication safety, MS rehabilitation aftercare, and ALS respiratory/telehealth/palliative escalation into accountable pathways.	Audit uptake and outcomes by socioeconomic, racial/ethnic, linguistic, geographic, disability and caregiver-capacity strata; fund access supports.	[43,56,57,65,66,89,93,96,99,102]

## Data Availability

The original contributions presented in this study are included in the article and Supplementary Materials. Further inquiries can be directed to the corresponding author. The study protocol is publicly available on the Open Science Framework (OSF) at https://doi.org/10.17605/OSF.IO/US27J.

## Supplementary Materials

The following supporting information can be downloaded at https://www.mdpi.com/article/10.3390/medsci14040445/s1. The combined supplementary file contains the PCC-based methodological framework and operational subtables in Supplementary Table S1 (S1A–S1H). Supplementary Table S2 reports the search yield audit; Supplementary Table S3 includes the disease-by-category distribution and study-level evidence classification; Supplementary Tables S4–S8 provide the care model taxonomy, outcome-domain map, technology-enabled continuity models, caregiver role framework, and methodological gap map; Supplementary Table S9 provides component-to-study traceability for the proposed frameworks; and Supplementary Figures S1 and S2 present the proposed conceptual frameworks.

## Abbreviations

ACP, advance care planning; ADRD, Alzheimer’s disease and related dementias; ALS, amyotrophic lateral sclerosis; ED, emergency department; HHA, home health agency; JBI, Joanna Briggs Institute; MS, multiple sclerosis; NIV, non-invasive ventilation; OSF, Open Science Framework; PCC, population–concept–context; PD, Parkinson’s disease; PICO, population–intervention–comparator–outcomes; PRISMA-ScR, Preferred Reporting Items for Systematic Reviews and Meta-Analyses Extension for Scoping Reviews; SNF, skilled nursing facility.
