# Supplementary material for: Hospital-to-Home Neurological Transition Care: A Scoping Review Across Selected Chronic Neurological Disorders
Source: Med Sci (Basel). 2026 Jul 28;14(4):445. doi: 10.3390/medsci14040445 (PMC13515623; doi:10.3390/medsci14040445)
Supplement: Supplementary file 1 [file medsci-14-00445-s001.zip › medsci-4417792-supplementary.pdf]

medical sciences

MDPI

Supplementary Material: Hospital-to-Home Neurological Transition Care: A Scoping Review Across Selected Chronic Neurological Disorders

Rocco Salvatore Calabrò, Andrea Calderone, Daniele Ravi, Carmelo Galipò, Maria Felicita Crupi and Angelo Quartarone

PCC-based methodological framework and evidence-mapping tables

This combined supplementary material supports the scoping review of post-discharge follow-up, transitional care, return-home trajectories, rehabilitation continuity, and community re-entry in the selected chronic neurological populations of Parkinson's disease, dementia/ADRD, multiple sclerosis, and ALS. Supplementary Table S1 uses PCC as the primary eligibility and synthesis framework; PICO-derived comparator and outcome fields are retained only as auxiliary search and charting variables. Supplementary Tables S2–S9 provide evidence-mapping, audit, and framework-traceability tables, while Supplementary Figures S1 and S2 present the proposed conceptual frameworks.

Supplementary Table S1. PCC-based methodological framework and operational subtables

Subtable S1A. PCC framework with auxiliary PICO-derived charting fields

Framework element	Operational definition	Eligible examples	Boundary rules
Population	Adults or adult-dominant populations with Parkinson's disease, dementia/Alzheimer's disease and related dementias, multiple sclerosis, or amyotrophic lateral sclerosis.	Adults leaving acute hospital, inpatient rehabilitation, skilled nursing or subacute care, specialist or multidisciplinary care, or organized neurological services; mixed samples when target-condition data are separable or clearly predominant.	Exclude pediatric-only studies unless adult data are separable. Mixed samples are eligible only when target-condition data can be extracted or the eligible population clearly predominates.
Concept	Post-discharge follow-up, transitional care, discharge planning, return-home support, rehabilitation continuity, home or community re-entry, case management, telehealth or telerehabilitation, caregiver support, medication or treatment continuity, integrated care, respiratory or nutritional continuity, palliative-care continuity, and advance care planning.	Follow-up calls, home visits, medication reconciliation, caregiver education, post-rehabilitation coaching, home-health linkage, home NIV monitoring, multidisciplinary telehealth, palliative follow-up, and advance-care-planning support.	The concept must concern continuity across a transition or the preservation of care after institutional or organized specialist care. Generic outpatient monitoring without transition relevance is insufficient.
Context	Transition from acute hospital, inpatient rehabilitation, skilled nursing or subacute care, specialist or multidisciplinary care, or another organized neurological service to home, community, outpatient, primary-care, home-health, home-palliative, or everyday-life settings.	Hospital-to-home, SNF-to-home, rehabilitation-to-community, clinic-to-home telehealth, home-health episodes, community rehabilitation, and specialist home palliative or respiratory care.	Exclude inpatient-only episodes, institution-only trajectories without home or community continuity, and routine clinic follow-up without an explicit or inferable transition context.

Framework element	Operational definition	Eligible examples	Boundary rules
Auxiliary PICO-derived charting fields	Comparator and outcome fields were retained to support searching, charting, and interpretation, but they did not replace PCC as the eligibility framework. Comparator presence was not required.	Usual care, conventional discharge, baseline, alternative model, or no comparator; patient, caregiver, safety, rehabilitation, service-use, feasibility, equity, respiratory, nutritional, palliative, and implementation outcomes.	Do not exclude non-comparative, qualitative, implementation, or service-evaluation evidence solely because a comparator is absent. Outcomes must be measured or described after transition or in relation to home or community continuity.

Abbreviations: ACP = advance care planning; ADRD = Alzheimer's disease and related dementias; ALS = amyotrophic lateral sclerosis; NIV = non-invasive ventilation; PCC = Population–Concept–Context; PICO = Population–Intervention–Comparator–Outcomes.

Subtable S1B. PCC-based eligibility criteria

Domain	Inclusion criteria	Exclusion criteria	Operational notes
Study type	Peer-reviewed primary empirical quantitative, qualitative, or mixed-methods studies, including trials, cohort studies, before-after studies, implementation studies, service evaluations with empirical data, qualitative studies, and mixed-methods studies.	Reviews, systematic reviews, scoping reviews, meta-analyses, editorials, commentaries, protocols without results, letters, opinion pieces, conference abstracts without sufficient data, theses, book chapters, and non-human studies.	Relevant reviews may be hand-searched for primary studies but should not be included in the evidence map.
Disease population	Parkinson's disease, dementia including Alzheimer's disease and related neurodegenerative dementias, multiple sclerosis, or amyotrophic lateral sclerosis.	Stroke, traumatic brain injury, spinal cord injury, Huntington disease, epilepsy, generic frailty samples, vascular dementia, post-stroke dementia, vascular cognitive impairment, or mixed neurological samples without separable or predominant eligible data.	If a mixed sample includes eligible diseases, include only if target-disease data are separable or the eligible group is clearly predominant. For dementia, exclude purely vascular or non-neurodegenerative etiologies unless a neurodegenerative subgroup is separable.
Age group	Adults or adult-dominant mixed-age samples.	Pediatric-only studies without adult data.	Adult-dominant mixed-age samples may be included when the clinical context is relevant to adult discharge or rehabilitation pathways.
Context: transition origin	Explicit or inferable discharge from acute hospital, inpatient rehabilitation, subacute care, specialist care, or comparable organized neurological care. Explicit anchors included stated discharge or immediate post-discharge timing; inferable anchors were based on pathway timing, intervention start, home/community destination, or follow-up after organized care.	Routine outpatient follow-up unrelated to discharge; disease monitoring without care transition; inpatient-only episodes.	Err on inclusion during title/abstract screening when discharge or organized-care continuity is plausible; require explicit or inferable transition at full-text stage. When no discrete discharge point is identifiable but home/community continuity is relevant, classify as adjacent continuity evidence rather than core transition evidence.
Context: destination	Home, community, outpatient follow-up, primary care, home care, home-based rehabilitation, community rehabilitation, telehealth, telerehabilitation, or everyday-life re-entry.	Long-term institutional care only; nursing-home-only trajectories without discharge-to-home pathway; inpatient-only rehabilitation.	Return home may be literal discharge destination or functional/community re-entry after discharge.
Intervention or exposure	Transitional care, follow-up, rehabilitation continuity, case management, caregiver support, telehealth, medication-management support, integrated care, self-management support, readmission-prevention pathway, home	Drug efficacy, device efficacy, disease-modifying treatment, or biological treatment studies without a discharge, continuity, home/community transition, respiratory-continuity, palliative-continuity, or service-delivery component.	Observational follow-up studies are eligible when they describe the post-discharge trajectory or outcomes.

https://doi.org/10.3390/medsci14040445

Domain	Inclusion criteria	Exclusion criteria	Operational notes
	NIV/ventilation support, respiratory or nutritional continuity, palliative-care continuity, advance care planning, or usual-care post-discharge follow-up.		
Outcomes	At least one patient, caregiver, service-use, safety, rehabilitation, participation, quality-of-life, feasibility, acceptability, respiratory, nutritional, palliative, end-of-life, or implementation outcome after discharge or transition.	No post-discharge outcomes; outcomes measured only before discharge; purely administrative coding without transition or care-model relevance.	Accept quantitative outcomes and qualitative themes concerning experience, barriers, facilitators, or implementation.
Language and date	English-language full-text articles published from database inception through 11 May 2026.	Non-English full texts; reports published after the final search cut-off; full texts unavailable after reasonable retrieval attempts.	No lower date limit was applied. Language and date restrictions were applied consistently at full-text eligibility.

Abbreviations: ACP = advance care planning; ADRD = Alzheimer's disease and related dementias; ALS = amyotrophic lateral sclerosis; NIV = non-invasive ventilation.

Subtable S1C. Final database-specific Boolean search strategies and syntax notes

All electronic searches were run from database inception to 11 May 2026. No date limit was applied at the search stage. English-language full-text eligibility was applied at full-text screening. Field tags, controlled vocabulary, truncation, and proximity operators were adapted to each database interface; comparator terms were not mandatory search blocks.

PubMed/MEDLINE syntax: disease terms were searched in Title/Abstract and, where available, mapped to MeSH headings (for example Dementia, Alzheimer Disease, Parkinson Disease, Multiple Sclerosis, Amyotrophic Lateral Sclerosis, Patient Discharge, Continuity of Patient Care, Telemedicine, Home Care Services, Rehabilitation, Palliative Care). Core free-text blocks used [tiab] field tags, quoted phrases, and truncation where supported.

Embase syntax: controlled vocabulary was searched with Emtree terms and explosion where appropriate, combined with title/abstract/keyword terms using :ti,ab,kw. Proximity operators were adapted to the Embase interface when phrase searching alone was insufficient for terms such as post-discharge, hospital-to-home, home care, telemonitoring, non-invasive ventilation, and palliative care.

Web of Science Core Collection syntax: topic searches used TS= blocks with quoted phrases, Boolean operators, truncation, and NEAR/x proximity operators where applicable. Scopus syntax used TITLE-ABS-KEY blocks with W/n proximity operators when appropriate. Cochrane Library syntax used title/abstract/keyword searches and MeSH descriptors where available.

Database-level retrieved records were PubMed/MEDLINE n=1,023, Web of Science n=862, Cochrane Library/Reviews n=252, Embase n=1,822, and Scopus n=20,458, for 24,417 records before deduplication. Cochrane Library/Cochrane Reviews records were exported and reported as one Cochrane source within the five-database PRISMA structure.

Disease-domain strategy	Final Boolean search string	Target evidence domain	Database use and notes
Dementia and ADRD transition evidence	(dementia OR Alzheimer OR ADRD) AND ("hospital-to-home" OR "transitional care" OR "post-discharge" OR "discharge planning" OR "home	Table 1: hospital-to-home transition, discharge planning, caregiver-mediated continuity, medication management, home health, readmission and return-home evidence.	Used across PubMed/MEDLINE, Web of Science Core Collection, Embase, Scopus and Cochrane Library with database-specific field tags, controlled vocabulary where applica-

Disease-domain strategy	Final Boolean search string	Target evidence domain	Database use and notes
	health") AND (caregiver OR "medication management" OR readmission OR "return home")		ble, truncation, and proximity syntax adapted to the interface.
Parkinson disease and multiple sclerosis rehabilitation-continuity evidence	("Parkinson disease" OR "Parkinson's disease" OR "multiple sclerosis") AND ("inpatient rehabilitation" OR "multidisciplinary rehabilitation" OR telerehabilitation OR mHealth) AND ("post-discharge" OR follow-up OR "carry-over" OR "return home")	Table 2: inpatient rehabilitation, multidisciplinary rehabilitation, telerehabilitation, mHealth, post-discharge follow-up, carry-over and return-home evidence.	Used across PubMed/MEDLINE, Web of Science Core Collection, Embase, Scopus and Cochrane Library with database-specific field tags and proximity syntax. The string was intentionally focused to avoid excessive retrieval from generic rehabilitation literature.
Amyotrophic lateral sclerosis home-based, telehealth, respiratory and palliative-continuity evidence	("amyotrophic lateral sclerosis" OR "motor neuron disease") AND (telemedicine OR telehealth OR telemonitoring OR "home care" OR "palliative care" OR "non-invasive ventilation") AND (follow-up OR continuity OR caregiver OR "advance care planning")	Table 3: ALS telemedicine, telehealth, telemonitoring, home care, palliative care, non-invasive ventilation, follow-up, continuity, caregiver and advance-care-planning evidence.	Used across PubMed/MEDLINE, Web of Science Core Collection, Embase, Scopus and Cochrane Library with database-specific field tags and controlled vocabulary where applicable. The ALS abbreviation was not used as a standalone term to reduce irrelevant retrieval.

Abbreviations: ADRD = Alzheimer’s disease and related dementias; ALS = amyotrophic lateral sclerosis; MS = multiple sclerosis; MND = motor neuron disease; NIV = non-invasive ventilation.

Subtable S1D. Screening decision rules

Screening issue	Include	Exclude	Resolution rule
Unclear discharge context	Include at title/abstract stage if follow-up after hospital, rehabilitation, specialist, or subacute discharge is plausible.	Exclude at full text if no discharge, transition point, or return-home/community continuity component can be identified.	Use conservative title and abstract screening; apply the PCC transition context explicitly at full text.
Mixed neurological samples	Include if Parkinson's disease, dementia/ADRD, multiple sclerosis, or amyotrophic lateral sclerosis data are separable or clearly predominant.	Exclude if eligible diseases are a minor, non-separable subgroup.	Record reviewer judgement and whether disease-specific data were extractable.
Home versus institution	Include discharge home, home care, community care, outpatient follow-up, community rehabilitation, or everyday-life re-entry.	Exclude long-term institutional care without a discharge-to-home or community re-entry pathway.	If discharge destinations include both home and institution, chart subgroup information when available.
Telehealth or telerehabilitation	Include when remote care is linked to post-discharge follow-up, rehabilitation continuity, return-home support, caregiver support, or self-management after institutional/specialist care.	Exclude remote monitoring or telehealth unrelated to discharge, transition, rehabilitation carry-over, or community re-entry.	Code mode of delivery separately from intervention function.
Medication management	Include caregiver or professional support for medication continuity after discharge.	Exclude drug efficacy, dosing, or disease-modifying treatment studies without transition-of-care relevance.	Chart medication support as a component of transitional care or caregiver-mediated continuity.
Readmission studies	Include if the disease population and post-discharge pathway, follow-up model, or care process are specified.	Exclude administrative readmission-only studies without transition, follow-up, care-model, or patient/caregiver process information unless clearly relevant.	Use caution when the study provides no patient-centred, caregiver-centred, or process data beyond readmission.
Caregiver-focused studies	Include when caregiver outcomes or caregiver preparation relate to discharge, return home, medication management, safety, or community navigation for an eligible disease.	Exclude caregiver studies unrelated to discharge or post-discharge continuity.	Chart whether caregivers are intervention recipients, informants, proxies, or outcome participants.
Multiple sclerosis rehabilitation	Include inpatient rehabilitation carry-over, home-based management, telecoaching, telerehabilitation, self-management or functional follow-up after inpatient/specialist care.	Exclude routine disease-modifying therapy monitoring without discharge or continuity-of-care component.	Document relapse/progression context, disability level, fatigue, and participation outcomes where reported.

https://doi.org/10.3390/medsci14040445

Screening issue	Include	Exclude	Resolution rule
Amyotrophic lateral sclerosis continuity	Include home-based care, telehealth, telemedicine, telemonitoring, NIV/home ventilation, multidisciplinary follow-up, caregiver support, palliative-care continuity, advance care planning, or end-of-life pathway studies when they report empirical outcomes. When the discharge point is not discrete or timed, classify as adjacent continuity evidence.	Exclude ALS studies focused only on drug, biological, respiratory-device efficacy, or clinic-only monitoring without a home/community, follow-up, palliative, respiratory-continuity, or service-delivery component.	Chart respiratory, nutritional, palliative, caregiver and service-use components separately because these are central to ALS community trajectories.

Abbreviations: ACP = advance care planning; ADRD = Alzheimer's disease and related dementias; ALS = amyotrophic lateral sclerosis; MS = multiple sclerosis; NIV = non-invasive ventilation.

Subtable S1E. Evidence-classification and overlap-control framework

Evidence category	Operational definition	Eligible examples	Interpretive use
Core transition evidence	Reports explicitly beginning at or immediately after discharge and evaluating structured follow-up, transitional care, return-home support, rehabilitation continuity, case management, medication support, integrated care, or readmission-prevention pathways.	Post-discharge case management; caregiver-supported dementia discharge; medication follow-up after Parkinson's hospitalization; telecoaching after inpatient MS rehabilitation.	Most direct evidence for discharge-anchored transition models, while remaining heterogeneous in design, comparator, intensity, and follow-up.
Return-home/community re-entry evidence	Reports examining functional, participation, caregiver, safety, equity, or service-use trajectories after discharge when the care model is less structured or not labeled transitional care.	Functional carry-over after inpatient rehabilitation; home-to-home discharge; community stability; caregiver burden or home-health quality after discharge.	Defines how return home and community re-entry are operationalized but may not test a discrete transition intervention.
Adjacent continuity evidence	Reports on home-based, remote, rehabilitation, respiratory, nutritional, caregiver, outpatient, or palliative continuity with a less explicit discharge anchor, or in which continuity represents ongoing specialist/community management rather than a discrete discharge event. For ALS, repeated respiratory, nutritional, technological, and palliative adjustments may additionally be described as a continuous, progression- or needs-triggered continuity pathway. This is a descriptive sublabel within adjacent continuity, not a fifth category, and does not alter allocation or counts.	ALS telemonitoring, home ventilation, multidisciplinary telehealth, home palliative care, or remote rehabilitation not clearly initiated by a specific discharge.	Informs components, feasibility, delivery mechanisms, escalation functions, and implementation boundaries; it is not direct evidence of discharge-model effectiveness and may underrepresent conditions with continuous rather than discrete transitions.
Companion/secondary report	A report arising from an overlapping program or cohort that contributes distinct mechanistic, outcome, implementation, or subgroup information.	Fam-FFC, Connect-Home ADRD, Danish MS Hospitals, ALS Home Monitoring and Coaching, or home palliative-care companion analyses.	Retained to enrich interpretation but not treated as independent replication of the parent program or cohort.

Abbreviations: ADRD = Alzheimer's disease and related dementias; ALS = amyotrophic lateral sclerosis; MS = multiple sclerosis; NIV = non-invasive ventilation. Evidence categories describe transition proximity or reporting overlap, not methodological quality, sample size adequacy, comparator strength, or certainty of evidence.

Subtable S1F. Data-charting form and verification procedure

https://doi.org/10.3390/medsci14040445

Domain	Variables to chart	Purpose in synthesis
Charting procedure	One reviewer (A.C.) charted each included report using the standardized form. R.S.C. and A.Q. verified transition anchors, dominant mechanisms, evidence categories, and overlap status; disagreements or uncertainties were resolved by discussion among R.S.C., A.C. and A.Q., with adjudication by A.Q. when required.	Ensure transparent verification, consistent application of operational definitions, and alignment between charted data, evidence categories, and synthesis statements.
Bibliographic data	Author, year, journal, country, funding, conflicts of interest if reported.	Describe publication distribution and geographical context.
Study design	Design, aims, recruitment source, comparator, randomization if applicable, sample size, follow-up schedule.	Map methodological diversity and strength of available evidence.
Population	Disease group, diagnostic criteria if reported, age, sex/gender, disease stage or severity, cognitive status, disability level, respiratory/nutritional support, palliative-care status, comorbidity, living situation.	Compare target populations and identify under-described subgroups.
Discharge context	Acute hospital, inpatient rehabilitation, subacute care, specialist service, discharge destination, timing of enrolment relative to discharge.	Define the starting point of the transition.
Post-discharge destination	Home, community care, outpatient follow-up, primary care, home care, community rehabilitation, telehealth, telerehabilitation, institutional destination if mixed.	Clarify whether return home is literal, functional, service-based, or participation-based.
Care model/intervention	Discharge planning, case management, home-based follow-up, home rehabilitation, multidisciplinary follow-up, telehealth, caregiver training, medication support, readmission-prevention pathway, integrated care, usual care.	Classify post-discharge transition models and active components.
Personnel and delivery	Professionals involved, caregiver role, mode of delivery, frequency, dose, duration, technology platform, coordination mechanism, personalization.	Identify who delivers continuity and how it is operationalized.
Comparator	Usual care, conventional discharge, baseline, alternative model, no comparator, not applicable.	Support interpretation without excluding non-comparative evidence.
Outcomes and instruments	Readmission, emergency visits, adverse events, falls, medication errors, function, mobility, activities of daily living, cognition, fatigue, participation, quality of life, caregiver burden, preparedness, adherence, satisfaction, service use, implementation outcomes, measurement tools.	Map outcome domains and measurement heterogeneity.
Implementation and equity	Feasibility, acceptability, adherence, fidelity, workforce needs, digital access, accessibility adaptations, barriers, facilitators, equity issues.	Assess translation into real post-discharge pathways.
Key findings and limitations	Main results, qualitative themes, author-reported limitations, follow-up completeness, missing data, safety reporting.	Support narrative synthesis and gap analysis.
Evidence category	Core transition evidence, return-home/community re-entry evidence, adjacent continuity evidence, or companion/secondary report.	Maintain inferential discipline and prevent overlapping reports from being interpreted as independent replication.

Abbreviations: A.C. = Andrea Calderone; A.Q. = Angelo Quartarone; ADL = activities of daily living; ADRD = Alzheimer's disease and related dementias; ALS = amyotrophic lateral sclerosis; QoL = quality of life; R.S.C. = Rocco Salvatore Calabrò.

Subtable S1G. Outcome and synthesis taxonomy

Outcome domain	Examples	Disease-specific considerations	Synthesis output
Safety and service use	Readmission, emergency department use, adverse events, falls, complications, medication errors, unplanned contacts, hospitalization duration, service utilization.	Parkinson's disease: dopaminergic timing, falls, delirium, aspiration risk. Dementia: medication safety, wandering, caregiver-mediated monitoring. Multiple sclerosis: relapse/disability context and fatigue-related safety. Amyotrophic lateral sclerosis: respiratory deterioration, NIV/ventilation support, nutritional support, emergency use and palliative transitions.	Frequency and type of safety and service-use outcomes by disease and care model.

Outcome domain	Examples	Disease-specific considerations	Synthesis output
Functional recovery	Activities of daily living, mobility, balance, transfers, gait, upper-limb function, fatigue, rehabilitation goal attainment.	Avoid inappropriate pooling across disease-specific instruments. Multiple sclerosis fatigue and disability should be interpreted with disease trajectory. Parkinson's disease outcomes should consider fluctuations.	Narrative comparison of functional domains, instruments and follow-up timing.
Cognitive and neuropsychological outcomes	Cognition, executive function, memory, attention, delirium, behavioural symptoms, neuropsychiatric symptoms.	Dementia may require proxy reporting and caregiver interpretation. Parkinson's disease may include cognitive fluctuations or delirium risk. Multiple sclerosis may include cognitive fatigue and processing speed.	Map cognitive outcomes and identify whether they are primary targets, safety markers, or contextual variables.
Participation and community re-entry	Return to everyday activities, social participation, community reintegration, self-management, return to usual roles, environmental adaptation.	Often under-measured. Qualitative evidence may be important across all three diseases. Multiple sclerosis studies may emphasize participation and self-management; dementia studies may rely on caregiver reports.	Concept map of how return home and community re-entry are operationalized.
Quality of life and patient experience	Health-related quality of life, satisfaction, perceived continuity, confidence, experience of discharge, perceived preparedness, emotional well-being.	Dementia studies may use proxy-reported quality of life. Parkinson's disease and multiple sclerosis may require disease-specific quality-of-life tools.	Summary of patient-centred outcome use and reporting gaps.
Caregiver outcomes	Caregiver burden, preparedness, confidence, medication-management competence, strain, satisfaction, perceived support, navigation workload.	Central in dementia and relevant in advanced Parkinson's disease, multiple sclerosis and amyotrophic lateral sclerosis. Distinguish caregiver as participant, proxy, intervention recipient, or informal service coordinator.	Description of caregiver involvement, measured outcomes and underreported caregiver domains.
Medication and treatment continuity	Medication reconciliation, adherence, timing accuracy, caregiver medication guidance, treatment-plan communication, adverse drug events.	Parkinson's disease: dopaminergic timing and medication interruptions. Dementia: caregiver-mediated medication safety. Multiple sclerosis: distinguish symptomatic treatment, disease-modifying therapy, and rehabilitation continuity.	Map medication continuity components and whether outcomes are measured or only described.
Rehabilitation continuity	Home exercises, outpatient rehabilitation linkage, home-based rehabilitation, telerehabilitation, telecoaching, equipment use, goal carry-over, home monitoring, respiratory/nutritional continuity, and palliative-care continuity.	Parkinson's disease: mobility, falls and non-motor symptoms. Dementia: caregiver-supported function and safety. Multiple sclerosis: fatigue, disability, participation and rehabilitation carry-over. Amyotrophic lateral sclerosis: home ventilation, telemonitoring, multidisciplinary home care and palliative-care continuity.	Classification of rehabilitation-continuity models and follow-up intensity.
Implementation and equity	Feasibility, acceptability, adherence, fidelity, workforce requirements, digital access, technology barriers, service fragmentation, rural access, socioeconomic barriers.	Remote models may exclude patients with cognitive impairment, severe disability, sensory impairment, limited caregiver support, or limited digital access.	Narrative synthesis of barriers, facilitators, accessibility requirements and implementation readiness.

Abbreviations: ACP = advance care planning; ADL = activities of daily living; ADRD = Alzheimer's disease and related dementias; ALS = amyotrophic lateral sclerosis; ED = emergency department; ER = emergency room; HRQOL = health-related quality of life; NIV = non-invasive ventilation.

Subtable S1H. Planned reporting structure

Manuscript component	Content to report	Format
Study selection	PRISMA flow, records identified, duplicates removed, records screened, full texts assessed, included studies, and main full-text exclusion reasons.	Flow diagram plus concise narrative with placeholders replaced after final screening.
Evidence landscape	Distribution of studies by disease, country, design, setting, discharge context, post-discharge destination, follow-up duration, care model and outcome domain.	Narrative text plus summary tables or figures.

https://doi.org/10.3390/medsci14040445

Manuscript component	Content to report	Format
Disease-specific synthesis	Separate synthesis for Parkinson's disease, dementia/ADRD, multiple sclerosis, and amyotrophic lateral sclerosis, emphasizing transition needs, care models, caregiver role and outcome domains.	Main-text subsections with disease-domain evidence tables in supplementary materials or main manuscript tables.
Cross-cutting care models	Discharge planning, case management, home-based follow-up, rehabilitation continuity, telehealth/telerehabilitation, caregiver-supported discharge, medication-management support, integrated care, and readmission-prevention pathways.	Conceptual map and narrative synthesis.
Outcome map	Safety/service use, functional recovery, cognition, participation/community re-entry, quality of life, patient experience, caregiver outcomes, medication continuity, rehabilitation continuity, implementation and equity.	Outcome taxonomy table plus disease-specific narrative.
Evidence-stratification summary	Counts and proportions for core transition, return-home/community re-entry, adjacent continuity, and companion/secondary reports, overall and by disease group.	Disease-by-category matrix plus study-level classification and an interpretive statement distinguishing transition proximity from methodological quality.
Gap analysis	Underrepresented diseases, discharge settings, caregiver outcomes, long-term follow-up, participation measures, implementation reporting, equity and digital-access issues.	Reviewer-proof discussion anchored to mapped evidence.
Methodological quality and interpretation	No risk-of-bias exclusion for scoping purposes; chart design, comparator, follow-up completeness, outcome measurement and reporting clarity descriptively.	Methods statement and optional supplementary methodological-feature table.

Abbreviations: ADRD = Alzheimer’s disease and related dementias; ALS = amyotrophic lateral sclerosis; PRISMA = Preferred Reporting Items for Systematic Reviews and Meta-Analyses.

Supplementary Table S2. PRISMA search yield and database-level contribution

Search string	Database	Raw records	Contribution (%)	Notes
String 1	PubMed	365	1.49	
String 1	Web of Science	334	1.37	
String 1	Cochrane Library/Reviews	69	0.28	Cochrane Library and Cochrane Reviews records were exported and reported together as one Cochrane source within the five-database PRISMA structure.
String 1	Embase	600	2.46	
String 1	Scopus	10,503	43.02	
String 2	PubMed	196	0.80	
String 2	Web of Science	127	0.52	
String 2	Cochrane Library/Reviews	104	0.43	Cochrane Reviews were merged under Cochrane Library for the 5-database PRISMA structure.
String 2	Embase	264	1.08	
String 2	Scopus	5,274	21.60	
String 3	PubMed	462	1.89	
String 3	Web of Science	401	1.64	
String 3	Cochrane Library/Reviews	79	0.32	Cochrane Reviews were merged under Cochrane Library for the 5-database PRISMA structure.

https://doi.org/10.3390/medsci14040445

Search string	Database	Raw records	Contribution (%)	Notes
String 3	Embase	958	3.92	
String 3	Scopus	4,681	19.17	
Overall total	5 databases	24,417	100.00	Records identified before deduplication.

Abbreviations: PRISMA = Preferred Reporting Items for Systematic Reviews and Meta-Analyses.

Supplementary Table S3. Evidence classification of included reports

Panel A. Distribution by disease group and evidence category

Disease group	Core transition evidence	Return-home/community re-entry evidence	Adjacent continuity evidence	Companion/secondary reports	Total
Dementia/ADRD	14	3	0	11	28
Parkinson's disease	3	7	0	0	10
Multiple sclerosis	1	4	0	4	9
ALS	0	0	16	6	22
Overall, n (%)	18 (26.1%)	14 (20.3%)	16 (23.2%)	21 (30.4%)	69 (100%)

Percentages in the overall row use all 69 included reports as the denominator. Companion/secondary reports were retained for distinct information from overlapping programs or cohorts and were not interpreted as independent replication.

Panel B. Study-level classification

Study/reference	Disease population	Evidence category	Rationale for classification	Main transition mechanism
[40] Cummings et al., 1999	Dementia/ADRD	Core transition evidence	Discharge-plan adequacy and 1-month readmission after dementia hospitalization	Discharge-plan adequacy, family readiness and caregiver support

Study/reference	Disease population	Evidence category	Rationale for classification	Main transition mechanism
[41] Villars et al., 2013	Dementia/ADRD	Core transition evidence	Structured follow-up after Special Alzheimer Acute Care Unit discharge	Scheduled caregiver/resource-person calls
[42] Kable et al., 2015	Dementia/ADRD	Core transition evidence	Professional perspectives on discharge and transitional-care failures	Documentation, service access and medication-order failure mapping
[43] Boltz et al., 2015	Dementia/ADRD	Companion/secondary report	Early Fam-FFC feasibility/preliminary efficacy report	Family-centered function-focused recovery support
[44] Kable et al., 2019	Dementia/ADRD	Core transition evidence	Audit of dementia discharge documentation	Risk, medication, referral and caregiver information quality
[45] Bruce et al., 2020	Dementia/ADRD	Core transition evidence	Medication-management support after discharge	Caregiver-mediated medication continuity
[46] Knox et al., 2020	Dementia/ADRD	Companion/secondary report	Medicare/OASIS home-health readmission analysis	Dementia severity as readmission risk marker
[47] Knox et al., 2020b	Dementia/ADRD	Companion/secondary report	Home-health readmission predictors in ADRD	Mobility, self-care, cognition and caregiver-support risk
[48] Sawan et al., 2021	Dementia/ADRD	Core transition evidence	Caregiver experiences of medication guidance at discharge	Medication information, engagement and supply continuity
[49] Knox et al., 2022	Dementia/ADRD	Companion/secondary report	Home-health therapy completion analysis	Therapy continuity and caregiver/medication support
[50] Knox et al., 2022b	Dementia/ADRD	Companion/secondary report	Home-health utilization and discharge-to-community analysis	Episode duration, therapy utilization and community stability
[51] Toles et al., 2022b	Dementia/ADRD	Core transition evidence	Needs assessment for SNF-to-home dementia transitions	Readiness, symptom management and community linkage
[52] Toles et al., 2022	Dementia/ADRD	Companion/secondary report	Connect-Home ADRD feasibility cohort	Dyad-centered SNF transitional care
[53] Kovaleva et al., 2023	Dementia/ADRD	Core transition evidence	Post-hospital caregiver tele-education design study	Tele-education and caregiver psychoeducation
[54] Du Preez et al., 2023	Dementia/ADRD	Core transition evidence	Carer needs after ED/hospital discharge in advanced dementia	Palliative needs assessment and future planning
[55] Saragosa et al., 2023	Dementia/ADRD	Core transition evidence	Grounded theory of hospital-to-home transition	Caregiver observation, navigation and connecting dots
[56] Temkin-Greener et al., 2023	Dementia/ADRD	Return-home/community re-entry evidence	PAC pathway and outcomes analysis	SNF, home-health and home-without-services pathways
[57] Boltz et al., 2023	Dementia/ADRD	Companion/secondary report	Definitive Fam-FFC cluster RCT	Family-centered function-focused care and postacute calls
[58] Berish et al., 2024	Dementia/ADRD	Companion/secondary report	Secondary analysis of BPSD and adverse outcomes	Behavioral risk markers for ER use and hospitalization
[59] Drazich et al., 2025	Dementia/ADRD	Return-home/community re-entry evidence	Physical resilience and post-discharge adverse events	Resilience as risk profile
[60] Horstman et al., 2024	Dementia/ADRD	Core transition evidence	Caregiver needs in VA care transitions	Caregiver partnership, dementia information and navigation
[61] Knox et al., 2024	Dementia/ADRD	Companion/secondary report	Home-health discharge-to-community analysis	Dementia severity, medication and caregiver support
[62] Paudel et al., 2024	Dementia/ADRD	Companion/secondary report	Care-partner preparedness secondary analysis	Burden, strain and depression as preparedness determinants
[63] Toles et al., 2024	Dementia/ADRD	Core transition evidence	Transitional palliative care in ADRD-PC	Palliative-care plan plus post-discharge calls
[64] Toles et al., 2024b	Dementia/ADRD	Companion/secondary report	Health trajectories after SNF discharge	Post-SNF trajectory and recurrent acute event mapping
[65] Yang et al., 2024	Dementia/ADRD	Return-home/community re-entry evidence	Home-health quality disparities after ADRD hospitalization	Equity/access to HHA quality
[66] Greyson et al., 2025	Dementia/ADRD	Core transition evidence	Latino caregiver hospital-to-home qualitative study	Cultural tailoring and task ambiguity
[67] Idso et al., 2025	Dementia/ADRD	Core transition evidence	Nurse-led caregiver discharge-call QI project	4Ms caregiver call and tailored resources
[21] Freeman et al., 1999	Multiple sclerosis	Return-home/community re-entry evidence	Inpatient rehabilitation carry-over after discharge	Community carry-over of disability, handicap and HRQOL gains
[22] Pozzilli et al., 2002	Multiple sclerosis	Return-home/community re-entry evidence	Home-based management versus hospital care	Interdisciplinary home care and cost/quality-of-life effects
[24] Flachenecker et al., 2020	Multiple sclerosis	Core transition evidence	Internet-based aftercare after inpatient rehabilitation	Digital exercise/PA aftercare for fatigue and HRQOL carry-over

https://doi.org/10.3390/medsci14040445

Study/reference	Disease population	Evidence category	Rationale for classification	Main transition mechanism
[25] Nørgaard et al., 2024	Multiple sclerosis	Companion/secondary report	Telecoaching after inpatient MDR	Goal-linked post-discharge telecoaching
[68] Frazzitta et al., 2012	Parkinson's disease	Return-home/community re-entry evidence	Intensive inpatient rehabilitation with 1-year follow-up	Home exercise carry-over and medication-dose trajectory
[69] Frazzitta et al., 2013	Parkinson's disease	Return-home/community re-entry evidence	MIRT balance/gait 1-year follow-up	Partial gait and balance carry-over
[70] Boesen et al., 2018	Multiple sclerosis	Companion/secondary report	Danish MS Hospitals RCT 6-month report	MDR HRQOL carry-over
[71] Ferrazzoli et al., 2018	Parkinson's disease	Return-home/community re-entry evidence	MIRT quality-of-life RCT	Short and medium-term QoL carry-over
[72] Isernia et al., 2020	Parkinson's disease	Core transition evidence	Clinic-to-home telerehabilitation	HomeHEAD digital rehabilitation continuation
[73] Boesen et al., 2020	Multiple sclerosis	Companion/secondary report	Danish MS Hospitals 12-month report	Long-term HRQOL carry-over
[74] Feldmann et al., 2020	Parkinson's disease	Core transition evidence	Post-discharge PD medication-change study	Medication-continuity and self-adherence risk
[75] Chen et al., 2021	Parkinson's disease	Return-home/community re-entry evidence	Short inpatient MIRT with remote home education	Subtype-specific motor carry-over
[76] Kuendig et al., 2022	Multiple sclerosis	Return-home/community re-entry evidence	Post-rehabilitation real-world PA study	Capacity-to-performance gap
[77] Patt et al., 2023	Multiple sclerosis	Return-home/community re-entry evidence	Inpatient fatigue/self-management RCT	Energy-management education and home exercise booster
[78] Steendam-Oldekamp et al., 2023	Parkinson's disease	Return-home/community re-entry evidence	Advanced PD inpatient/outpatient rehabilitation	ADL stabilization and nursing-home avoidance
[79] Trénel et al., 2024	Multiple sclerosis	Companion/secondary report	Differential MDR effects by main focus area	Goal-setting and durability of HRQOL gains
[80] Gunzler et al., 2025	Parkinson's disease	Return-home/community re-entry evidence	Inpatient mobility and home-to-home discharge	Mobility-readiness for discharge
[81] Alnes et al., 2026	Parkinson's disease	Core transition evidence	mHealth after inpatient rehabilitation	Remote PA/nutrition self-management support
[82] Friedrich et al., 2026	Parkinson's disease	Return-home/community re-entry evidence	PD-MCT prospective follow-up	Multimodal complex-treatment carry-over
[83] Pinto et al., 2010	ALS	Adjacent continuity evidence	Home NIV telemonitoring controlled trial	Remote ventilator review and health-care utilization
[84] De Almeida et al., 2010	ALS	Adjacent continuity evidence	Wireless device feasibility for home ventilation	Remote ventilator data transmission
[85] Vitacca et al., 2010	ALS	Adjacent continuity evidence	Pilot telemedicine-assisted integrated care	Nurse-tutor, oximetry and triage after discharge
[86] Vitacca et al., 2012	ALS	Companion/secondary report	Long-term TAIC activity/cost study	Scalable nurse-led tele-assistance
[87] Creemers et al., 2014	ALS	Adjacent continuity evidence	Case-management cluster RCT	Navigation support added to multidisciplinary care
[88] Bakker et al., 2015	ALS	Companion/secondary report	Qualitative case-management perspectives	Relational case-management value
[89] Cordesse et al., 2015	ALS	Adjacent continuity evidence	Coordinated community ALS network cohort	Specialist-community pathway coordination
[90] Hobson et al., 2019	ALS/MND	Adjacent continuity evidence	TiM feasibility RCT	Home symptom reporting and nurse review
[91] Pulley et al., 2019	ALS	Adjacent continuity evidence	Store-and-forward multidisciplinary telemedicine	Home assessment plus asynchronous team review
[92] Helleman et al., 2020	ALS	Adjacent continuity evidence	ALS home-monitoring and coaching cohort	App self-monitoring and nurse feedback
[93] Capozzo et al., 2020	ALS	Adjacent continuity evidence	COVID-era ALS telemedicine	Structured remote triage and follow-up
[94] Munan et al., 2020	ALS	Adjacent continuity evidence	Qualitative patient-caregiver transition study	Device, dependency, communication and ACP transitions

https://doi.org/10.3390/medsci14040445

Study/reference	Disease population	Evidence category	Rationale for classification	Main transition mechanism
[95] De Marchi et al., 2021	ALS	Adjacent continuity evidence	Multidisciplinary ALS televisits during COVID	Remote neurology, nutrition, psychology and physiotherapy
[96] Dontje et al., 2022	ALS	Companion/secondary report	Nationwide ALS H&C implementation	Scale-up, fidelity and sustainability of home monitoring
[97] Réginault et al., 2023	ALS	Adjacent continuity evidence	At-home NIV initiation cohort	Physiotherapist-led home NIV initiation plus telemonitoring
[98] Fidelix et al., 2023	ALS	Adjacent continuity evidence	Multidisciplinary ALS telehealth cohort	Remote respiratory, medication and gastrostomy management
[99] Bublitz et al., 2024	ALS	Adjacent continuity evidence	Home neuropalliative ALS care	Multiprofessional home visits and end-of-life care
[100] Kim et al., 2025	ALS	Adjacent continuity evidence	Home-based ALS video televisits	Equipment, acute issues and ACP support
[101] Mercadante et al., 2025	ALS	Companion/secondary report	Home palliative-care cohort	Respiratory/nutritional support and shared planning
[102] Schmidt et al., 2025	ALS	Adjacent continuity evidence	Claims-based palliative-care pathway analysis	PPC, SPHC, inpatient and hospice sequencing
[103] Mercadante et al., 2026	ALS	Companion/secondary report	ACP/living will analysis in home palliative care	Relationship-based ACP facilitation
[104] Mercadante et al., 2026b	ALS	Companion/secondary report	Longitudinal home palliative-care follow-up	Symptom, NIV and ACP trajectory monitoring

Abbreviations: 4Ms = What Matters, Medication, Mentation and Mobility; ACP = advance care planning; ADRD = Alzheimer's disease and related dementias; ALS = amyotrophic lateral sclerosis; ALSFRS-R = Amyotrophic Lateral Sclerosis Functional Rating Scale-Revised; BPSD = behavioral and psychological symptoms of dementia; ED = emergency department; ER = emergency room; Fam-FFC = Family-centered Function-focused Care; H&C = Home Monitoring and Coaching; HHA = home health agency; HRQOL = health-related quality of life; MDR = multidisciplinary rehabilitation; MIRT = multidisciplinary intensive rehabilitation treatment; MND = motor neuron disease; NIV = non-invasive ventilation; OASIS = Outcome and Assessment Information Set; PA = physical activity; PD = Parkinson's disease; PPR = potentially preventable readmission; QoL = quality of life; SNF = skilled nursing facility; TAIC = tele-assistance integrated care. Companion/secondary reports were retained for distinct mechanistic, outcome, implementation, or subgroup information but were not treated as independent replication.

ALS classification note: the continuous, progression- or needs-triggered continuity pathway is a descriptive sublabel within adjacent continuity. It does not create a fifth evidence category, change report allocation, or alter any count in Supplementary Table S3.

Supplementary Table S4. Care-model taxonomy across neurological populations

Care model	Operational definition	Disease groups represented	Typical delivery personnel	Typical setting	Examples of outcomes	Implementation considerations
Discharge planning and documentation	Structured preparation for transfer from hospital/SNF to home, PAC, HHA or community services.	Dementia/ADRD, Parkinson's disease	Physicians, nurses, social workers, pharmacists, case managers	Acute hospital, SNF, discharge planning unit	Readmission, ED use, documentation completeness, caregiver understanding	Needs dementia-specific and medication-specific content; generic discharge summaries are insufficient.
Caregiver-mediated transition support	Intervention or process in which caregiver becomes education recipient, proxy, care coordinator or outcome participant.	Dementia/ADRD, ALS, advanced PD	Nurses, dementia specialists, palliative-care staff, social workers	Hospital-to-home, SNF-to-home, home palliative care	Preparedness, burden, strain, medication confidence, satisfaction	Requires explicit measurement of caregiver work and capacity.
Medication/treatment continuity	Medication reconciliation, dose timing, medication list, treatment-plan communication and post-discharge medication monitoring.	Dementia/ADRD, Parkinson's disease	Pharmacists, nurses, physicians, caregivers	Hospital, discharge, home, HHA	Medication changes, errors, adherence, adverse events	PD requires time-critical dosing; dementia requires caregiver-facing medication information.
Home-health/post-acute pathway	Use and quality of home health, SNF, home without services or discharge-to-community pathways.	Dementia/ADRD	HHA clinicians, PT/OT/ST, nurses, aides	Home health, PAC, SNF	PPR, successful discharge to community, therapy completion	Equity, service quality and episode duration influence outcomes.

https://doi.org/10.3390/medsci14040445

Care model	Operational definition	Disease groups represented	Typical delivery personnel	Typical setting	Examples of outcomes	Implementation considerations
Rehabilitation carry-over	Persistence of inpatient or specialist rehabilitation gains after discharge.	Parkinson's disease, multiple sclerosis	Physiotherapists, occupational therapists, rehabilitation physicians, neurologists	Inpatient rehabilitation to home/community	ADL, mobility, walking capacity, fatigue, HRQOL, PA	Requires home practice, coaching, community linkage or behavioral aftercare.
Telehealth/telerehabilitation/mHealth	Remote continuation of follow-up, rehabilitation, self-management or symptom monitoring.	Parkinson's disease, multiple sclerosis, ALS, dementia/ADRD	Nurses, physiotherapists, neurologists, dieticians, psychologists, coaches	Home, clinic-to-home, post-discharge remote care	Feasibility, adherence, QoL, activity, escalation, usability	Digital access and caregiver involvement must be reported.
Respiratory/nutritional continuity	Home monitoring and support for NIV, ventilation, oxygenation, cough, gastrostomy and nutrition.	ALS	Pulmonologists, respiratory therapists, ALS nurses, dieticians, neurologists	Home ventilation, ALS clinic, home palliative care	NIV adherence, hypoxaemia, admissions, symptoms, equipment problems	Respiratory continuity is a core ALS transition mechanism.
Palliative-care and ACP continuity	Transfer of goals-of-care, symptom, hospice and ACP support across settings.	ALS, dementia/ADRD	Palliative-care physicians, nurses, social workers, chaplains, caregivers	Hospital, home palliative care, hospice, HHA	ACP, place of death, hospital use, symptom burden, grief support	Requires repeated conversations and integration with community services.
Integrated or community network care	Formal linkage of specialist teams, primary care, community services and home care.	ALS, Parkinson's disease, dementia/ADRD	Multidisciplinary teams, coordinators, primary care, community services	Specialist center, home, community	Hospitalization, survival, QoL, perceived quality, service use	Benefits depend on network maturity and implementation context.

Abbreviations: ACP = advance care planning; ADL = activities of daily living; ADRD = Alzheimer's disease and related dementias; ALS = amyotrophic lateral sclerosis; ED = emergency department; HHA = home health agency; HRQOL = health-related quality of life; NIV = non-invasive ventilation; PAC = post-acute care; PA = physical activity; PD = Parkinson's disease; PPR = potentially preventable readmission; PT/OT/ST = physical, occupational and speech therapy; QoL = quality of life; SNF = skilled nursing facility.

Supplementary Table S5. Outcome-domain mapping

Outcome domain	Examples of instruments/outcomes	Disease groups represented	Level of reporting consistency	Key gaps
Safety and service use	Readmission, ED/ER use, hospitalizations, ICU use, falls, injuries, death, PPR, emergency calls	Dementia/ADRD, ALS, Parkinson's disease	Moderate but definitions differ	Need standardized follow-up windows and outcome definitions.
Functional recovery and ADL	Barthel, FIM, ALDS, ADL, walking, mobility, transfers, balance, TUG, 6MWT	Parkinson's disease, multiple sclerosis, dementia/ADRD	High in rehabilitation studies; lower in transition studies	Need link between inpatient gains and real-world function.
Medication/treatment continuity	Medication changes, medication errors, DAA use, HMR, caregiver medication confidence, PD timing	Dementia/ADRD, Parkinson's disease	Moderate	Medication outcomes should include patient/caregiver-facing measures.
Caregiver outcomes	Preparedness, burden, strain, anxiety, depression, satisfaction, role ambiguity, confidence	Dementia/ADRD, ALS	Moderate in dementia; variable in ALS	Caregiver work is often implicit and undermeasured.
Quality of life and participation	PDQ-39, FAMS, MSIS-29, SF-36, ALSAQ-40, participation/community re-entry	Parkinson's disease, multiple sclerosis, ALS	Variable	Participation and community re-entry are underreported.

https://doi.org/10.3390/medsci14040445

Outcome domain	Examples of instruments/outcomes	Disease groups represented	Level of reporting consistency	Key gaps
Respiratory/nutritional/palliative outcomes	NIV adherence, SpO2, ventilation settings, gastrostomy, ACP, place of death, palliative pathways	ALS, advanced dementia	Moderate in ALS; emerging in dementia	Need integrate symptom, service-use and goal-concordance outcomes.
Feasibility and implementation	Recruitment, retention, fidelity, adherence, call duration, cost, usability, SUS, adoption, sustainability	ALS, dementia/ADRD, digital PD/MS studies	Moderate	Need report workforce, digital access and implementation burden.
Equity and access	Race/ethnicity, dual eligibility, HHA quality, geographic distance, language/cultural tailoring	Dementia/ADRD, ALS	Low to moderate	Equity should become a standard transition outcome domain.

Abbreviations: ACP = advance care planning; ADL = activities of daily living; ADRD = Alzheimer's disease and related dementias; ALS = amyotrophic lateral sclerosis; ALSAQ-40 = Amyotrophic Lateral Sclerosis Assessment Questionnaire-40; ALDS = Amsterdam Linear Disability Scale; DAA = dose-administration aid; ED = emergency department; ER = emergency room; FAMS = Functional Assessment of Multiple Sclerosis; FIM = Functional Independence Measure; HMR = home medicines review; HRQOL = health-related quality of life; ICU = intensive care unit; MSIS-29 = Multiple Sclerosis Impact Scale-29; NIV = non-invasive ventilation; PA = physical activity; PD = Parkinson's disease; PDQ-39 = Parkinson's Disease Questionnaire-39; PPR = potentially preventable readmission; QoL = quality of life; SF-36 = 36-item Short Form Health Survey; SpO2 = peripheral oxygen saturation; SUS = System Usability Scale; TUG = Timed Up and Go; 6MWT = Six-Minute Walk Test.

Supplementary Table S6. Technology-enabled continuity models

Model	Disease group	Delivery mode	Monitoring target	Caregiver involvement	Feasibility or implementation findings
Telephone follow-up/calls	Dementia/ADRD, ALS	Scheduled or triggered calls	Caregiver status, BPSD, medication, oximetry, symptoms, palliative needs	Central in dementia and ALS	Feasible and low-burden, but requires clear escalation and documentation.
Telehealth/video visits	ALS, Parkinson's disease	Synchronous video or phone visit	Multidisciplinary assessment, equipment, respiratory/nutritional issues, rehabilitation follow-up	Often required for advanced disease	Acceptability generally high in selected samples; access barriers remain.
Telerehabilitation/telecoaching	Multiple sclerosis, Parkinson's disease	Synchronous or asynchronous coaching/platform	Exercise, PA, fatigue, self-management, motor practice	Variable but often important	Adherence high in structured programs; long-term sustainability needs testing.
mHealth/wearable self-monitoring	Parkinson's disease, ALS	Activity trackers, apps, web portals	Physical activity, well-being, body weight, ALS-FRS-R, symptoms	Caregiver may support use in advanced disease	Requires usability, data review workflow and action thresholds.
NIV/ventilator telemonitoring	ALS	Modem or cloud-based ventilator data review	NIV use, leaks, pressures, volumes, AHI, oximetry	Caregiver often assists device use and communication	Can reduce utilization and accelerate home NIV initiation in selected models.
Store-and-forward telemedicine	ALS	Recorded home assessment reviewed asynchronously	Multidisciplinary ALS team recommendations	Caregiver supports visit logistics	Preserves team input while reducing travel burden.

Abbreviations: AHI = apnea-hypopnea index; ADRD = Alzheimer's disease and related dementias; ALS = amyotrophic lateral sclerosis; ALSFRS-R = Amyotrophic Lateral Sclerosis Functional Rating Scale-Revised; BPSD = behavioral and psychological symptoms of dementia; mHealth = mobile health; NIV = non-invasive ventilation; PA = physical activity.

Supplementary Table S7. Caregiver role and transition workload

Disease group	Caregiver function in the transition	Measured caregiver outcomes	Reported barriers	Implications for intervention design
Dementia/ADRD	Medication administrator, safety monitor, proxy informant, service navigator, discharge-plan interpreter, advocate	Preparedness, burden, strain, depression, satisfaction, role ambiguity, medication concerns	Limited discharge communication, complex home services, poor medication information, cultural/language barriers	Design dyad-centered interventions with caregiver identification, training, tailored resources and post-discharge navigation.

https://doi.org/10.3390/medsci14040445

Disease group	Caregiver function in the transition	Measured caregiver outcomes	Reported barriers	Implications for intervention design
Parkinson's disease	Medication-schedule supporter, mobility/falls observer, home-exercise supporter, ADL and nursing-home-avoidance partner	Caregiver outcomes often undermeasured	Medication timing complexity, fluctuations, falls, cognitive/non-motor burden in advanced disease	Include caregiver medication and mobility-readiness roles in discharge and rehabilitation pathways.
Multiple sclerosis	Exercise/self-management supporter, fatigue routine supporter, home adaptation participant, community participation facilitator	Caregiver outcomes rarely primary in mapped studies	Sustaining PA, fatigue management, translating capacity into daily performance	Integrate home-context barriers, participation goals and support networks into rehabilitation aftercare.
ALS	Ventilation/nutrition/equipment supporter, communication partner, care coordinator, ACP participant, palliative-care partner	Caregiver strain, satisfaction, qualitative experience, caregiver end-of-life ratings	Progressive dependency, device decisions, communication loss, social isolation, end-of-life uncertainty	Build caregiver support into home monitoring, respiratory care and palliative pathways from early stages.

Abbreviations: ACP = advance care planning; ADL = activities of daily living; ADRD = Alzheimer's disease and related dementias; ALS = amyotrophic lateral sclerosis; MS = multiple sclerosis; PA = physical activity; PD = Parkinson's disease.

Supplementary Table S8. Methodological limitations and evidence gaps

Limitation/gap	Disease area affected	Examples from included evidence	Implications for future research
Heterogeneous discharge anchors	All disease areas	Acute hospital, SNF, inpatient rehabilitation, clinic and home-care transitions are not equivalent	Future studies should define the transition start point and timing of intervention initiation.
Variable comparator quality	All disease areas	Many studies used usual care, baseline or no comparator	Use comparators that distinguish intervention content from attention, dose and service access.
Outcome heterogeneity	All disease areas	Readmission, HRQOL, function, caregiver and implementation outcomes use different windows and tools	Develop core outcome domains for neurological transition research.
Caregiver work undermeasured	Dementia/ADRD, ALS, advanced PD	Caregiver often performs transition labor but is not always an outcome participant	Measure caregiver preparedness, workload, capacity and equity.
Equity underreported	Most areas except dementia/ADRD home-health studies	Few studies report race/ethnicity, socioeconomic status, language, geography or digital access	Include equity variables and subgroup analyses in transition interventions.
Technology access and usability gaps	Telehealth, mHealth and telemonitoring studies	Selected users may exclude severe cognitive impairment or low digital access	Report accessibility adaptations, caregiver assistance, drop-out and usability.
Companion/secondary report overlap	Fam-FFC, Connect-Home, Danish MS Hospitals, ALS H&C, ALS palliative-care cohorts	Multiple reports enrich mechanisms but may overrepresent programs	Identify overlapping cohorts and avoid double-counting independent evidence.
Limited long-term follow-up	All disease areas	Many studies focus on 30 days, 3 months or 6 months	Use longer follow-up for community stability, participation, caregiver outcomes and sustainability.
No formal critical appraisal or certainty grading	All disease areas	The scoping objective prioritized breadth and evidence mapping; design features were charted descriptively.	Interpret mapped patterns cautiously; focused future effectiveness reviews should use formal risk-of-bias and certainty methods when evidence becomes sufficiently homogeneous.

Abbreviations: ADRD = Alzheimer's disease and related dementias; ALS = amyotrophic lateral sclerosis; H&C = Home Monitoring and Coaching; mHealth = mobile health; PA = physical activity; PD = Parkinson's disease; SNF = skilled nursing facility.

https://doi.org/10.3390/medsci14040445

Supplementary Table S9. Traceability matrix linking components of Supplementary Figures S1 and S2 to included studies.

Figure	Framework component	Operational meaning	Disease population	Representative included studies [Ref.]	Evidence category	Mapped mechanism or finding	Interpretive limitation
S1/S2	Information transfer and discharge documentation	Actionable transfer of diagnoses, treatment changes, risks, referrals and responsibilities.	Dementia/ADRD	Cummings [40]; Kable [42,44]; Saragosa [55]	Core; companion	Plan adequacy, documentation failure and caregiver-held information.	Mostly dementia-specific; no comparative component effect.
S1/S2	Medication or treatment continuity	Reconciliation, accurate timing, supply and understandable instructions after transition.	Dementia/ADRD; PD	Bruce [45]; Sawan [48]; Schmidt [74]; Bublitz [80]	Core; return-home	Caregiver-mediated medication safety and PD medication-change/timing risks.	Heterogeneous designs and endpoints.
S1/S2	Caregiver preparation and dyadic support	Prepare caregivers for treatment, symptoms, daily care and navigation.	Dementia/ADRD; ALS	Boltz [43,57]; Berish [58]; Horstman [60]; Helleman [88]	Core; companion	Preparedness, role clarity, navigation and emotional support.	Caregiver outcomes and follow-up windows varied.
S1/S2	Rehabilitation carry-over	Translate inpatient gains and goals into home performance and participation.	MS; PD	Freeman [21]; Boesen [24]; Frazzitta [68]; Boesen [70,73]; Ferrazzoli [75]; Bublitz [81]	Core; return-home; companion	Functional carry-over, fatigue support, home exercise and coaching.	No shared core outcome set.
S1/S2	Monitoring and escalation	Detect deterioration and activate predefined clinical or service responses.	All; strongest in dementia/ALS	Knox [46,47]; Berish [58]; Drazich [59]; Bublitz [80]; Mercadante [90,92]	Core; return-home; adjacent; companion	Risk stratification, symptom monitoring and escalation prompts.	Thresholds and response pathways were inconsistently reported.
S1/S2	Coordination and accountable linkage	Connect hospital/specialist plans with primary, rehabilitation, home and community services.	Dementia/ADRD; ALS	Cummings [40]; Toles [51]; Creemers [87]; Helleman [89]	Core; adjacent	Case management, rapid linkage and named continuity roles.	Incremental benefit over established multidisciplinary care was uncertain.
S1/S2	Respiratory and NIV continuity	Initiate, monitor and adjust ventilation and respiratory support at home.	ALS	Pinto [83]; De Almeida [84]; Vitacca [85]; Capozzo [95]; Pulley [97]	Adjacent	Remote NIV data, oximetry, adherence review and respiratory escalation.	Mostly non-randomized or feasibility evidence.
S1/S2	Telehealth and digital continuity	Remote monitoring, coaching or consultation between scheduled in-person contacts.	MS; ALS	Boesen [25]; Flachenecker [72]; Mercadante [90-92]; Capozzo [93,95]; Dontje [96]; Pulley [98]; Trenel [100]	Core; return-home; adjacent; companion	Access, self-management, multidisciplinary review and escalation.	Digital access and generalizability were rarely tested as equity outcomes.
S1/S2	Palliative and advance-care-planning continuity	Maintain goals-of-care, symptom support and anticipatory planning across settings.	Dementia/ADRD; ALS	Du Preez [54]; Toles [63]; Alnes [99]; Friedrich [101]; Kuendig [102]; Noorgard [103]; Reginault [104]	Core; adjacent; companion	Planning, symptom support, home death and hospital avoidance.	Referral timing and access differed across systems.
S1/S2	Escalation and urgent-care avoidance	Provide instructions and rapid alternatives to emergency or hospital care.	Dementia/ADRD; ALS	Villars [41]; Vitacca [85]; Mercadante [90,92]; Capozzo [95]; Pulley [97]	Core; adjacent	Telephone triage, risk cards and remote clinical response.	Causal effects cannot be separated from selection and service context.
S1/S2	Implementation and sustainability	Embed models in workflow with acceptable burden, fidelity and ongoing resources.	Dementia/ADRD; MS; ALS	Boltz [43,57]; Boesen [72]; Mercadante [92]; Dontje [96]	Core; return-home; adjacent; companion	Fidelity, workflow integration, usability, adoption and national scale-up.	Costs and long-term sustainability were underreported.

https://doi.org/10.3390/medsci14040445

Figure	Framework component	Operational meaning	Disease population	Representative included studies [Ref.]	Evidence category	Mapped mechanism or finding	Interpretive limitation
S1/S2	Equity and access	Identify differential reach, affordability, service quality and cultural/digital barriers.	Dementia/ADRD; ALS	Cummings [40]; Temkin-Greener [56]; Yang [65]; Greyson [66]; Capozzo [93]; Dontje [96]	Core; return-home; adjacent	Resource affordability, race/ethnicity, dual eligibility, cultural fit and digital context.	Explicit equity analyses were sparse outside dementia/ADRD.

Note: The matrix identifies representative, directly mapped sources rather than treating every listed study as support for every framework element. Evidence categories describe transition proximity or reporting overlap, not methodological quality or certainty.

Supplementary Figure S1. Proposed Conceptual Framework for Post-discharge Neurological Transition Care. This author-derived, hypothesis-generating framework was developed through framework-based synthesis of charted mechanisms and organizes disease context, transition stages, continuity functions, and access barriers. It is not an empirically validated care model, taxonomy, effect-size analysis, or clinical prediction tool.

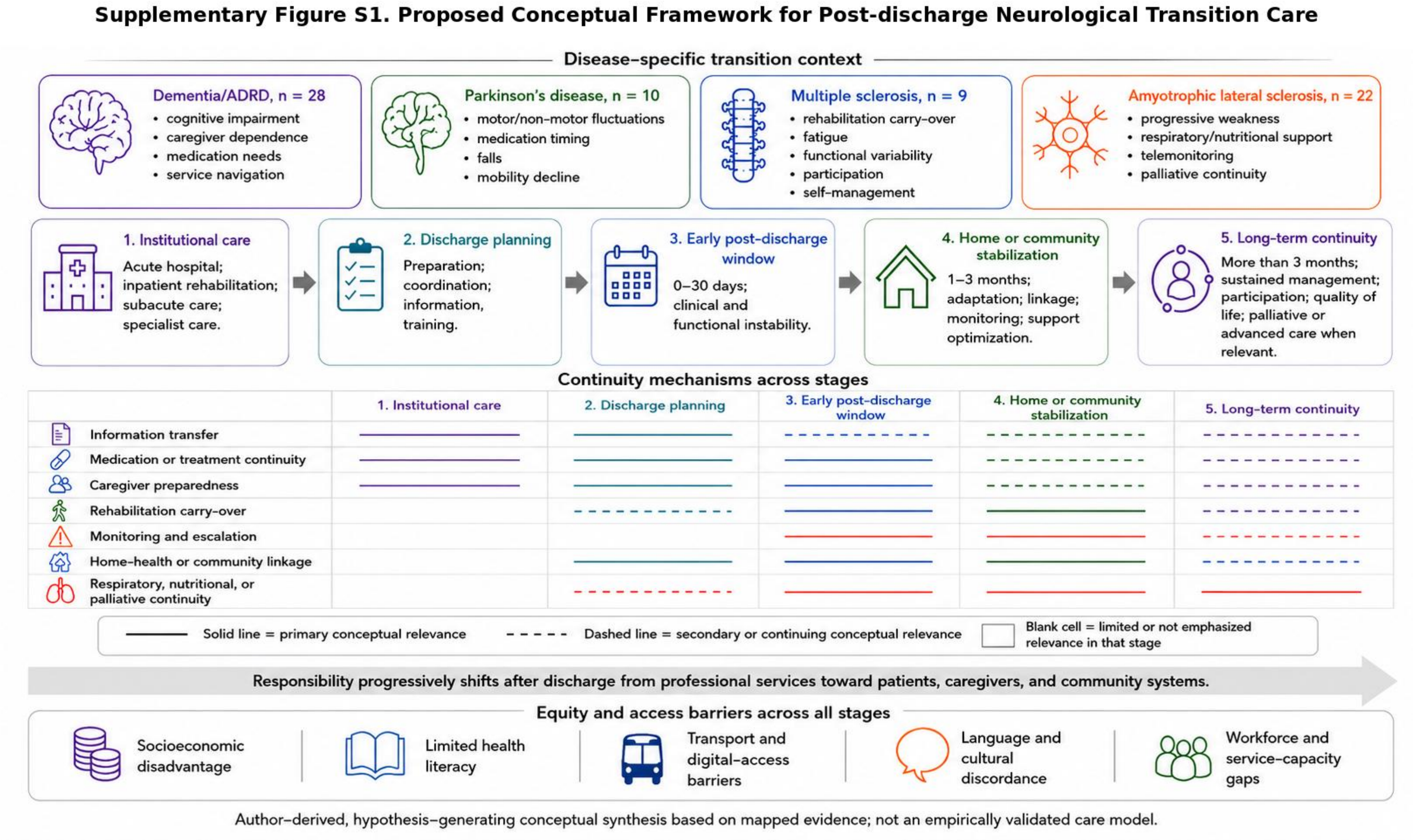

Supplementary Figure S2. Proposed Disease-specific Conceptual Framework of Transition Phenotypes and Care-continuity Mechanisms. This author-derived, hypothesis-generating synthesis links disease-specific risks, candidate continuity mechanisms, mapped outcomes, and evidence gaps. It is not an empirically validated taxonomy, effect-size display, care model, or clinical prediction tool.

Supplementary Figure S2. Proposed Disease-specific Conceptual Framework of Transition Phenotypes and Care-continuity Mechanisms

Disease population	Disease-specific transition phenotype	Key risks during transition	Care-continuity mechanisms	Mapped outcomes	Evidence gaps
**Dementia/ADRD n = 28**	Caregiver-mediated safety and service navigation	• cognitive impairment<br>• medication confusion<br>• behavioral symptoms<br>• caregiver overload<br>• fragmented home-health access	• caregiver preparedness<br>• discharge documentation<br>• medication guidance<br>• SNF-to-home support<br>• home-health linkage<br>• palliative planning	• readmission<br>• emergency department use<br>• caregiver preparedness<br>• medication safety<br>• home-health quality<br>• discharge-to-community success	• long-term dyad outcomes<br>• equity-sensitive implementation<br>• caregiver workload measurement
**Parkinson's disease n = 10**	Medication timing, mobility, and home-to-home stability	• dopaminergic mistiming<br>• falls<br>• delirium<br>• mobility decline<br>• nursing-home risk	• medication reconciliation<br>• inpatient mobility<br>• multidisciplinary rehabilitation<br>• mHealth self-management<br>• outpatient support	• activities of daily living<br>• mobility<br>• home-to-home discharge<br>• quality of life<br>• nursing-home avoidance<br>• medication changes	• standardized medication-safety pathways<br>• long-term home stability<br>• pragmatic implementation
**Multiple sclerosis n = 9**	Rehabilitation carry-over, fatigue, and self-management	• fatigue relapse into inactivity<br>• loss of inpatient rehabilitation gains<br>• functional variability<br>• limited participation	• inpatient-to-home rehabilitation carry-over<br>• telecoaching<br>• digital aftercare<br>• fatigue management<br>• self-management support	• health-related quality of life<br>• fatigue<br>• walking capacity<br>• physical activity<br>• participation<br>• self-efficacy	• capacity-to-performance translation<br>• long-term behavioral maintenance<br>• standardized participation outcomes
**Amyotrophic lateral sclerosis n = 22**	Respiratory, nutritional, telehealth, and palliative continuity	• respiratory decline<br>• non-invasive ventilation adaptation<br>• dysphagia and nutritional support<br>• caregiver escalation<br>• end-of-life decisions	• non-invasive ventilation telemonitoring<br>• home ventilation support<br>• multidisciplinary telehealth<br>• home palliative care<br>• advance care planning	• non-invasive ventilation adherence<br>• hospital use<br>• emergency visits<br>• symptom burden<br>• home death<br>• caregiver satisfaction<br>• advance care planning	• timing of palliative referral<br>• integration of telemonitoring with escalation<br>• equitable access to home specialist care

**Shared continuity mechanisms:**	information transfer; medication or treatment continuity; caregiver preparedness; rehabilitation carry-over; monitoring and escalation; home-health or community linkage; respiratory, nutritional, or palliative continuity; equity and access
**Mapped outcome domains:**	readmission or emergency department use; safety; activities of daily living or mobility; fatigue or health-related quality of life; caregiver preparedness; service use; home or community stability; palliative or advance-care-planning outcomes; equity or access

Author-derived synthesis from mapped evidence; disease-specific pathways are hypothesis-generating. Not an effect-size display and not an empirically validated taxonomy.

https://doi.org/10.3390/medsci14040445
